# The effect of metabolism-related lifestyle and clinical risk factors on digestive system cancers in East Asian populations: a two-sample Mendelian randomization analysis

**DOI:** 10.1038/s41598-024-60122-6

**Published:** 2024-04-24

**Authors:** Xianlei Cai, Xueying Li, Chao Liang, Miaozun Zhang, Zhebin Dong, Weiming Yu

**Affiliations:** 1 Department of Gastrointestinal Surgery, Ningbo Medical Center Lihuili Hospital, The Lihuili Affiliated Hospital, Ningbo University, Ningbo, 315000 Zhejiang China; 2 Department of Gastroenterology, First Affiliated Hospital, School of Medicine, Zhejiang University, Hangzhou, 310003 China; 3 Department of Gastroenterology, Ningbo First Hospital, Ningbo, 315000 Zhejiang China

**Keywords:** Digestive system cancers, Risk factor, Mendelian randomization, Lifestyle, Physical conditions, Metabolic comorbidities, Cancer, Gastroenterology, Oncology, Risk factors

## Abstract

Metabolic factors play a critical role in the development of digestive system cancers (DSCs), and East Asia has the highest incidence of malignant tumors in the digestive system. We performed a two-sample Mendelian randomization analysis to explore the associations between 19 metabolism-related lifestyle and clinical risk factors and DSCs, including esophageal, gastric, colorectal, hepatocellular, biliary tract, and pancreatic cancer. The causal association was explored for all combinations of each risk factor and each DSC. We gathered information on the instrumental variables (IVs) from various sources and retrieved outcome information from Biobank Japan (BBJ). The data were all from studies of east Asian populations. Finally, 17,572 DSCs cases and 195,745 controls were included. Our analysis found that genetically predicted alcohol drinking was a strong indicator of gastric cancer (odds ratio (OR) = 0.95; 95% confidence interval (CI): 0.93–0.98) and hepatocellular carcinoma (OR = 1.11; 95% CI: 1.05–1.18), whereas coffee consumption had a potential protective effect on hepatocellular carcinoma (OR = 0.69; 95% CI: 0.53–0.90). Triglyceride was potentially associated with a decreased risk of biliary tract cancer (OR = 0.53; 95% CI: 0.34–0.81), and uric acid was associated with pancreatic cancer risk (OR = 0.59; 95% CI: 0.37–0.96). Metabolic syndrome (MetS) was associated with esophageal and gastric cancer. Additionally, there was no evidence for a causal association between other risk factors, including body mass index, waist circumference, waist-to-hip ratio, educational levels, lipoprotein cholesterol, total cholesterol, glycine, creatinine, gout, and Graves’ disease, and DSCs. The leave-one-out analysis revealed that the single nucleotide polymorphism (SNP) rs671 from the ALDH2 gene has a disproportionately high contribution to the causal association between alcohol drinking and gastric cancer and hepatocellular carcinoma, as well as the association between coffee consumption and hepatocellular carcinoma. The present study revealed multiple metabolism-related lifestyle and clinical risk factors and a valuable SNP rs671 for DSCs, highlighting the significance of metabolic factors in both the prevention and treatment of DSCs.

## Introduction

Digestive system cancers (DSCs) are a remarkable public health concern globally, affecting a vast number of individuals annually. DSCs involve pronounced heterogeneity and a deficient therapeutic response^1,2^. Various well-known DSCs are esophageal cancer (EC), gastric cancer (GC), colorectal cancer (CRC), hepatocellular carcinoma (HCC), biliary tract cancer (BTC), and pancreatic cancer (PC). Of these cancers, CRC is the primary cause of mortality, accounting for an estimated 9.4% of all cancer-related fatalities, followed by liver cancer (8.3%), stomach cancer (7.7%), esophageal cancer (5.5%), and pancreatic cancer (4.7%). These outcomes are associated with severe and life-threatening implications^3^.

DSCs are linked to various risk factors such as lifestyle, tobacco use, alcohol drinking, obesity, and a family history of the disease. Metabolic risk factors including glucose and lipid metabolism may also contribute to an increased risk of DSCs^1,4^ and are thought to play an important role in cancer development by affecting the expression of genes involved in the regulation of cell growth, differentiation, and apoptosis^5–7^. Tran et al.^8^ reported that metabolic syndrome (MetS) is a risk factor for CRC development, while Hong et al. demonstrated that high variability in visit-to-visit fasting plasma glucose levels was independently associated with an increased risk of GC^9^. Chen et al.^10^ suggested that abnormalities in the regulation and expression of lipid metabolism-related genes accelerated HCC progression. Nevertheless, the role of metabolic risk factors in the development of DSCs remains incompletely understood, and the reported findings are controversial.

Genome wide association studies (GWAS) are used for analyzing a vast amount of genetic data from thousands of individuals to identify genetic variants associated with a specific trait or disease. In this approach, the genetic profiles of individuals with and without the trait or disease are compared, and genetic variants that increase the risk or provide protection from the trait or disease are identified.

Mendelian randomization (MR) is a robust epidemiological method that assesses causal relationships between exposures and outcomes. MR relies on the notion that genetic variants are randomly allocated at conception, that the allocation is not affected by environmental or other factors, and that these variants can be used as instrumental variables (IVs) to estimate the causal effects of exposures on outcomes without being affected by confounding or reverse causation. In recent years, the combination of MR and GWAS has become increasingly popular because of its advantages over traditional epidemiological methods. The combination method can provide unbiased estimates of the causal effect of exposures on outcomes.

The present study investigated the causal relationship between 19 potential metabolism-related lifestyle and clinical risk factors and DSCs using two-sample MR analysis. Findings on the relationship between metabolism-related lifestyle and clinical risk factors and DSCs are expected to aid in improving strategies for cancer prevention and treatment.

## Methods

### MR design

**Figure 1:**
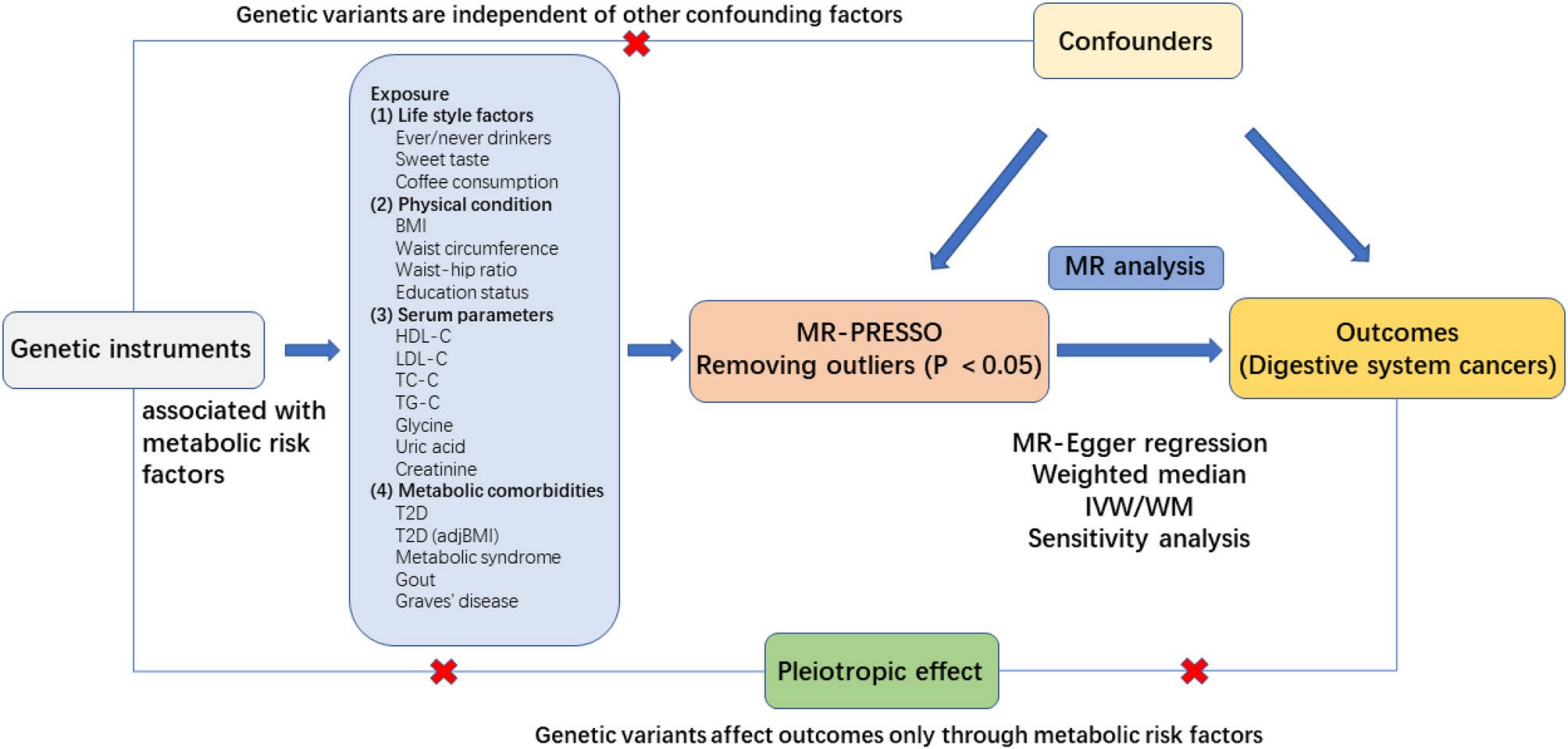
Flowchart of the data collection, processing, and analysis procedures of this study.

This study was conducted according to the STROBE-MR guidelines^11,12^. MR was designed based on three assumptions: (1) Relevance assumption: genetic variants are significantly associated with metabolism-related lifestyle and clinical risk factors; (2) Independence assumption: genetic variants are independent of other confounding factors for DSCs; (3) Exclusion restriction assumption: genetic variants affect DSCs only through metabolism-related lifestyle and clinical risk factors^13^ (Fig. 1). Due to the wide variety of metabolism-related lifestyle and clinical risk factors, we have categorized the 19 risk factors into 4 distinct groups to aid in clarity and comprehension. Overall, 19 predominant metabolism-related lifestyle and clinical risk factors were selected and divided into the following four groups: lifestyle factors, physical factors, serum parameters, and metabolic comorbidities. The associations between these metabolism-related lifestyle and clinical risk factors and the development of DSCs (including EC, GC, CRC, HCC, BTC, and PC) were explored preliminarily.

### Selection of genetic variants

In MR analysis, we used IVs to investigate the potential associations between metabolism-related lifestyle and clinical risk factors and the development of DSCs. The risk fators/traits were classified into four categories: (1) lifestyle factors, namely, ever/never drinking alcohol, sweet taste, and coffee consumption; (2) physical factors, namely, body mass index (BMI), waist circumference (adjusted by BMI), waist-to-hip ratio (adjusted by BMI), and educational level; (3) serum parameters, namely, high-density lipoprotein cholesterol (HDL-C), low-density lipoprotein cholesterol (LDL-C), total cholesterol (TC), triglyceride (TG), glycine, uric acid, and creatinine levels; and (4) metabolic comorbidities, namely, type 2 diabetes (T2D), T2D (adjusted by BMI), MetS, gout, and Graves' disease.

To obtain GWAS summary data for these traits, we collected information on the IVs from various sources on populations of east Asian ancestry (Supplementary Table 1). The IVs for BMI, uric acid, creatinine, gout, and Graves' disease were retrieved from Biobank Japan (BBJ) release of disease traits through the Integrative Epidemiology Unit (IEU) Open GWAS project^14^, and the IVs for HDL-C, LDL-C, TC, TG, and T2D^15^ were retrieved from the Asian Genetic Epidemiology Network (https://blog.nus.edu.sg/agen/). Additionally, the IVs for ever/never drinking alcohol and coffee consumption were extracted from the study of Matoba et al.^16^, the IVs for sweet taste were extracted from the study of Kawafune et al.^17^, the IVs for waist circumference and waist-to-hip ratio were extracted from the study of Wen et al.^18^, the IVs for educational level were extracted from the pan-ancestry genetic analysis of UK Biobank performed at the Broad Institute (https://gwas.mrcieu.ac.uk/datasets/ukb-e-845_EAS/). The dataset (ukb-e-845_EAS) was collected within participants of east Asian ancestry. The IV for glycine was extracted from the study of Chang et al.^19^, the IVs for MetS were extracted from the study of Zhu et al.^20^, and the IVs for gout were extracted from the study of Nakayama et al.^21^.

To ensure the quality of the instrumental single nucleotide polymorphisms (SNPs) of the risk factors, a series of quality control measures were established. First, we identified SNPs were significantly associated with risk factors at the traditional threshold (*P* < 5 × 10^–8^). Second, we removed SNPs with a minor allele frequency (MAF) of < 0.01. Third, we retained only SNPs with a long physical distance (window size = 10,000 kb) and a low likelihood of linkage disequilibrium estimates (r^2^ < 0.001) from East Asian samples in the 1000 Genomes Project. Fourth, the proportion of variance explained by the SNPs (PVE) was calculated according to the following formula: *PVE* = *2* × *MAF* × *(1-MAF)* × *beta*^2^^22^. The strength of each SNP was measured by calculating the F-statistic using the following formula: *F* = *PVE* × *(N−* *2)/(1−* *PVE)* ^22^. When MAFs were not available in the original studies, another formula was used for calculation (*F* = *beta*^2^*/se*^*2*^)^23^. A statistical power of *F* > 10 was considered to indicate a strong association^13^.

Finally, we separately analyzed 19 eligible risk factors. An overview of these genetic tools is provided in Supplementary Table 1. We also excluded any candidate genetic tools that failed in the MR pleiotropy residual sum and outlier (MR-PRESSO) test (*P* < 0.05)^24^. Ethical approval was obtained in the original studies.

### GWAS summary statistics of DSCs

To ensure comparability in patients’ ancestry, GWAS summary statistics of the associations between genetic variants and the development of EC (1300 cases and 195,745 controls), GC (6563 cases and 195,745 controls), CRC (7062 cases and 195,745 controls), HCC (1866 cases and 195,745 controls), BTC (339 cases and 195,745 controls), and PC (442 cases and 195,745 controls) were retrieved from BBJ, a biobank containing the data of ~ 200,000 participants recruited mainly from 12 medical institutions in Japan during 2003–2008^14,25^. In the IEU open GWAS platform, the GWAS ID corresponding to EC, GC, CRC, HCC, BTC, and PC was “bbj-a-117,” “bbj-a-119,” “bbj-a-107,” “bbj-a-158,” “bbj-a-92,” and “bbj-a-140,” respectively. During the process of extracting SNPs linked to the exposure from the outcome, any SNPs lacking pertinent details in the outcome were excluded^26^.

### Participant overlap in MR analysis

As participant overlap in MR analysis can lead to inflated type I errors^27^, we attempted to select IVs from sources other than BBJ to avoid the introduction of bias due to sample overlap. However, we identified genetic variants in certain risk factors, such as BMI, uric acid, creatinine, and Graves' disease only from BBJ. Potential bias introduced by participant sample overlap was calculated using the following formula: *bias* = *βr/F*, where *β* is the MR estimate, *r* is the sample overlap rate between the exposure and the outcome, and *F* is the mean *F* statistic averaged across IVs^27^.

### Statistical analysis

We prepared a flowchart to perform MR in a step-by-step manner. First, we harmonized the GWAS data of risk factors and DSCs with the selected IVs being a matching index. Second, we used the MR-PRESSO approach to detect pleiotropic outliers among the selected IVs and removed them before MR analysis. Third, we performed MR-Egger regression to test for horizontal pleiotropy, where *P* > 0.05 indicated no evidence of horizontal pleiotropy^28^. In addition, this study aimed to confirm the association between the IVs incorporated in the analysis and the significant risk factors for cancer such as smoking and a family history of all malignant neoplasms. To accomplish this confirmation, we employed an online tool known as PhenoScanner V2^29^. If any IVs were found to be associated with either smoking or a family history of malignant neoplasms, we eliminated them and repeated the MR analysis.

After excluding pleiotropy, we used Cochran's Q test to detect any heterogeneity between SNPs, and various MR methods were used to ensure consistency in the directions (i.e., MR-Egger regression, weighted median, inverse variance weighted [IVW; random-effects models], and weighted mode [WM]). Scatter plots were constructed to visualize the results. For sweet taste, waist-to-hip ratio, glycine, and MetS, only the Wald ratio (WR) method was used (two or fewer SNPs). We calculated the odds ratios (ORs) and the corresponding 95% confidence intervals (CIs) of DSCs per one-standard deviation (SD) increment of a quantitative exposure or per unit change on the log odds scale of binary exposure^24^. Finally, the leave-one-out sensitivity test was used to evaluate the robustness of the IVW estimates and detect the potential influential SNPs. The statistical power of MR was estimated using mRnd^30^.

We applied the Bonferroni-corrected significance level of *P* < 2.63 × 10^–3^ (0.05/19), which indicated a strong association, and a *P* value between 2.63 × 10^–3^ and 0.05 indicated a suggestive association. All analyses were conducted using the TwoSampleMR function of the R package (version 4.0.3).

### Ethics approval and consent to participate

Our analyses were based on publicly available data that have been approved by relevant review boards and no additional ethical approval and consent to participate is required.

## Results

### Baseline participant characteristics

**Figure 2:**
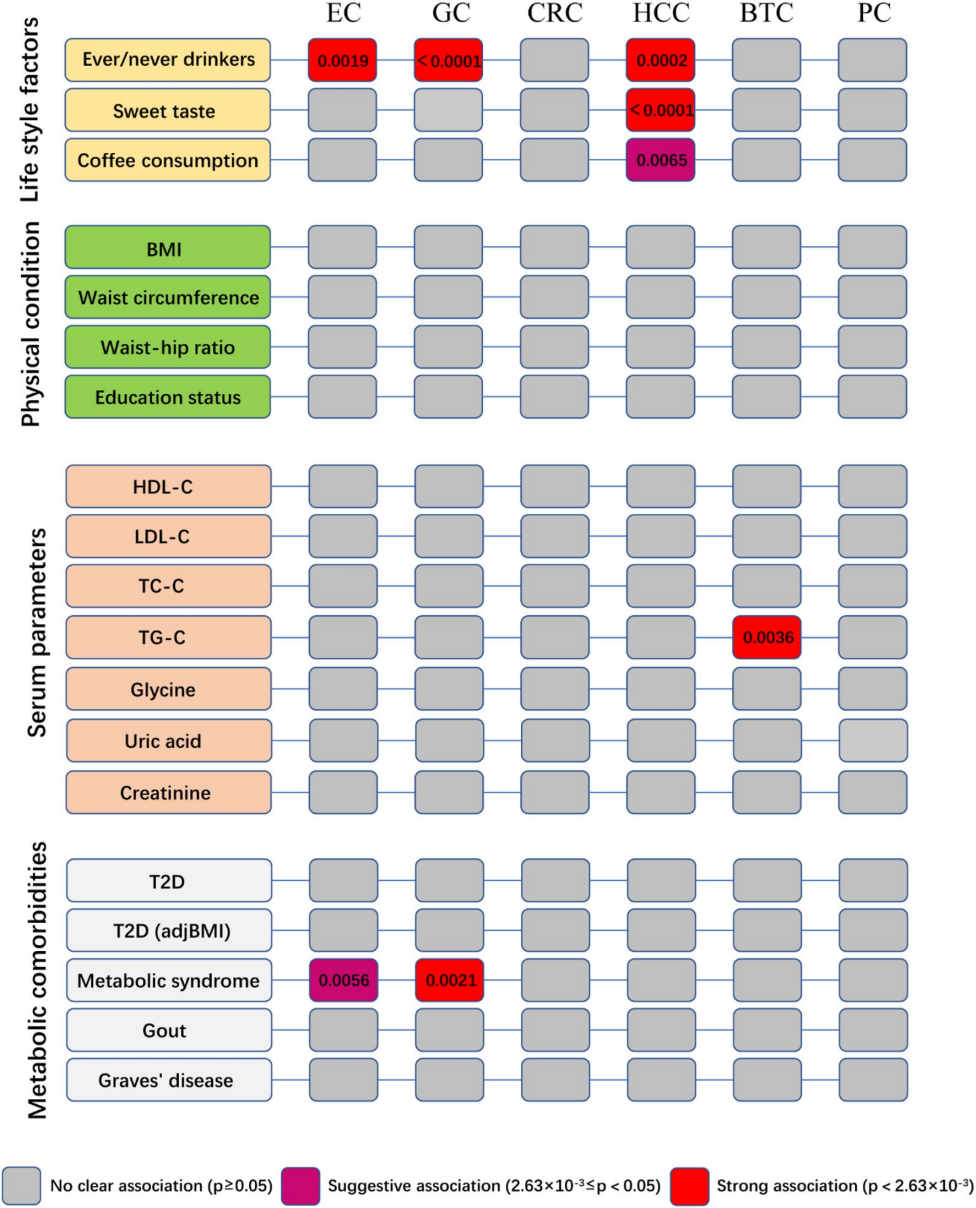
Summary results of this study.

We evaluated 19 possible metabolism-related lifestyle and clinical risk factors to investigate their causal relationships with the development of DSCs. These 19 risk factors were categorized into four groups: lifestyle factors, physical factors, serum parameters, and metabolic comorbidities. Particularly, six types of DSCs (including EC, GC, CRC, HCC, BTC, and PC) were analyzed as outcomes (Fig. 2). The number of SNPs analyzed ranged from 1 to 151. The *F* statistics for all selected traits exceeded 10, indicating the absence of potential weak instrument bias (Supplementary Table 1). Participant overlap between GWAS of BMI, uric acid, creatinine, and Graves' disease and DSCs ranged from 0.001 to 0.06, introducing minimal bias to our causal effect estimates (Supplementary Table 2).

Scatter plots for MR analyses are presented in Supplementary Figs. 1–16. Additionally, the results of the MR-PRESSO test, horizontal pleiotropy test, heterogeneity test, and three MR methods (MR-Egger regression, weighted median, and IVW*/*WR) are presented in Supplementary Tables 3–8. The statistical power of IVs for digestive system cancers are presented in Supplementary Table 9. To adhere to the third MR assumption and prevent any violations, we specifically selected SNPs related to six types of DSCs. This selection was done to confirm that there were no overlaps with IVs for distinct exposures, including but not limited to rs671 and rs1260326 (Supplementary Table 10). The results of the leave-one-out sensitivity test are displayed in Supplementary Figs. 17–32. In the sensitive analysis, we excluded rs11030100, the only IV found to be associated with past tobacco smoking, and repeated the MR analysis. No IVs were found to be associated with a family history of malignant neoplasms.

### Association between risk factors and EC development

**Figure 3:**
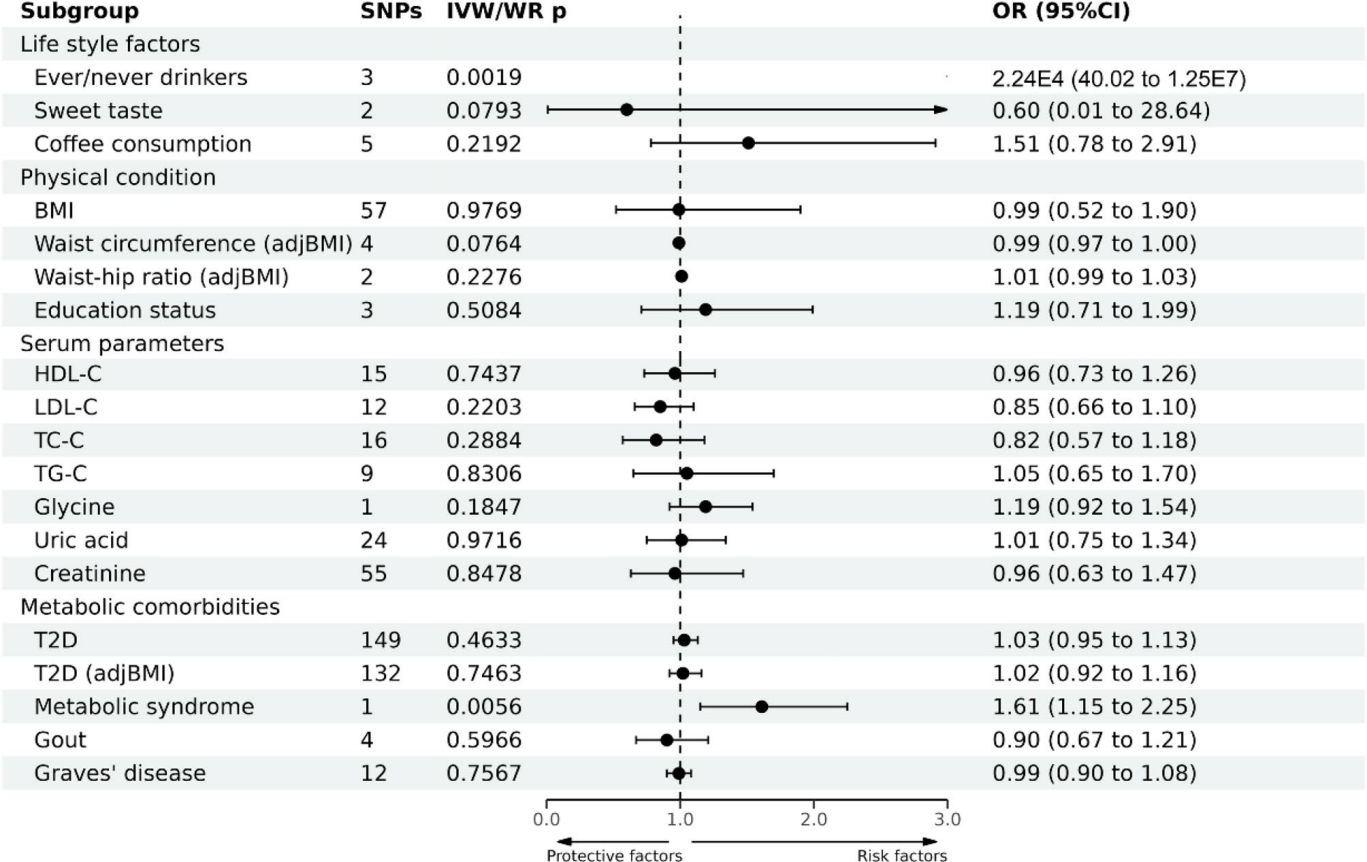
Causal effects of 19 metabolic risk factors on EC estimated by MR analyses.

Regarding lifestyle factors, the available evidence suggested an increased risk of EC in individuals who were ever drinker of alcohol. However, no significant causal association was observed between sweet taste or coffee consumption and EC (Fig. 3 and Supplementary Table 3).

Regarding physical factors and serum parameters, no significant causal associations existed between EC and BMI, waist circumference (adjusted by BMI), waist-to-hip ratio (adjusted by BMI), educational level, HDL-C, LDL-C, TC, TG, glycine, uric acid, or creatinine levels (Fig. 3).

Regarding metabolic comorbidities, the available evidence suggested an association between MetS and EC, with an OR (95% CI) of 1.61 (1.15–2.25, *P* = 0.006) according to the IVW method. No significant causal association existed between T2D, T2D (adjusted by BMI), gout, or Graves’ disease and EC (Fig. 3). The results of the leave-one-out sensitivity test demonstrated that our results were robust.

### Association between risk factors and GC development

**Figure 4:**
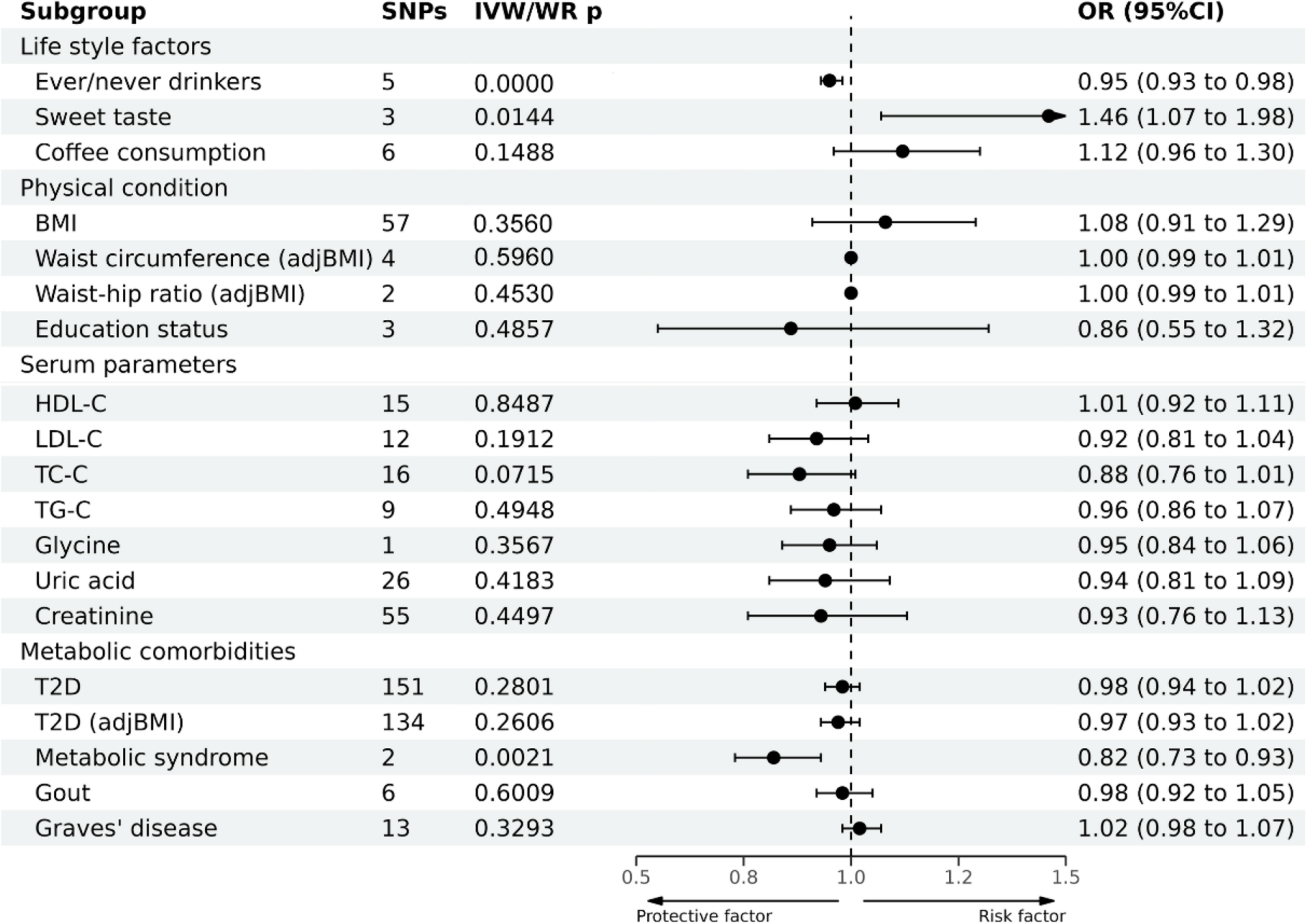
Causal effects of 19 metabolic risk factors on GC estimated by MR analyses.

As for lifestyle factors, a strong causal association between ever drinking alcohol and GC development was noted (OR = 0.95; 95%CI: 0.93–0.98), whereas sweet taste had a suggestive causal association with GC (OR = 1.46; 95%CI: 1.07–1.98; Fig. 4 and Supplementary Table 4). However, the results of the sensitivity analysis revealed that the SNP "rs671" exerted extreme effects on MR results. After excluding rs671, the causal associations between alcohol drinking or sweet taste and GC disappeared (Supplementary Fig. 17B and Supplementary Fig. 18A).

As for metabolic comorbidities, a strong association existed between MetS and GC. However, only two SNPs were included in the MR analysis. No causal association found between the other risk factors and GC. The leave-one-out sensitivity test indicated robust results.

### Association between risk factors and CRC development

**Figure 5:**
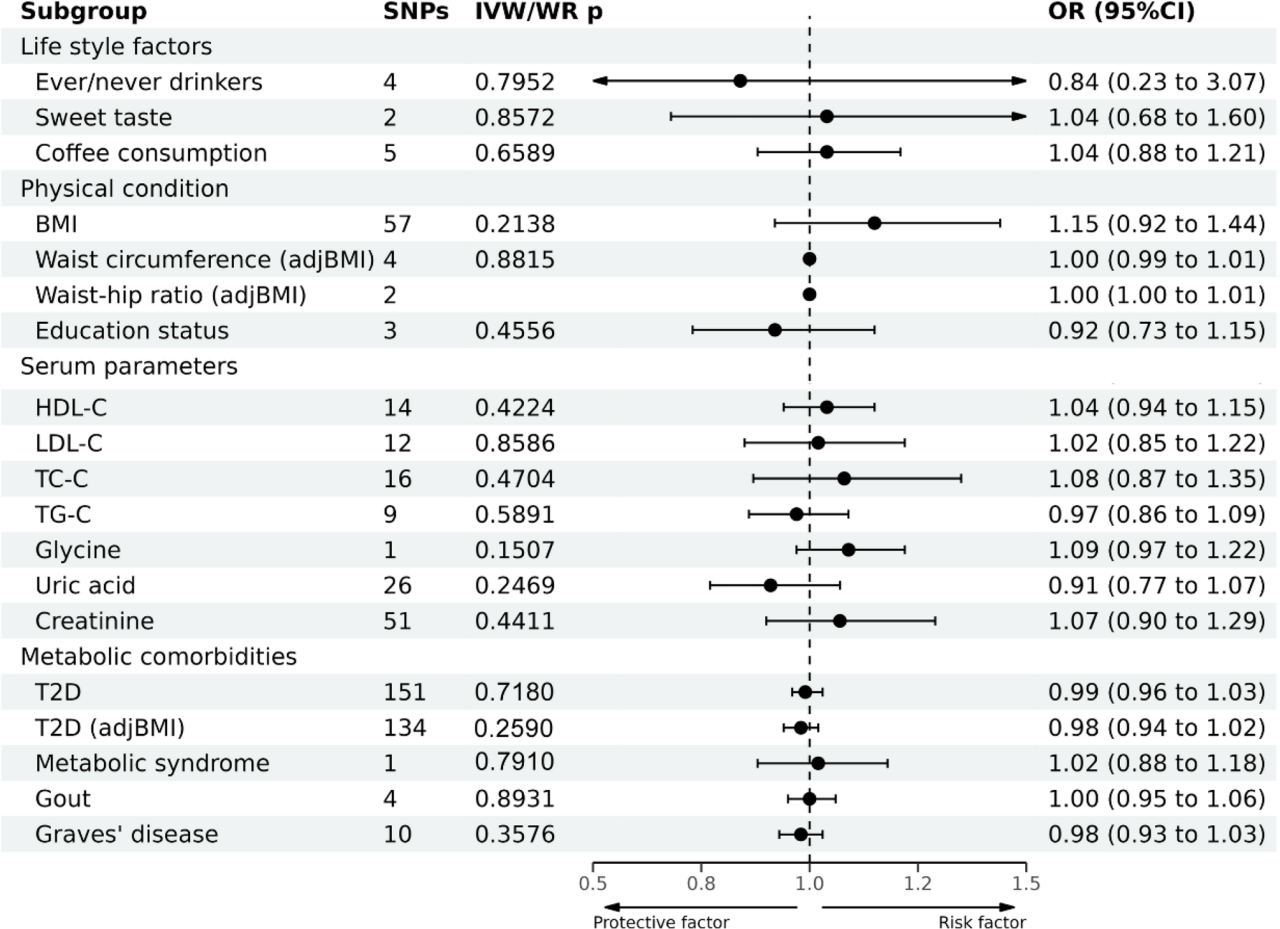
Causal effects of 19 metabolic risk factors on CRC estimated by MR analyses.

MR analysis revealed that no causal association existed between the 19 metabolism-related lifestyle and clinical risk factors and CRC development (Fig. 5 and Supplementary Table 5). The leave-one-out sensitivity test indicated robust results.

### Association between risk factors and HCC development

**Figure 6:**
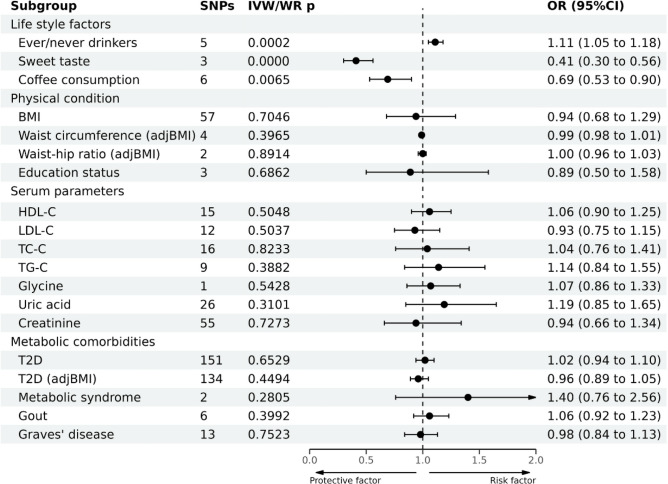
Causal effects of 19 metabolic risk factors on HCC estimated by MR analyses.

As for lifestyle factors, alcohol drinking and sweet taste had a pronounced causal link with HCC development (OR = 1.11; 95% CI: 1.05–1.18 for alcohol drinking; OR = 0.41; 95% CI: 0.30–0.56 for sweet taste), whereas coffee consumption exhibited a suggestive causal relationship with HCC (OR = 0.69; 95% CI: 0.53–0.90; Fig. 6 and Supplementary Table 6). However, sensitivity analysis indicated that the SNP "rs671" had a profound effect on the MR results. After the exclusion of rs671, the causal relationships between alcohol drinking or coffee consumption and HCC were nullified (Supplementary Fig. 17D and Supplementary Fig. 18D). Regarding the other risk factors, no causal association with HCC was noted. The leave-one-out sensitivity test indicated robust results.

### Association between risk factors and BTC development

**Figure 7:**
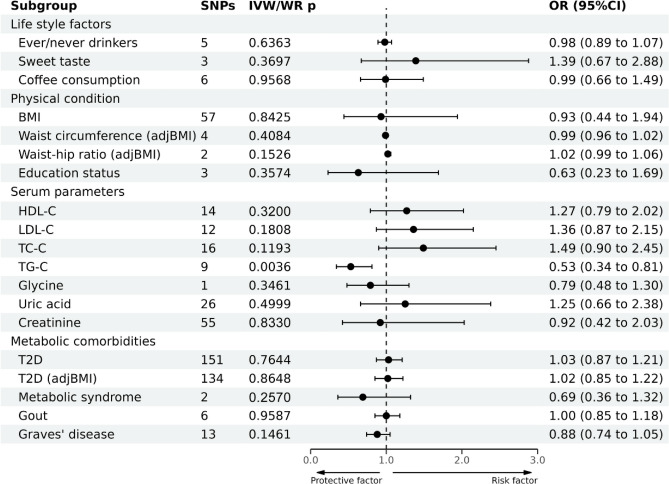
Causal effects of 19 metabolic risk factors on BTC estimated by MR analyses.

The available evidence suggested that genetically predicted higher levels of TG were associated with a reduced risk of BTC (OR = 0.53; 95% CI: 0.34–0.81) for a one-SD increase (Fig. 7 and Supplementary Table 7). However, the leave-one-out sensitivity test results revealed that the SNP "rs662799" had a profound effect on MR results. After the exclusion of rs662799, the causal relationship between TG and BTC was nullified (Supplementary Fig. 26E). The leave-one-out sensitivity test also showed that the SNP "rs10119" had a significant effect on the MR analysis results for the association between LDL and BTC. After the exclusion of rs10119, LDL was suggestively associated with an increased risk of BTC (Supplementary Fig. 24E). No causal association was identified between the other risk factors and BTC development.

### Association between risk factors and PC development

**Figure 8:**
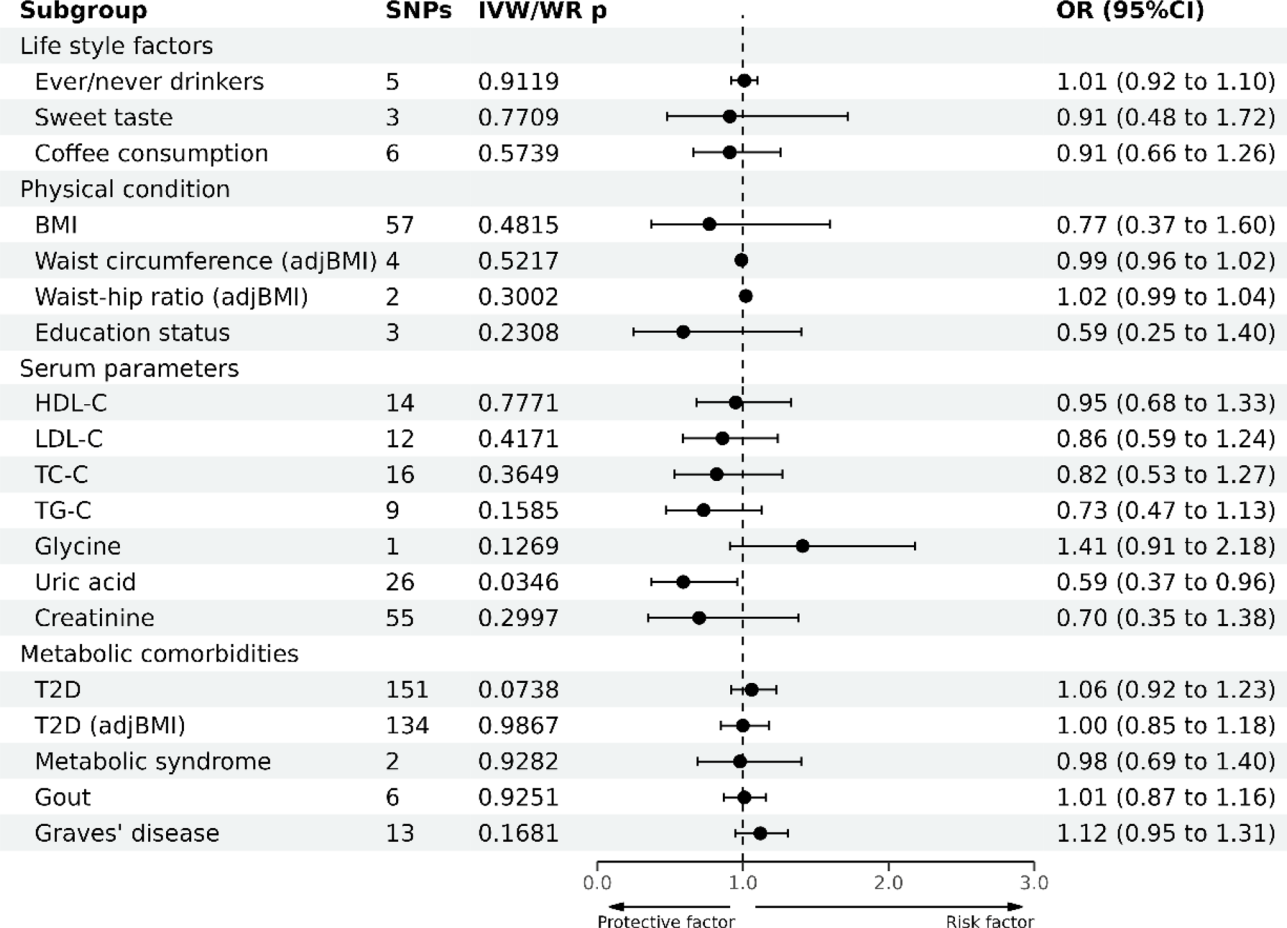
Causal effects of 19 metabolic risk factors on PC estimated by MR analyses.

MR analysis revealed that no causal association existed between the 18 metabolism-related lifestyle and clinical risk factors and PC development except uric acid (Fig. 8 and Supplementary Table 8). The leave-one-out sensitivity test results showed that the SNP "rs671" had an extreme effect on the MR analysis for the association between alcohol drinking and PC. After excluding rs671, a suggestive causal association between alcohol drinking and PC development was observed (Supplementary Fig. 17F).

## Discussion

In this study, we analyzed the causal effect of 19 metabolism-related lifestyle and clinical risk factors on the risk of DSCs through MR analyses. To the best of our knowledge, this study has evaluated the largest number of causal risk factors for DSCs. The present study showed that genetically predicted alcohol drinking is an important indicator strongly associated with GC and HCC development, whereas sweet taste and coffee consumption have potential associations with HCC. TGs were associated with a decreased risk of BTC, and uric acid was associated with PC risk. MetS was associated with EC and GC risk. There was no evidence for a causal association between other risk factors, including BMI, waist circumference, waist-to-hip ratio, educational level, LDL, TC, glycine, creatinine, gout, and Graves’ disease, and the development of DSCs. These findings are essential for providing better treatment for patients with DSCs through early intervention of metabolic risk factors.

Epidemiological investigations have revealed that alcohol drinking is an autonomous risk factor for various types of malignancy. Kubo et al.^31^ reported that both heavy and light drinkers of alcohol had higher probabilities of developing EC, with the former being at a significantly greater risk. Park et al.^32^ discovered that continuous intake of alcohol beyond a low threshold was linked to an increased hazard of liver cancer, consistent with the findings of our MR analysis. Deng et al. performed a dose–response meta-analysis^33^ and demonstrated that increased daily intake of alcohol was correlated with a heightened incidence of GC. Nevertheless, our research showed converse results, revealing that ever drinking alcohol was significantly linked to a diminished hazard of GC (OR = 0.95; 95% CI: 0.93–0.98). Although this result contradicts our prior knowledge, we derived a plausible explanation through our sensitivity analysis. The SNP "rs671" had a substantial effect on the MR results. After rs671 was removed, the causal correlations between alcohol drinking and GC or HCC vanished. Aldehyde dehydrogenase 2 (ALDH2) is an important enzyme with notable function, and it safeguards the cells from acetaldehyde toxicity. ALDH2 ∗ 2 polymorphism (rs671) is the most prevalent SNP in the Asian population. Zhang et al.^34^ elaborated on the prospective mechanisms of ALDH2 ∗ 2 in tumorigenesis and progression. The MR analysis performed in the present study revealed the presence of rs671 and the antithetical causal relationship between several risk factors and DSCs. These results suggested that an individual's SNP could influence the effect of risk factors on the development of cancers. Mechanically, SNPs in different regions can impact gene expression^35,36^. Nonsynonymous coding SNPs in the coding sequence region directly modify the amino acid composition of the protein encoded by the gene^37^. The function of this protein depends on whether the variant amino acid site plays a crucial role in protein structure or function. SNPs in introns mostly influence gene function by altering splice site activity. Gene regulatory regions consist of promoter regions, enhancer regions, and other elements that regulate gene expression. Changes in SNPs at these loci can result in variations in binding ability with regulatory factors, thus affecting normal gene expression. Changes in the expression levels of core genes can impact the susceptibility of the human body to various diseases, leading to the development of different health conditions.

Sweet taste has been linked to the development of various cardiometabolic ailments and is regarded as a risk factor for specific tumor types^38–40^. The present research showed that genetically predicted sweet taste was associated with an increased possibility of GC, whereas the correlation between sweet taste and HCC development showed a converse outcome. After rs671 was removed, the causal correlations between sweet taste and GC or HCC development disappeared, indicating that rs671 had a significant influence on the MR results. Llaha et al.^41^ discovered that the intake of sweetened beverages was not linked to the risk of CRC or PC development, consistent with our findings. However, Liu et al.^42^ performed an MR study and identified a positive association between the intake of sweetened beverages and colon cancer risk, but no causal association between the intake of sweetened beverages and rectal cancer or CRC was observed. The positive association was limited (OR = 1.01; 95% CI: 1.00–1.03), and we speculate that this association could be explained by disparities in ethnicity and tumor classification.

Investigation into the correlation between coffee consumption and cancer risk has been a topic of interest in the past few decades. Zhang et al.^43^ identified an inverse association between coffee consumption and EC development in East Asian participants, but not in Euro-America participants. Xie et al.^44^ reported that individuals in the highest category of coffee consumption may have a decreased risk of GC when compared with non-coffee drinkers. Nonetheless, our MR analysis indicated no causal association between coffee consumption and the risks of EC and GC. Kennedy et al.^45^ demonstrated that coffee intake was inversely associated with the risk of HCC, while Zhao et al.^46^ confirmed this finding through a dose–response analysis. The present study also revealed a protective causal association between coffee consumption and HCC. By contrast, Sartini et al.^47^ did not identify any significant correlation between coffee consumption and CRC, consistent with our findings. Furthermore, they discovered that decaffeinated coffee may exhibit a protective effect against CRC development. Overall, the present study recommends coffee consumption.

The correlation between BMI and cancer risk has provoked the interest of researchers^48^. Vithayathil et al. conducted an MR study^49^ and revealed that genetically predicted BMI was positively correlated with GC, EC, HCC, and PC among European population but demonstrated no correlation with CRC and BTC. Bull et al. performed an MR study^50^ and showed that high BMI was associated with higher odds of CRC in men, but not in women. However, Suzuki et al. conducted an MR study^51^ and discovered that, for every one-unit increase in genetically predicted BMI in Japanese participants, the ORs for CRC increased, whereas our study did not reveal any positive causal association between BMI and CRC. Thus, our findings suggest that MR analyses on the same topic can yield inconsistent results, which can be attributed to differences in ethnic groups and selection criteria for IVs, highlighting the need for cautious interpretation of MR results.

T2D is believed to be linked to the development of several common cancers, and a series of meta-analyses have been published on this topic^52^. Miao et al.^53^ discovered that individuals with T2D experienced little to no change in the risk of stomach cancer. Other studies^54,55^ demonstrated that T2D was linked to an increased incidence of CRC, HCC, and BTC^56^. However, our study did not reveal any causal associations between genetically predicted T2D and six types of DSCs.

MetS represents a cluster of metabolic disorders including obesity, insulin resistance, high blood pressure, and dyslipidemia^57^. Recent observational studies suggest that MetS plays a role in the etiology and progression of various cancers. The meta-analysis of Zhang et al.^58^ indicated that MetS was associated with a higher risk of EC, consistent with our findings. Mariani et al.^59^ reported that Western women with MetS had an increased risk of GC. Our study revealed a causal association between MetS and GC among Asian participants. Mili et al.^60^ found that MetS is positively associated with the risk of CRC, whereas the present study did not observe the association between genetically predicted MetS and CRC. However, the number of SNPs of genetically predicted MetS was limited in our study; therefore, the results of MR analysis should be interpreted with caution.

When conducting research on cancer, it is crucial to consider the various subtypes that exist. In the case of esophageal cancer, it is important to recognize that the mechanisms and risk factors differ for esophageal squamous cell carcinoma and esophageal adenocarcinoma. Esophageal squamous cell carcinoma is linked to factors such as smoking, alcohol consumption, exposure to environmental pollutants, contamination with mold toxins, nutritional imbalances, and the consumption of hot food and beverages. On the other hand, esophageal adenocarcinoma is often associated with obesity and gastroesophageal reflux. Subgroup analysis is highly significant and valuable. Although the BBJ contains the largest available cancer GWAS data for East Asian populations, it does not include data on specific cancer subtypes. Therefore, conducting MR analysis for cancer subtypes is not feasible. It is recommended that future GWAS data on cancer subtypes be published, encompassing a larger sample of East Asian populations, in order to validate or update our MR results.

Nonetheless, our study has several limitations. First, the sample sizes of DSCs in European studies are much smaller than those in Asian studies. Moreover, East Asia is a region with the highest incidence of malignant tumors in the digestive system. Therefore, we explored only the causal effects of metabolism-related lifestyle and clinical risk factors on Asian participants. Second, BBJ includes data from a hospital-based patient-ascertained cohort, other than a population-based healthy volunteer cohort like UK Biobank. The discrepancy in the cohorts may potentially influence the causal effect estimates. Third, the number of SNPs of some of the genetically predicted risk factors (sweet taste, waist-to-hip ratio, educational level, glycine, and MetS) is limited, impairing the robustness of MR results. The limited availability of instrumental variables in MR analysis can exert diverse influences on the outcomes, notably diminishing statistical power and heightening the instability of results. This instability not only complicates the interpretation of the findings but also poses a potential threat to the reliability of the derived conclusions. Fourth, our study is limited to 19 metabolism-related lifestyle and clinical risk factors with available SNPs summary from large-scale GWAS, although other risk factors for malignant tumors of the digestive system have been reported. In future investigations, these limitations should be resolved by using more availably data from large-scale biobanks in more countries. Fifth, due to the differences in SNPs representing each phenotype, it is not feasible to calculate the comprehensive effects of each category. Sixth, colocalization analysis, which integrates multiple GWAS datasets, can more accurately uncover the associations between SNPs and diseases. However, some IVs were derived from independent studies that did not provide complete GWAS data. This makes it challenging for us to perform colocalization analysis. Finally, we explored the causal effect of only genetically predicted continuous variables showing a one-SD increase. We may lose a possible positive association when the highest category is not compared with the lowest category.

Our comprehensive MR analysis showed that alcohol drinking was a strong indicator of GC and HCC development and demonstrated a causal association between coffee consumption and HCC development, TG and BTC development, uric acid and PC development, and MetS and EC and GC development. The SNP rs671 from the ALDH2 gene is of great value for experimental research on the effect of metabolic risk factors on gastrointestinal cancers. Our study provides a better understanding of metabolism-related lifestyle and clinical risk factors for DSCs.

### Supplementary Information

Supplementary Information.

## Data Availability

The datasets used and/or analyzed during the current study are presented in the manuscript. Summary statistics for GWAS are publicly available.
