# Supplementary material for: The effect of metabolism-related lifestyle and clinical risk factors on digestive system cancers in East Asian populations: a two-sample Mendelian randomization analysis
Source: Sci Rep. 2024 Apr 24;14:9474. doi: 10.1038/s41598-024-60122-6 (PMC11043381; doi:10.1038/s41598-024-60122-6)
Supplement: Supplementary file 1 — Supplementary Information. [file 41598_2024_60122_MOESM1_ESM.docx]

| **Supplementary Table 1** Genetic variants used as instrument variables | | | | | | | | | | | |
| --- | --- | --- | --- | --- | --- | --- | --- | --- | --- | --- | --- |
|  |  |  |  |  |  |  |  |  |  |  |  |
| **Exposure** | **rsID** | **EA** | **OA** | **EAF** | **Beta** | **SE** | **Pval** | **Sample size** | **R2** | **F statistic** | **Study** |
| Ever/never drinkers | rs1260326 | C | T | 0.52 | 0.011 | 0.0015 | 1.50E-13 | 165,084 | 6.04032E-05 | 9.972083408 | Matoba, N. et al study |
|  | rs1229984 | C | T | 0.3 | 0.038 | 0.0021 | 1.60E-72 | 165,084 | 0.00060648 | 100.1796883 | Matoba, N. et al study |
|  | rs3043 | C | G | 0.7 | -0.015 | 0.0016 | 3.30E-19 | 165,084 | 0.0000945 | 15.60172336 | Matoba, N. et al study |
|  | rs8187929 | A | T | 0.05 | 0.031 | 0.0041 | 4.60E-14 | 165,084 | 0.000091295 | 15.07253724 | Matoba, N. et al study |
|  | rs671 | A | G | 0.17 | -1.815 | 0.0121 | 1.0E-4740 | 165,084 | 0.929630295 | 2180842.287 | Matoba, N. et al study |
| Sweet taste | rs671 | A | G | 0.266 | 0.22 | 0.012 | 2.10E-70 | 12,312 | 0.018899619 | 237.1360942 | Kawafune, K. et al study |
|  | rs4552669 | A | G | 0.699 | 0.061 | 0.012 | 5.10E-07 | 12,312 | 0.001565789 | 19.30509471 | Kawafune, K. et al study |
|  | rs13347339 | C | T | 0.274 | -0.064 | 0.013 | 6.30E-07 | 12,312 | 0.001629585 | 20.09293953 | Kawafune, K. et al study |
| Coffee consumption | rs6681426 | A | G | 0.65 | -0.078 | 0.0118 | 1.1×10-10 | 152,634 | 0.00276822 | 423.6918272 | Matoba, N. et al study |
|  | rs1260326 | C | T | 0.52 | 0.096 | 0.0113 | 9.9×10-17 | 152,634 | 0.004600627 | 705.4484361 | Matoba, N. et al study |
|  | rs4410790 | C | T | 0.41 | 0.213 | 0.0119 | 8.1×10-68 | 152,634 | 0.021949522 | 3425.385037 | Matoba, N. et al study |
|  | rs671 | A | G | 0.17 | 0.354 | 0.0131 | 3.8×10-153 | 152,634 | 0.035364175 | 5595.588149 | Matoba, N. et al study |
|  | rs58806801 | A | G | 0.24 | -0.084 | 0.0137 | 2.4×10-9 | 152,634 | 0.002574029 | 393.8930559 | Matoba, N. et al study |
|  | rs5760444 | C | T | 0.56 | -0.073 | 0.0114 | 3.7×10-10 | 152,634 | 0.002626131 | 401.8870655 | Matoba, N. et al study |
| BMI | rs10062657 | A | C | 0.5947 | -0.03827 | 0.003878 | 5.70E-23 | 158284 | 0.000706027 | 111.8303602 | Biobank Japan |
|  | rs1011939 | A | G | 0.3392 | -0.02516 | 0.003874 | 8.33E-11 | 158284 | 0.000283777 | 44.92953632 | Biobank Japan |
|  | rs10208649 | C | T | 0.0132 | -0.1092 | 0.01671 | 6.36E-11 | 158284 | 0.000310655 | 49.1863743 | Biobank Japan |
|  | rs1035491 | G | A | 0.2417 | -0.02617 | 0.004398 | 2.67E-09 | 158284 | 0.000251047 | 39.74620962 | Biobank Japan |
|  | rs10764373 | T | G | 0.625 | 0.02154 | 0.003675 | 4.59E-09 | 158284 | 0.000217487 | 34.43171631 | Biobank Japan |
|  | rs10795945 | C | T | 0.5566 | 0.02157 | 0.00356 | 1.37E-09 | 158284 | 0.000229651 | 36.35803921 | Biobank Japan |
|  | rs10835389 | C | T | 0.5926 | -0.02082 | 0.003603 | 7.54E-09 | 158284 | 0.000209302 | 33.13573096 | Biobank Japan |
|  | rs11030100 | T | G | 0.4075 | -0.03831 | 0.003609 | 2.53E-26 | 158284 | 0.000708713 | 112.2560343 | Biobank Japan |
|  | rs11191021 | G | A | 0.4452 | 0.02076 | 0.003601 | 8.16E-09 | 158284 | 0.0002129 | 33.70546341 | Biobank Japan |
|  | rs11602339 | T | C | 0.3138 | 0.02399 | 0.003907 | 8.24E-10 | 158284 | 0.000247853 | 39.2403912 | Biobank Japan |
|  | rs11642015 | T | C | 0.2058 | 0.07922 | 0.004414 | 5.03E-72 | 158284 | 0.002051516 | 325.3856017 | Biobank Japan |
|  | rs12597682 | A | C | 0.178 | -0.03034 | 0.004649 | 6.75E-11 | 158284 | 0.000269372 | 42.64827799 | Biobank Japan |
|  | rs12617004 | C | G | 0.3918 | 0.02042 | 0.003663 | 2.48E-08 | 158284 | 0.000198725 | 31.46082896 | Biobank Japan |
|  | rs1518170 | C | T | 0.6858 | -0.02136 | 0.003827 | 2.39E-08 | 158284 | 0.000196624 | 31.12813377 | Biobank Japan |
|  | rs1568079 | A | T | 0.3558 | -0.0251 | 0.003708 | 1.30E-11 | 158284 | 0.000288805 | 45.72577515 | Biobank Japan |
|  | rs16937956 | G | A | 0.5639 | -0.02051 | 0.003575 | 9.63E-09 | 158284 | 0.000206895 | 32.7544936 | Biobank Japan |
|  | rs16978956 | G | A | 0.1884 | 0.02649 | 0.004567 | 6.62E-09 | 158284 | 0.000214594 | 33.97360125 | Biobank Japan |
|  | rs1832886 | A | G | 0.7838 | 0.03007 | 0.004567 | 4.57E-11 | 158284 | 0.000306449 | 48.52018401 | Biobank Japan |
|  | rs1907240 | A | G | 0.6975 | -0.0259 | 0.003855 | 1.84E-11 | 158284 | 0.000283073 | 44.81811624 | Biobank Japan |
|  | rs1996023 | G | T | 0.7112 | -0.032 | 0.003937 | 4.36E-16 | 158284 | 0.000420648 | 66.60903502 | Biobank Japan |
|  | rs2206271 | A | T | 0.3501 | 0.03064 | 0.003752 | 3.18E-16 | 158284 | 0.000427215 | 67.6492944 | Biobank Japan |
|  | rs2390669 | C | A | 0.2416 | 0.02549 | 0.004117 | 5.96E-10 | 158284 | 0.000238103 | 37.69639932 | Biobank Japan |
|  | rs2540034 | T | C | 0.3238 | 0.02823 | 0.004261 | 3.47E-11 | 158284 | 0.000348983 | 55.25694619 | Biobank Japan |
|  | rs35560038 | T | A | 0.6113 | -0.05502 | 0.003704 | 6.53E-50 | 158284 | 0.0014386 | 228.0325582 | Biobank Japan |
|  | rs3888190 | A | C | 0.1441 | 0.03043 | 0.005044 | 1.61E-09 | 158284 | 0.000228413 | 36.16193379 | Biobank Japan |
|  | rs3932549 | C | A | 0.6873 | 0.02437 | 0.004348 | 2.08E-08 | 158284 | 0.000255279 | 40.41640575 | Biobank Japan |
|  | rs4357030 | T | C | 0.5296 | 0.02236 | 0.003961 | 1.65E-08 | 158284 | 0.000249109 | 39.43924685 | Biobank Japan |
|  | rs4366055 | C | A | 0.4889 | -0.01983 | 0.003545 | 2.22E-08 | 158284 | 0.000196518 | 31.11130485 | Biobank Japan |
|  | rs4409766 | C | T | 0.2829 | 0.02504 | 0.003944 | 2.17E-10 | 158284 | 0.000254397 | 40.27665 | Biobank Japan |
|  | rs4686392 | G | A | 0.3367 | -0.03289 | 0.003753 | 1.89E-18 | 158284 | 0.000483182 | 76.51600372 | Biobank Japan |
|  | rs4790981 | G | A | 0.7124 | 0.02298 | 0.003979 | 7.68E-09 | 158284 | 0.000216393 | 34.25850086 | Biobank Japan |
|  | rs4811309 | A | G | 0.3631 | -0.02051 | 0.003718 | 3.46E-08 | 158284 | 0.000194562 | 30.80171152 | Biobank Japan |
|  | rs491055 | G | A | 0.6309 | -0.02158 | 0.003723 | 6.78E-09 | 158284 | 0.000216889 | 34.33706591 | Biobank Japan |
|  | rs5015933 | C | T | 0.5401 | -0.0223 | 0.003558 | 3.67E-10 | 158284 | 0.000247046 | 39.11255093 | Biobank Japan |
|  | rs55934576 | C | T | 0.2898 | -0.02424 | 0.004333 | 2.22E-08 | 158284 | 0.000241866 | 38.29224762 | Biobank Japan |
|  | rs60808706 | A | G | 0.3929 | 0.04596 | 0.003669 | 5.34E-36 | 158284 | 0.001007702 | 159.6620447 | Biobank Japan |
|  | rs62116682 | T | C | 0.2461 | -0.02449 | 0.004118 | 2.73E-09 | 158284 | 0.000222553 | 35.23393237 | Biobank Japan |
|  | rs633715 | C | T | 0.2254 | 0.04774 | 0.004249 | 2.73E-29 | 158284 | 0.000795841 | 126.0676117 | Biobank Japan |
|  | rs6567160 | C | T | 0.2162 | 0.051 | 0.004293 | 1.51E-32 | 158284 | 0.000881518 | 139.6515734 | Biobank Japan |
|  | rs6734118 | A | C | 0.5381 | 0.02268 | 0.003552 | 1.71E-10 | 158284 | 0.000255698 | 40.48271602 | Biobank Japan |
|  | rs6881648 | C | A | 0.5609 | -0.02439 | 0.00357 | 8.38E-12 | 158284 | 0.000293024 | 46.39394249 | Biobank Japan |
|  | rs6882046 | G | A | 0.4886 | 0.02422 | 0.003544 | 8.25E-12 | 158284 | 0.000293152 | 46.41424835 | Biobank Japan |
|  | rs6913361 | G | A | 0.8516 | -0.03743 | 0.005305 | 1.72E-12 | 158284 | 0.000354111 | 56.06922438 | Biobank Japan |
|  | rs6947395 | T | A | 0.1975 | 0.0305 | 0.004442 | 6.59E-12 | 158284 | 0.000294878 | 46.68758687 | Biobank Japan |
|  | rs7020996 | T | C | 0.4194 | 0.03484 | 0.003853 | 1.53E-19 | 158284 | 0.000591142 | 93.62246699 | Biobank Japan |
|  | rs713586 | C | T | 0.5109 | 0.02505 | 0.003621 | 4.58E-12 | 158284 | 0.000313602 | 49.65314571 | Biobank Japan |
|  | rs7559954 | T | C | 0.5722 | -0.02052 | 0.003682 | 2.50E-08 | 158284 | 0.000206145 | 32.63581093 | Biobank Japan |
|  | rs75766425 | C | G | 0.1272 | 0.03988 | 0.00552 | 5.02E-13 | 158284 | 0.000353136 | 55.91483726 | Biobank Japan |
|  | rs7692081 | G | A | 0.7982 | -0.02456 | 0.004433 | 3.02E-08 | 158284 | 0.000194321 | 30.76348525 | Biobank Japan |
|  | rs77489951 | T | C | 0.064 | 0.04442 | 0.007976 | 2.56E-08 | 158284 | 0.000236398 | 37.42632067 | Biobank Japan |
|  | rs77636220 | A | G | 0.2041 | 0.02717 | 0.004419 | 7.82E-10 | 158284 | 0.000239834 | 37.97051453 | Biobank Japan |
|  | rs80234489 | C | A | 0.1911 | -0.03183 | 0.00474 | 1.88E-11 | 158284 | 0.000313227 | 49.59368492 | Biobank Japan |
|  | rs9266629 | C | T | 0.1921 | -0.02569 | 0.004498 | 1.12E-08 | 158284 | 0.000204853 | 32.43124957 | Biobank Japan |
|  | rs939584 | T | C | 0.8964 | 0.05406 | 0.005971 | 1.38E-19 | 158284 | 0.000542805 | 85.96289086 | Biobank Japan |
|  | rs9397585 | C | T | 0.6455 | 0.02195 | 0.003713 | 3.39E-09 | 158284 | 0.000220501 | 34.90911455 | Biobank Japan |
|  | rs9425762 | G | C | 0.2204 | 0.02367 | 0.004317 | 4.18E-08 | 158284 | 0.000192535 | 30.48071052 | Biobank Japan |
|  | rs9568867 | A | G | 0.2235 | 0.03141 | 0.004252 | 1.50E-13 | 158284 | 0.00034244 | 54.22070131 | Biobank Japan |
| Waist circumference (adjBMI) | rs3791679 | A | G | 0.21 | 2.87 | 0.38 | 4.86E-14 | 64,454 | 2.73300342 | 57.04224377 | Wen, W. et al study |
|  | rs8030379 | A | G | 0.76 | 2.46 | 0.41 | 1.62E-09 | 50,668 | 2.20762368 | 36 | Wen, W. et al study |
|  | rs3809128 | C | T | 0.8 | 3.69 | 0.63 | 3.74E-09 | 30368 | 4.357152 | 34.30612245 | Wen, W. et al study |
|  | rs2057291 | G | A | 0.73 | 2.52 | 0.46 | 4.02E-08 | 38613 | 2.50332768 | 30.01134216 | Wen, W. et al study |
| Waist-hip ratio (adjBMI) | rs1982963 | A | G | 0.81 | 4.82 | 0.62 | 1.07E-14 | 56,208 | 7.15093272 | 60.43808533 | Wen, W. et al study |
|  | rs5020946 | T | G | 0.41 | 3.15 | 0.52 | 1.30E-09 | 49,519 | 4.8005055 | 36.69563609 | Wen, W. et al study |
| Education | rs280536 | A | G | 0.8933 | -0.2489 | 0.0472 | 1.33E-07 | 1,111 | 0.011809773 | 13.25355955 | pan-ancestry genetic analysis of the UK Biobank |
|  | rs77827642 | T | C | 0.98058 | -0.5911 | 0.1151 | 2.81E-07 | 1,111 | 0.013307123 | 14.95662911 | pan-ancestry genetic analysis of the UK Biobank |
|  | rs2688072 | A | T | 0.0503 | 0.3699 | 0.07148 | 2.27E-07 | 1,111 | 0.013072332 | 14.68923921 | pan-ancestry genetic analysis of the UK Biobank |
| HDL-C | rs3764261 | A | C | 0.1693 | 0.262 | 0.0141 | 4.53E-77 | 34,421 | 0.019307842 | 677.6404043 | Asian Genetic Epidemiology Network |
|  | rs662799 | A | G | 0.7251 | 0.1851 | 0.01 | 1.72E-76 | 34,421 | 0.013658892 | 476.6357274 | Asian Genetic Epidemiology Network |
|  | rs1800588 | T | C | 0.4016 | 0.137 | 0.0099 | 1.50E-43 | 34,421 | 0.009021036 | 313.321525 | Asian Genetic Epidemiology Network |
|  | rs1011685 | T | C | 0.1047 | 0.1664 | 0.0131 | 5.74E-37 | 34,421 | 0.00519101 | 179.6017041 | Asian Genetic Epidemiology Network |
|  | rs16940212 | T | G | 0.2969 | 0.0992 | 0.0084 | 3.49E-32 | 34,421 | 0.004108475 | 141.9929713 | Asian Genetic Epidemiology Network |
|  | rs1883025 | T | C | 0.2462 | -0.0983 | 0.0091 | 3.36E-27 | 34,421 | 0.003586586 | 123.8910392 | Asian Genetic Epidemiology Network |
|  | rs3786247 | T | G | 0.5993 | -0.0957 | 0.0102 | 6.45E-21 | 34,421 | 0.004398631 | 152.065346 | Asian Genetic Epidemiology Network |
|  | rs4420638 | A | G | 0.8834 | 0.1129 | 0.0132 | 1.20E-17 | 34,421 | 0.002625874 | 90.61789624 | Asian Genetic Epidemiology Network |
|  | rs1883023 | T | C | 0.3813 | -0.061 | 0.008 | 2.44E-14 | 34,421 | 0.001755645 | 60.53380482 | Asian Genetic Epidemiology Network |
|  | rs11066280 | A | T | 0.2148 | -0.077 | 0.0108 | 1.01E-12 | 34,421 | 0.001999982 | 68.97531825 | Asian Genetic Epidemiology Network |
|  | rs3760782 | T | C | 0.2531 | -0.0676 | 0.0107 | 2.65E-10 | 34,421 | 0.001727738 | 59.56995015 | Asian Genetic Epidemiology Network |
|  | rs1109166 | T | C | 0.8717 | -0.0749 | 0.0118 | 2.19E-10 | 34,421 | 0.001254837 | 43.24450126 | Asian Genetic Epidemiology Network |
|  | rs6124760 | C | G | 0.9368 | 0.1043 | 0.0168 | 5.35E-10 | 34,421 | 0.001288139 | 44.39362542 | Asian Genetic Epidemiology Network |
|  | rs948937 | A | T | 0.2854 | -0.0525 | 0.0086 | 1.03E-09 | 34,421 | 0.001124257 | 38.73935314 | Asian Genetic Epidemiology Network |
|  | rs445925 | A | G | 0.0937 | 0.1197 | 0.0207 | 7.36E-09 | 34,421 | 0.002433492 | 83.96267291 | Asian Genetic Epidemiology Network |
| LDL-C | rs445925 | A | G | 0.0937 | -0.5219 | 0.0214 | 2.30E-131 | 34,421 | 0.046261122 | 1669.494228 | Asian Genetic Epidemiology Network |
|  | rs599839 | A | G | 0.9307 | 0.1877 | 0.0162 | 4.83E-31 | 34,421 | 0.004544661 | 157.1368191 | Asian Genetic Epidemiology Network |
|  | rs10119 | A | G | 0.1435 | 0.2063 | 0.019 | 1.83E-27 | 34,421 | 0.010461831 | 363.8927624 | Asian Genetic Epidemiology Network |
|  | rs12916 | T | C | 0.4747 | -0.0793 | 0.0082 | 4.01E-22 | 34,421 | 0.003136195 | 108.2842825 | Asian Genetic Epidemiology Network |
|  | rs2738464 | C | G | 0.727 | 0.0957 | 0.012 | 1.52E-15 | 34,421 | 0.003635389 | 125.5830087 | Asian Genetic Epidemiology Network |
|  | rs8051431 | C | G | 0.7098 | 0.0753 | 0.0093 | 5.64E-16 | 34,421 | 0.002335895 | 80.58742009 | Asian Genetic Epidemiology Network |
|  | rs10172650 | A | G | 0.132 | -0.0945 | 0.012 | 3.41E-15 | 34,421 | 0.002046385 | 70.57894487 | Asian Genetic Epidemiology Network |
|  | rs2980869 | T | C | 0.5606 | -0.0503 | 0.0084 | 2.12E-09 | 34,421 | 0.001246462 | 42.95552481 | Asian Genetic Epidemiology Network |
|  | rs174533 | A | G | 0.3839 | -0.0602 | 0.0104 | 7.10E-09 | 34,421 | 0.001714322 | 59.10656307 | Asian Genetic Epidemiology Network |
|  | rs1799955 | A | G | 0.6284 | 0.0517 | 0.0087 | 2.81E-09 | 34,421 | 0.001248311 | 43.01933427 | Asian Genetic Epidemiology Network |
|  | rs505151 | A | G | 0.9442 | -0.104 | 0.0184 | 1.58E-08 | 34,421 | 0.001139711 | 39.27248389 | Asian Genetic Epidemiology Network |
|  | rs12117661 | C | G | 0.8569 | 0.0764 | 0.0141 | 6.01E-08 | 34,421 | 0.001431484 | 49.34087748 | Asian Genetic Epidemiology Network |
| TC-C | rs7254892 | A | G | 0.0723 | -0.3981 | 0.0264 | 2.21E-51 | 34,421 | 0.02125985 | 747.6374522 | Asian Genetic Epidemiology Network |
|  | rs599839 | A | G | 0.9307 | 0.1684 | 0.0161 | 1.32E-25 | 34,421 | 0.003658113 | 126.3708708 | Asian Genetic Epidemiology Network |
|  | rs12916 | T | C | 0.4747 | -0.0794 | 0.0081 | 1.10E-22 | 34,421 | 0.003144109 | 108.558417 | Asian Genetic Epidemiology Network |
|  | rs2980869 | T | C | 0.5606 | -0.0705 | 0.0084 | 4.74E-17 | 34,421 | 0.00244862 | 84.48592244 | Asian Genetic Epidemiology Network |
|  | rs9958734 | T | C | 0.6105 | -0.0919 | 0.0111 | 1.24E-16 | 34,421 | 0.004016559 | 138.8034558 | Asian Genetic Epidemiology Network |
|  | rs10172650 | A | G | 0.132 | -0.0973 | 0.012 | 5.13E-16 | 34,421 | 0.002169448 | 74.83259124 | Asian Genetic Epidemiology Network |
|  | rs1865063 | T | C | 0.2543 | -0.0775 | 0.0095 | 3.41E-16 | 34,421 | 0.002277949 | 78.58371957 | Asian Genetic Epidemiology Network |
|  | rs1883025 | T | C | 0.2482 | -0.0735 | 0.0096 | 1.91E-14 | 34,421 | 0.002016085 | 69.53180106 | Asian Genetic Epidemiology Network |
|  | rs1122531 | T | C | 0.7216 | 0.0684 | 0.0092 | 1.05E-13 | 34,421 | 0.001879784 | 64.82213799 | Asian Genetic Epidemiology Network |
|  | rs2070895 | A | G | 0.4054 | 0.0757 | 0.0108 | 2.40E-12 | 34,421 | 0.002762679 | 95.3520707 | Asian Genetic Epidemiology Network |
|  | rs7776054 | A | G | 0.7154 | 0.0598 | 0.0093 | 1.28E-10 | 34,421 | 0.001456184 | 50.19348114 | Asian Genetic Epidemiology Network |
|  | rs10402592 | T | C | 0.1481 | -0.1005 | 0.0159 | 2.60E-10 | 34,421 | 0.002548624 | 87.94523435 | Asian Genetic Epidemiology Network |
|  | rs780092 | A | G | 0.6658 | 0.0526 | 0.0087 | 1.49E-09 | 34,421 | 0.001231266 | 42.43117222 | Asian Genetic Epidemiology Network |
|  | rs174533 | A | G | 0.3833 | -0.0549 | 0.0103 | 9.82E-08 | 34,421 | 0.00142491 | 49.11396229 | Asian Genetic Epidemiology Network |
|  | rs1800774 | T | C | 0.1217 | -0.0688 | 0.0125 | 3.71E-08 | 34,421 | 0.001011906 | 34.8640848 | Asian Genetic Epidemiology Network |
|  | rs17122278 | A | G | 0.5268 | -0.0469 | 0.0088 | 9.85E-08 | 34,421 | 0.001096645 | 37.78687352 | Asian Genetic Epidemiology Network |
| TG-C | rs662799 | A | G | 0.7254 | -0.2825 | 0.0116 | 5.33E-131 | 34421 | 0.031793986 | 1130.252449 | Asian Genetic Epidemiology Network |
|  | rs780094 | T | C | 0.5155 | 0.1047 | 0.0087 | 2.34E-33 | 34421 | 0.005475778 | 189.5084996 | Asian Genetic Epidemiology Network |
|  | rs17482753 | T | G | 0.1065 | -0.154 | 0.0144 | 1.08E-26 | 34421 | 0.004513522 | 156.0552864 | Asian Genetic Epidemiology Network |
|  | rs1051921 | A | G | 0.1069 | -0.1202 | 0.0142 | 2.57E-17 | 34421 | 0.002758778 | 95.21705646 | Asian Genetic Epidemiology Network |
|  | rs995000 | T | C | 0.1876 | -0.0801 | 0.0109 | 2E-13 | 34421 | 0.00195568 | 67.44444691 | Asian Genetic Epidemiology Network |
|  | rs1800588 | T | C | 0.3963 | 0.0839 | 0.0115 | 2.97E-13 | 34421 | 0.00336821 | 116.3222189 | Asian Genetic Epidemiology Network |
|  | rs2954018 | T | C | 0.706 | -0.0702 | 0.0094 | 8.14E-14 | 34421 | 0.002045767 | 70.55761223 | Asian Genetic Epidemiology Network |
|  | rs157582 | T | C | 0.1941 | 0.1073 | 0.0168 | 1.69E-10 | 34421 | 0.003601937 | 124.4232395 | Asian Genetic Epidemiology Network |
|  | rs7165077 | T | C | 0.3635 | -0.0601 | 0.0109 | 3.51E-08 | 34421 | 0.001671405 | 57.62441116 | Asian Genetic Epidemiology Network |
| Glycine | rs1047891 | A | C | 0.174 | 0.455 | 0.051 | 9.67E-19 | 1,324 | 0.059508922 | 83.64863528 | Chang, X. et al study |
| Uric acid | rs11202346 | T | G | 0.2224 | 0.03489 | 0.00528 | 3.89E-11 | 109029 | 0.00042104 | 45.92402294 | Biobank Japan |
|  | rs11231455 | C | G | 0.0595 | -0.2186 | 0.008996 | 1.82E-130 | 109029 | 0.005348181 | 586.231374 | Biobank Japan |
|  | rs1165196 | A | G | 0.8407 | 0.05158 | 0.0057 | 1.43E-19 | 109029 | 0.000712606 | 77.7487021 | Biobank Japan |
|  | rs1260326 | C | T | 0.4409 | -0.03472 | 0.00424 | 2.65E-16 | 109029 | 0.000594318 | 64.83526164 | Biobank Japan |
|  | rs1549287 | G | A | 0.8452 | -0.03315 | 0.005792 | 1.05E-08 | 109029 | 0.000287559 | 31.36075224 | Biobank Japan |
|  | rs16856823 | T | A | 0.1929 | 0.04066 | 0.005494 | 1.35E-13 | 109029 | 0.000514783 | 56.15416922 | Biobank Japan |
|  | rs1805100 | A | G | 0.432 | 0.03729 | 0.004247 | 1.63E-18 | 109029 | 0.000682412 | 74.45217271 | Biobank Japan |
|  | rs1886603 | A | G | 0.3744 | 0.02607 | 0.004341 | 1.91E-09 | 109029 | 0.000318379 | 34.72298019 | Biobank Japan |
|  | rs4077450 | T | G | 0.5862 | -0.03561 | 0.004707 | 3.86E-14 | 109029 | 0.000615191 | 67.11375869 | Biobank Japan |
|  | rs4148155 | G | A | 0.2955 | 0.1136 | 0.00464 | 2.00E-132 | 109029 | 0.005373103 | 588.9779061 | Biobank Japan |
|  | rs4724828 | T | C | 0.4123 | 0.02793 | 0.005015 | 2.57E-08 | 109029 | 0.000378043 | 41.23245253 | Biobank Japan |
|  | rs4766566 | T | C | 0.6775 | 0.05443 | 0.00446 | 2.97E-34 | 109029 | 0.00129463 | 141.3326037 | Biobank Japan |
|  | rs477138 | G | C | 0.1609 | -0.2061 | 0.005818 | 1.00E-200 | 109029 | 0.011469797 | 1265.0272 | Biobank Japan |
|  | rs59350108 | C | G | 0.3729 | 0.03203 | 0.00435 | 1.81E-13 | 109029 | 0.000479814 | 52.33781044 | Biobank Japan |
|  | rs6026578 | G | C | 0.7227 | -0.02659 | 0.004724 | 1.83E-08 | 109029 | 0.000283384 | 30.90521336 | Biobank Japan |
|  | rs6445559 | G | A | 0.4376 | -0.02594 | 0.004399 | 3.67E-09 | 109029 | 0.000331202 | 36.12189198 | Biobank Japan |
|  | rs6460047 | C | T | 0.106 | -0.04096 | 0.006978 | 4.38E-09 | 109029 | 0.000317975 | 34.67891128 | Biobank Japan |
|  | rs647304 | C | G | 0.3316 | -0.07827 | 0.004489 | 4.34E-68 | 109029 | 0.002715636 | 296.8839221 | Biobank Japan |
|  | rs73575095 | C | T | 0.2808 | -0.03388 | 0.004709 | 6.26E-13 | 109029 | 0.000463622 | 50.57070851 | Biobank Japan |
|  | rs7570707 | T | C | 0.3729 | 0.02428 | 0.00437 | 2.74E-08 | 109029 | 0.000275713 | 30.06840309 | Biobank Japan |
|  | rs7679724 | T | G | 0.586 | 0.1239 | 0.004284 | 5.56E-184 | 109029 | 0.00744853 | 818.1851461 | Biobank Japan |
|  | rs7835379 | A | G | 0.7546 | 0.0303 | 0.004928 | 7.78E-10 | 109029 | 0.000340022 | 37.08415339 | Biobank Japan |
|  | rs9394948 | C | A | 0.6594 | -0.03079 | 0.004536 | 1.13E-11 | 109029 | 0.000425837 | 46.44746326 | Biobank Japan |
|  | rs9416703 | C | A | 0.4751 | 0.03615 | 0.004289 | 3.53E-17 | 109029 | 0.000651791 | 71.1091399 | Biobank Japan |
|  | rs963837 | C | T | 0.3432 | -0.02785 | 0.004867 | 1.05E-08 | 109029 | 0.000349672 | 38.13702112 | Biobank Japan |
|  | rs9895661 | T | C | 0.4776 | 0.04506 | 0.004821 | 9.03E-21 | 109029 | 0.001013164 | 110.5742885 | Biobank Japan |
| Creatinine | rs10459012 | A | C | 0.3255 | 0.02215 | 0.003885 | 1.19E-08 | 142097 | 0.000215432 | 30.61841965 | Biobank Japan |
|  | rs10518732 | C | G | 0.3923 | -0.0452 | 0.003708 | 3.52E-34 | 142097 | 0.000974124 | 138.5531708 | Biobank Japan |
|  | rs10794486 | A | G | 0.4754 | -0.02708 | 0.003716 | 3.16E-13 | 142097 | 0.000365776 | 51.99390773 | Biobank Japan |
|  | rs10857147 | T | A | 0.3014 | -0.03533 | 0.004281 | 1.55E-16 | 142097 | 0.000525641 | 74.73021627 | Biobank Japan |
|  | rs11123169 | T | C | 0.7321 | -0.03103 | 0.004114 | 4.61E-14 | 142097 | 0.000377691 | 53.68828408 | Biobank Japan |
|  | rs11742501 | C | T | 0.0944 | -0.03778 | 0.00624 | 1.41E-09 | 142097 | 0.000244041 | 34.68543183 | Biobank Japan |
|  | rs118082206 | T | C | 0.0519 | 0.05795 | 0.00936 | 5.97E-10 | 142097 | 0.00033049 | 46.97650805 | Biobank Japan |
|  | rs1275609 | A | G | 0.5936 | -0.02821 | 0.003987 | 1.49E-12 | 142097 | 0.000383958 | 54.5794731 | Biobank Japan |
|  | rs12916630 | G | A | 0.2028 | -0.0266 | 0.004514 | 3.80E-09 | 142097 | 0.000228786 | 32.51671598 | Biobank Japan |
|  | rs12935539 | C | T | 0.3153 | 0.02436 | 0.003974 | 8.80E-10 | 142097 | 0.000256218 | 36.41656233 | Biobank Japan |
|  | rs142516820 | G | A | 0.0222 | 0.09646 | 0.01305 | 1.45E-13 | 142097 | 0.00040395 | 57.42245859 | Biobank Japan |
|  | rs1533988 | T | A | 0.3328 | 0.0572 | 0.004462 | 1.28E-37 | 142097 | 0.001452986 | 206.7624585 | Biobank Japan |
|  | rs16856823 | T | A | 0.1929 | -0.04923 | 0.004744 | 3.14E-25 | 142097 | 0.000754656 | 107.313882 | Biobank Japan |
|  | rs16942751 | A | C | 0.2962 | 0.02425 | 0.004075 | 2.67E-09 | 142097 | 0.000245182 | 34.8476173 | Biobank Japan |
|  | rs16972495 | C | A | 0.2514 | -0.02353 | 0.004206 | 2.21E-08 | 142097 | 0.000208396 | 29.61817242 | Biobank Japan |
|  | rs17001974 | T | C | 0.2991 | -0.02934 | 0.003993 | 2.01E-13 | 142097 | 0.00036093 | 51.30483109 | Biobank Japan |
|  | rs1705694 | G | A | 0.8364 | -0.03389 | 0.004966 | 8.83E-12 | 142097 | 0.000314319 | 44.67718295 | Biobank Japan |
|  | rs241812 | G | A | 0.5705 | -0.02155 | 0.003686 | 5.02E-09 | 142097 | 0.000227585 | 32.34603172 | Biobank Japan |
|  | rs2511162 | G | A | 0.4402 | -0.02077 | 0.00368 | 1.66E-08 | 142097 | 0.000212611 | 30.21739788 | Biobank Japan |
|  | rs2736609 | T | C | 0.3804 | -0.02174 | 0.003787 | 9.43E-09 | 142097 | 0.000222793 | 31.664786 | Biobank Japan |
|  | rs2781656 | T | C | 0.3202 | 0.02604 | 0.003918 | 3.01E-11 | 142097 | 0.000295199 | 41.95864567 | Biobank Japan |
|  | rs307558 | A | G | 0.7437 | -0.02628 | 0.004258 | 6.75E-10 | 142097 | 0.000263286 | 37.42141972 | Biobank Japan |
|  | rs316020 | G | A | 0.9485 | 0.0553 | 0.008178 | 1.36E-11 | 142097 | 0.000298762 | 42.46522102 | Biobank Japan |
|  | rs34720381 | T | C | 0.0693 | 0.0417 | 0.007267 | 9.56E-09 | 142097 | 0.000224308 | 31.88021761 | Biobank Japan |
|  | rs3734861 | A | G | 0.0887 | -0.03594 | 0.006378 | 1.75E-08 | 142097 | 0.00020882 | 29.67840973 | Biobank Japan |
|  | rs3782787 | G | C | 0.2567 | 0.02715 | 0.004162 | 6.88E-11 | 142097 | 0.000281293 | 39.98164353 | Biobank Japan |
|  | rs3812036 | T | C | 0.2532 | 0.03549 | 0.004248 | 6.57E-17 | 142097 | 0.000476332 | 67.71668961 | Biobank Japan |
|  | rs4399402 | G | A | 0.8176 | -0.02775 | 0.004774 | 6.15E-09 | 142097 | 0.000229679 | 32.64376506 | Biobank Japan |
|  | rs4665987 | A | G | 0.5759 | -0.03616 | 0.003805 | 2.03E-21 | 142097 | 0.000638708 | 90.81518302 | Biobank Japan |
|  | rs4690095 | T | C | 0.4697 | 0.02386 | 0.003684 | 9.38E-11 | 142097 | 0.000283604 | 40.31020839 | Biobank Japan |
|  | rs4715491 | G | A | 0.2291 | 0.03202 | 0.004437 | 5.33E-13 | 142097 | 0.000362156 | 51.47921229 | Biobank Japan |
|  | rs4859682 | A | C | 0.2167 | 0.04946 | 0.004403 | 2.80E-29 | 142097 | 0.000830473 | 118.1040725 | Biobank Japan |
|  | rs549752 | G | A | 0.6824 | -0.04186 | 0.004005 | 1.44E-25 | 142097 | 0.000759535 | 108.0082027 | Biobank Japan |
|  | rs6026578 | G | C | 0.7227 | -0.02583 | 0.004086 | 2.59E-10 | 142097 | 0.000267416 | 38.00858321 | Biobank Japan |
|  | rs67332916 | T | C | 0.398 | -0.02647 | 0.003918 | 1.42E-11 | 142097 | 0.000335751 | 47.72457585 | Biobank Japan |
|  | rs6851943 | T | G | 0.4594 | 0.02146 | 0.003695 | 6.33E-09 | 142097 | 0.000228748 | 32.51132089 | Biobank Japan |
|  | rs6907843 | T | C | 0.084 | 0.04016 | 0.006813 | 3.76E-09 | 142097 | 0.000248195 | 35.27595362 | Biobank Japan |
|  | rs7123489 | A | C | 0.1593 | 0.03034 | 0.005054 | 1.94E-09 | 142097 | 0.000246557 | 35.04320828 | Biobank Japan |
|  | rs715 | C | T | 0.1631 | 0.03693 | 0.005244 | 1.89E-12 | 142097 | 0.00037232 | 52.92448769 | Biobank Japan |
|  | rs716877 | G | C | 0.1492 | 0.03724 | 0.005142 | 4.41E-13 | 142097 | 0.000352083 | 50.04692238 | Biobank Japan |
|  | rs7177266 | T | C | 0.5049 | 0.02833 | 0.004195 | 1.45E-11 | 142097 | 0.000401256 | 57.03934586 | Biobank Japan |
|  | rs7212715 | T | C | 0.7474 | 0.03478 | 0.004864 | 8.65E-13 | 142097 | 0.000456747 | 64.93110519 | Biobank Japan |
|  | rs7247977 | C | T | 0.6591 | -0.02202 | 0.003879 | 1.37E-08 | 142097 | 0.000217893 | 30.96822931 | Biobank Japan |
|  | rs7475348 | T | C | 0.315 | -0.03327 | 0.004025 | 1.39E-16 | 142097 | 0.00047768 | 67.90832559 | Biobank Japan |
|  | rs75174967 | A | G | 0.1516 | 0.02849 | 0.005131 | 2.82E-08 | 142097 | 0.000208792 | 29.67455661 | Biobank Japan |
|  | rs75530000 | T | C | 0.2399 | 0.03013 | 0.004799 | 3.42E-10 | 142097 | 0.000331077 | 47.05999153 | Biobank Japan |
|  | rs75834729 | T | C | 0.29 | -0.02413 | 0.004 | 1.61E-09 | 142097 | 0.000239773 | 34.07877124 | Biobank Japan |
|  | rs7714709 | A | G | 0.7292 | -0.03079 | 0.004365 | 1.74E-12 | 142097 | 0.000374408 | 53.22137903 | Biobank Japan |
|  | rs848302 | A | T | 0.6897 | -0.02697 | 0.003964 | 1.02E-11 | 142097 | 0.000311339 | 44.25353014 | Biobank Japan |
|  | rs881858 | A | G | 0.8792 | 0.03291 | 0.00559 | 3.93E-09 | 142097 | 0.00023006 | 32.69784234 | Biobank Japan |
|  | rs898696 | G | T | 0.4785 | -0.02056 | 0.003688 | 2.48E-08 | 142097 | 0.000210966 | 29.98353946 | Biobank Japan |
|  | rs9272117 | T | C | 0.3459 | 0.02839 | 0.004487 | 2.50E-10 | 142097 | 0.000364717 | 51.84330877 | Biobank Japan |
|  | rs963837 | C | T | 0.3432 | -0.03989 | 0.004218 | 3.17E-21 | 142097 | 0.000717362 | 102.0067583 | Biobank Japan |
|  | rs9887774 | C | A | 0.3131 | -0.02645 | 0.003973 | 2.79E-11 | 142097 | 0.000300925 | 42.7727761 | Biobank Japan |
|  | rs9895661 | T | C | 0.4776 | -0.04348 | 0.004178 | 2.31E-25 | 142097 | 0.000943358 | 134.1730332 | Biobank Japan |
| T2D | rs115001946 | A | G | 0.6073 | 0.0533 | 0.0081 | 4.70E-11 | 443,540 | 0.001355029 | 601.8223478 | Asian Genetic Epidemiology Network |
|  | rs118185025 | A | G | 0.4248 | -0.1222 | 0.0081 | 1.99E-51 | 443,540 | 0.007297528 | 3260.524918 | Asian Genetic Epidemiology Network |
|  | rs1196593 | T | C | 0.1712 | -0.1014 | 0.0115 | 1.17E-18 | 443,540 | 0.002917826 | 1297.953968 | Asian Genetic Epidemiology Network |
|  | rs1207881 | T | C | 0.5332 | -0.0613 | 0.0081 | 3.79E-14 | 443,540 | 0.001870561 | 831.2198422 | Asian Genetic Epidemiology Network |
|  | rs12199742 | T | C | 0.4845 | -0.0572 | 0.0091 | 3.26E-10 | 443,540 | 0.001634348 | 726.0820611 | Asian Genetic Epidemiology Network |
|  | rs1220583 | A | C | 0.7272 | -0.0559 | 0.009 | 5.26E-10 | 443,540 | 0.001239801 | 550.5812964 | Asian Genetic Epidemiology Network |
|  | rs12245132 | T | C | 0.6207 | 0.0824 | 0.0083 | 3.15E-23 | 443,540 | 0.003197047 | 1422.559778 | Asian Genetic Epidemiology Network |
|  | rs12246765 | T | C | 0.379 | -0.0825 | 0.0083 | 2.80E-23 | 443,540 | 0.003203824 | 1425.585186 | Asian Genetic Epidemiology Network |
|  | rs12267476 | A | G | 0.7816 | -0.0703 | 0.0097 | 4.25E-13 | 443,540 | 0.001687244 | 749.6215167 | Asian Genetic Epidemiology Network |
|  | rs12281632 | A | G | 0.5784 | 0.0901 | 0.0081 | 9.65E-29 | 443,540 | 0.003959209 | 1763.040032 | Asian Genetic Epidemiology Network |
|  | rs12288444 | C | G | 0.2122 | 0.0769 | 0.0097 | 2.23E-15 | 443,540 | 0.00197717 | 878.6873804 | Asian Genetic Epidemiology Network |
|  | rs12289262 | T | C | 0.8522 | 0.0925 | 0.0166 | 2.51E-08 | 443,540 | 0.002155408 | 958.0702415 | Asian Genetic Epidemiology Network |
|  | rs12289775 | A | G | 0.285 | 0.0751 | 0.0088 | 1.41E-17 | 443,540 | 0.002298586 | 1021.859102 | Asian Genetic Epidemiology Network |
|  | rs12307894 | T | C | 0.4987 | 0.1211 | 0.008 | 9.16E-52 | 443,540 | 0.007332555 | 3276.290553 | Asian Genetic Epidemiology Network |
|  | rs12309269 | T | C | 0.5677 | 0.1025 | 0.0082 | 7.47E-36 | 443,540 | 0.005156819 | 2299.101064 | Asian Genetic Epidemiology Network |
|  | rs12310737 | T | C | 0.6578 | -0.0517 | 0.0084 | 7.52E-10 | 443,540 | 0.001203331 | 534.3658609 | Asian Genetic Epidemiology Network |
|  | rs12312203 | A | G | 0.6878 | 0.0837 | 0.0086 | 2.19E-22 | 443,540 | 0.00300868 | 1338.490948 | Asian Genetic Epidemiology Network |
|  | rs12328738 | T | C | 0.33 | -0.0732 | 0.009 | 4.18E-16 | 443,540 | 0.002369414 | 1053.421016 | Asian Genetic Epidemiology Network |
|  | rs12332447 | T | C | 0.763 | -0.0811 | 0.0097 | 6.23E-17 | 443,540 | 0.002378727 | 1057.571456 | Asian Genetic Epidemiology Network |
|  | rs1243617 | T | C | 0.3064 | 0.0606 | 0.0087 | 3.27E-12 | 443,540 | 0.001560893 | 693.3976094 | Asian Genetic Epidemiology Network |
|  | rs1252028 | A | G | 0.3064 | 0.0608 | 0.0088 | 4.88E-12 | 443,540 | 0.001571213 | 697.9892584 | Asian Genetic Epidemiology Network |
|  | rs1257311 | T | C | 0.7304 | -0.0628 | 0.0092 | 8.73E-12 | 443,540 | 0.001553209 | 689.978954 | Asian Genetic Epidemiology Network |
|  | rs1286227 | A | T | 0.6082 | 0.0891 | 0.0084 | 2.76E-26 | 443,540 | 0.003783522 | 1684.50911 | Asian Genetic Epidemiology Network |
|  | rs1288126 | T | C | 0.3924 | -0.089 | 0.0084 | 3.14E-26 | 443,540 | 0.003777085 | 1681.632452 | Asian Genetic Epidemiology Network |
|  | rs1288491 | T | C | 0.6086 | 0.0893 | 0.0084 | 2.14E-26 | 443,540 | 0.003799143 | 1691.490667 | Asian Genetic Epidemiology Network |
|  | rs1289447 | A | G | 0.3887 | -0.0895 | 0.0084 | 1.66E-26 | 443,540 | 0.003806668 | 1694.853662 | Asian Genetic Epidemiology Network |
|  | rs1289673 | T | C | 0.6093 | 0.0892 | 0.0084 | 2.43E-26 | 443,540 | 0.003788212 | 1686.605263 | Asian Genetic Epidemiology Network |
|  | rs1290788 | T | C | 0.388 | -0.0881 | 0.0084 | 9.80E-26 | 443,540 | 0.003686082 | 1640.966053 | Asian Genetic Epidemiology Network |
|  | rs1291058 | T | C | 0.3024 | -0.0784 | 0.0093 | 3.45E-17 | 443,540 | 0.002593286 | 1153.211398 | Asian Genetic Epidemiology Network |
|  | rs1294228 | T | G | 0.6085 | 0.0888 | 0.0084 | 4.04E-26 | 443,540 | 0.003757061 | 1672.683812 | Asian Genetic Epidemiology Network |
|  | rs1297380 | T | C | 0.3927 | -0.088 | 0.0084 | 1.11E-25 | 443,540 | 0.003693682 | 1644.362151 | Asian Genetic Epidemiology Network |
|  | rs1300258 | A | G | 0.6069 | 0.0884 | 0.0083 | 1.73E-26 | 443,540 | 0.003728677 | 1659.999323 | Asian Genetic Epidemiology Network |
|  | rs1313566 | T | C | 0.5635 | 0.0825 | 0.0095 | 3.81E-18 | 443,540 | 0.003348236 | 1490.058967 | Asian Genetic Epidemiology Network |
|  | rs1317192 | T | C | 0.5266 | -0.0696 | 0.0082 | 2.11E-17 | 443,540 | 0.002415225 | 1073.837595 | Asian Genetic Epidemiology Network |
|  | rs1323789 | A | G | 0.5707 | 0.084 | 0.0082 | 1.26E-24 | 443,540 | 0.003457461 | 1538.83594 | Asian Genetic Epidemiology Network |
|  | rs1324793 | A | G | 0.4295 | -0.0839 | 0.0082 | 1.43E-24 | 443,540 | 0.003449632 | 1535.339114 | Asian Genetic Epidemiology Network |
|  | rs1324993 | T | C | 0.3948 | -0.0895 | 0.0084 | 1.66E-26 | 443,540 | 0.003827825 | 1704.309861 | Asian Genetic Epidemiology Network |
|  | rs1330759 | T | C | 0.4816 | 0.0659 | 0.0081 | 4.09E-16 | 443,540 | 0.002168464 | 963.8865151 | Asian Genetic Epidemiology Network |
|  | rs1333987 | T | C | 0.7121 | -0.0632 | 0.009 | 2.18E-12 | 443,540 | 0.001637747 | 727.5946286 | Asian Genetic Epidemiology Network |
|  | rs1342521 | A | G | 0.5048 | -0.0686 | 0.0082 | 5.97E-17 | 443,540 | 0.002352763 | 1046.000854 | Asian Genetic Epidemiology Network |
|  | rs1343405 | T | C | 0.4953 | 0.0686 | 0.0082 | 5.97E-17 | 443,540 | 0.002352772 | 1046.004839 | Asian Genetic Epidemiology Network |
|  | rs1345956 | T | C | 0.3109 | 0.0614 | 0.0089 | 5.24E-12 | 443,540 | 0.001615361 | 717.6334167 | Asian Genetic Epidemiology Network |
|  | rs1346796 | A | G | 0.6014 | 0.0887 | 0.0084 | 4.59E-26 | 443,540 | 0.003772054 | 1679.384216 | Asian Genetic Epidemiology Network |
|  | rs1351509 | T | C | 0.3728 | 0.0667 | 0.0085 | 4.26E-15 | 443,540 | 0.00208048 | 924.6959021 | Asian Genetic Epidemiology Network |
|  | rs1357051 | A | G | 0.4083 | -0.0863 | 0.0084 | 9.25E-25 | 443,540 | 0.003598591 | 1601.876526 | Asian Genetic Epidemiology Network |
|  | rs1359604 | A | G | 0.4068 | -0.0859 | 0.0084 | 1.51E-24 | 443,540 | 0.003561217 | 1585.180134 | Asian Genetic Epidemiology Network |
|  | rs1365858 | T | C | 0.2179 | 0.0607 | 0.0102 | 2.67E-09 | 443,540 | 0.001255819 | 557.7036227 | Asian Genetic Epidemiology Network |
|  | rs1368342 | A | G | 0.4171 | -0.086 | 0.0085 | 4.61E-24 | 443,540 | 0.003596343 | 1600.872206 | Asian Genetic Epidemiology Network |
|  | rs1373551 | T | C | 0.6931 | -0.056 | 0.0102 | 4.01E-08 | 443,540 | 0.001334132 | 592.5287995 | Asian Genetic Epidemiology Network |
|  | rs1380699 | A | G | 0.0741 | -0.1229 | 0.0222 | 3.09E-08 | 443,540 | 0.002072603 | 921.1872988 | Asian Genetic Epidemiology Network |
|  | rs1381798 | A | G | 0.3071 | 0.0563 | 0.0088 | 1.58E-10 | 443,540 | 0.001348954 | 599.1205769 | Asian Genetic Epidemiology Network |
|  | rs1388657 | A | G | 0.241 | -0.0866 | 0.0135 | 1.41E-10 | 443,540 | 0.002743624 | 1220.249421 | Asian Genetic Epidemiology Network |
|  | rs1403479 | T | C | 0.3173 | 0.063 | 0.0092 | 7.50E-12 | 443,540 | 0.001719535 | 763.9929145 | Asian Genetic Epidemiology Network |
|  | rs1404415 | A | G | 0.3709 | 0.0728 | 0.009 | 6.02E-16 | 443,540 | 0.002473257 | 1099.703378 | Asian Genetic Epidemiology Network |
|  | rs1412172 | T | C | 0.5692 | -0.0734 | 0.009 | 3.48E-16 | 443,540 | 0.002642182 | 1175.012641 | Asian Genetic Epidemiology Network |
|  | rs1415035 | T | C | 0.2857 | 0.0641 | 0.0095 | 1.51E-11 | 443,540 | 0.001677015 | 745.0693682 | Asian Genetic Epidemiology Network |
|  | rs17126254 | A | G | 0.586 | -0.0521 | 0.0084 | 5.56E-10 | 443,540 | 0.001317053 | 584.9336408 | Asian Genetic Epidemiology Network |
|  | rs17406632 | T | C | 0.484 | 0.0614 | 0.008 | 1.65E-14 | 443,540 | 0.00188305 | 836.7798316 | Asian Genetic Epidemiology Network |
|  | rs17408630 | T | C | 0.6188 | -0.0856 | 0.0081 | 4.20E-26 | 443,540 | 0.003456851 | 1538.563581 | Asian Genetic Epidemiology Network |
|  | rs17412764 | A | G | 0.5272 | -0.0647 | 0.0081 | 1.38E-15 | 443,540 | 0.002086851 | 927.5333099 | Asian Genetic Epidemiology Network |
|  | rs17420537 | T | C | 0.4656 | -0.0615 | 0.008 | 1.50E-14 | 443,540 | 0.001882173 | 836.3896885 | Asian Genetic Epidemiology Network |
|  | rs1782586 | A | G | 0.7047 | -0.0664 | 0.0105 | 2.55E-10 | 443,540 | 0.001834991 | 815.3843378 | Asian Genetic Epidemiology Network |
|  | rs1784403 | T | C | 0.7135 | -0.0693 | 0.0102 | 1.09E-11 | 443,540 | 0.001963428 | 872.5683313 | Asian Genetic Epidemiology Network |
|  | rs1785791 | A | G | 0.8877 | -0.0826 | 0.0144 | 9.69E-09 | 443,540 | 0.001360304 | 604.1684954 | Asian Genetic Epidemiology Network |
|  | rs1786624 | T | C | 0.11 | 0.0874 | 0.0152 | 8.92E-09 | 443,540 | 0.001495669 | 664.3798216 | Asian Genetic Epidemiology Network |
|  | rs185526062 | A | G | 0.7052 | -0.1285 | 0.0087 | 2.28E-49 | 443,540 | 0.006865561 | 3066.188323 | Asian Genetic Epidemiology Network |
|  | rs2193597 | T | C | 0.9178 | -0.0976 | 0.0156 | 3.94E-10 | 443,540 | 0.001437307 | 638.4178175 | Asian Genetic Epidemiology Network |
|  | rs2195538 | A | G | 0.9198 | -0.1016 | 0.0151 | 1.71E-11 | 443,540 | 0.001522948 | 676.51578 | Asian Genetic Epidemiology Network |
|  | rs2203154 | T | C | 0.9175 | -0.1024 | 0.015 | 8.69E-12 | 443,540 | 0.001587413 | 705.1974232 | Asian Genetic Epidemiology Network |
|  | rs2215089 | A | G | 0.9035 | -0.0899 | 0.0141 | 1.82E-10 | 443,540 | 0.001409305 | 625.9622862 | Asian Genetic Epidemiology Network |
|  | rs2236091 | T | C | 0.0361 | 0.1267 | 0.0216 | 4.47E-09 | 443,540 | 0.001117178 | 496.0651263 | Asian Genetic Epidemiology Network |
|  | rs2247371 | T | C | 0.9639 | -0.1248 | 0.0215 | 6.45E-09 | 443,540 | 0.001083923 | 481.2826206 | Asian Genetic Epidemiology Network |
|  | rs2269604 | A | G | 0.9644 | -0.1216 | 0.0215 | 1.55E-08 | 443,540 | 0.001015323 | 450.7921579 | Asian Genetic Epidemiology Network |
|  | rs2270241 | T | G | 0.0356 | 0.1199 | 0.0215 | 2.45E-08 | 443,540 | 0.000987133 | 438.2635108 | Asian Genetic Epidemiology Network |
|  | rs2625152 | T | C | 0.0946 | -0.0964 | 0.0141 | 8.09E-12 | 443,540 | 0.0015919 | 707.193773 | Asian Genetic Epidemiology Network |
|  | rs2634124 | A | G | 0.8985 | 0.1072 | 0.0137 | 5.08E-15 | 443,540 | 0.00209606 | 931.6349799 | Asian Genetic Epidemiology Network |
|  | rs2642037 | A | G | 0.9151 | 0.1154 | 0.015 | 1.43E-14 | 443,540 | 0.002069273 | 919.7044711 | Asian Genetic Epidemiology Network |
|  | rs2648726 | T | C | 0.9061 | 0.087 | 0.0147 | 3.25E-09 | 443,540 | 0.001287983 | 572.0062603 | Asian Genetic Epidemiology Network |
|  | rs2660580 | T | C | 0.8487 | 0.0682 | 0.0116 | 4.12E-09 | 443,540 | 0.001194516 | 530.4467473 | Asian Genetic Epidemiology Network |
|  | rs2665936 | A | G | 0.9139 | 0.0887 | 0.0145 | 9.52E-10 | 443,540 | 0.001238167 | 549.8547233 | Asian Genetic Epidemiology Network |
|  | rs2680815 | T | C | 0.0946 | -0.0802 | 0.0139 | 7.94E-09 | 443,540 | 0.001101819 | 489.2377616 | Asian Genetic Epidemiology Network |
|  | rs2685820 | T | C | 0.0889 | -0.0942 | 0.0146 | 1.10E-10 | 443,540 | 0.001437473 | 638.4915856 | Asian Genetic Epidemiology Network |
|  | rs2696397 | T | C | 0.0992 | -0.0893 | 0.0136 | 5.16E-11 | 443,540 | 0.001425191 | 633.0283946 | Asian Genetic Epidemiology Network |
|  | rs2711018 | A | G | 0.1008 | -0.0896 | 0.0137 | 6.15E-11 | 443,540 | 0.001455335 | 646.436966 | Asian Genetic Epidemiology Network |
|  | rs2720835 | A | G | 0.1032 | -0.0846 | 0.0138 | 8.76E-10 | 443,540 | 0.001324787 | 588.372792 | Asian Genetic Epidemiology Network |
|  | rs2726233 | T | C | 0.1002 | -0.0862 | 0.0139 | 5.59E-10 | 443,540 | 0.001339856 | 595.0745185 | Asian Genetic Epidemiology Network |
|  | rs2729340 | T | C | 0.1412 | -0.0903 | 0.012 | 5.27E-14 | 443,540 | 0.001977572 | 878.8661979 | Asian Genetic Epidemiology Network |
|  | rs2729947 | T | G | 0.7853 | 0.0609 | 0.0106 | 9.18E-09 | 443,540 | 0.00125064 | 555.4008531 | Asian Genetic Epidemiology Network |
|  | rs2732971 | T | C | 0.9 | 0.088 | 0.014 | 3.26E-10 | 443,540 | 0.00139392 | 619.119492 | Asian Genetic Epidemiology Network |
|  | rs2825871 | T | C | 0.3955 | -0.1582 | 0.0084 | 4.03E-79 | 443,540 | 0.011967013 | 5372.112962 | Asian Genetic Epidemiology Network |
|  | rs2834461 | A | C | 0.0209 | 0.2346 | 0.0321 | 2.70E-13 | 443,540 | 0.002252472 | 1001.312231 | Asian Genetic Epidemiology Network |
|  | rs2836085 | A | G | 0.6147 | 0.1894 | 0.0086 | 1.72E-107 | 443,540 | 0.0169923 | 7667.01091 | Asian Genetic Epidemiology Network |
|  | rs2851113 | T | C | 0.385 | -0.1883 | 0.0085 | 9.77E-109 | 443,540 | 0.01679061 | 7574.453388 | Asian Genetic Epidemiology Network |
|  | rs2868868 | A | G | 0.0554 | 0.1261 | 0.0197 | 1.54E-10 | 443,540 | 0.001664247 | 739.3874659 | Asian Genetic Epidemiology Network |
|  | rs41403944 | T | C | 0.8947 | 0.0735 | 0.0134 | 4.13E-08 | 443,540 | 0.001017913 | 451.942949 | Asian Genetic Epidemiology Network |
|  | rs41507350 | T | C | 0.4218 | 0.0708 | 0.0082 | 5.92E-18 | 443,540 | 0.002445013 | 1087.114187 | Asian Genetic Epidemiology Network |
|  | rs4268029 | A | G | 0.6154 | -0.0516 | 0.0083 | 5.07E-10 | 443,540 | 0.001260365 | 559.7250182 | Asian Genetic Epidemiology Network |
|  | rs55809138 | A | C | 0.5051 | 0.081 | 0.008 | 4.28E-24 | 443,540 | 0.003280159 | 1459.662954 | Asian Genetic Epidemiology Network |
|  | rs57759769 | T | C | 0.8052 | -0.0761 | 0.01 | 2.74E-14 | 443,540 | 0.001816737 | 807.2584099 | Asian Genetic Epidemiology Network |
|  | rs57782738 | A | G | 0.2124 | 0.0789 | 0.01 | 3.02E-15 | 443,540 | 0.002082784 | 925.7219056 | Asian Genetic Epidemiology Network |
|  | rs57869750 | T | C | 0.2159 | 0.0779 | 0.0097 | 9.67E-16 | 443,540 | 0.002054608 | 913.1730038 | Asian Genetic Epidemiology Network |
|  | rs57909385 | A | G | 0.2306 | 0.0695 | 0.0096 | 4.50E-13 | 443,540 | 0.001714001 | 761.5298715 | Asian Genetic Epidemiology Network |
|  | rs610603 | T | C | 0.086 | -0.1034 | 0.0145 | 9.96E-13 | 443,540 | 0.001680799 | 746.7532644 | Asian Genetic Epidemiology Network |
|  | rs622723 | A | G | 0.0887 | -0.1029 | 0.0141 | 2.92E-13 | 443,540 | 0.001711771 | 760.5374758 | Asian Genetic Epidemiology Network |
|  | rs62362195 | A | G | 0.2088 | -0.0648 | 0.0099 | 5.93E-11 | 443,540 | 0.001387384 | 616.212588 | Asian Genetic Epidemiology Network |
|  | rs62388784 | A | G | 0.1975 | -0.077 | 0.0101 | 2.46E-14 | 443,540 | 0.001879419 | 835.1633162 | Asian Genetic Epidemiology Network |
|  | rs62390553 | A | G | 0.8025 | 0.0765 | 0.0101 | 3.61E-14 | 443,540 | 0.00185509 | 824.3321618 | Asian Genetic Epidemiology Network |
|  | rs623976 | T | C | 0.0887 | -0.1035 | 0.0141 | 2.13E-13 | 443,540 | 0.001731792 | 769.4480065 | Asian Genetic Epidemiology Network |
|  | rs62399564 | T | C | 0.2652 | -0.0803 | 0.0091 | 1.10E-18 | 443,540 | 0.002513065 | 1117.448126 | Asian Genetic Epidemiology Network |
|  | rs628504 | A | G | 0.0893 | -0.1028 | 0.0141 | 3.08E-13 | 443,540 | 0.00171887 | 763.6968377 | Asian Genetic Epidemiology Network |
|  | rs629510 | A | C | 0.911 | 0.1036 | 0.0141 | 2.02E-13 | 443,540 | 0.001740435 | 773.2950744 | Asian Genetic Epidemiology Network |
|  | rs629601 | A | G | 0.089 | -0.1036 | 0.0141 | 2.02E-13 | 443,540 | 0.001740435 | 773.2950744 | Asian Genetic Epidemiology Network |
|  | rs629831 | T | C | 0.0889 | -0.1036 | 0.0141 | 2.02E-13 | 443,540 | 0.001738671 | 772.5096268 | Asian Genetic Epidemiology Network |
|  | rs629959 | T | C | 0.089 | -0.1038 | 0.0141 | 1.82E-13 | 443,540 | 0.001747162 | 776.2888823 | Asian Genetic Epidemiology Network |
|  | rs630782 | A | G | 0.8879 | 0.0991 | 0.0141 | 2.09E-12 | 443,540 | 0.001955001 | 868.8157479 | Asian Genetic Epidemiology Network |
|  | rs630788 | A | G | 0.1284 | -0.0899 | 0.0139 | 9.96E-11 | 443,540 | 0.001808971 | 803.8014696 | Asian Genetic Epidemiology Network |
|  | rs631462 | A | G | 0.911 | 0.1042 | 0.0141 | 1.47E-13 | 443,540 | 0.001760653 | 782.2939416 | Asian Genetic Epidemiology Network |
|  | rs632028 | T | C | 0.9107 | 0.1031 | 0.0141 | 2.63E-13 | 443,540 | 0.001728917 | 768.1684473 | Asian Genetic Epidemiology Network |
|  | rs632348 | A | G | 0.089 | -0.1044 | 0.0141 | 1.32E-13 | 443,540 | 0.001767418 | 785.3051935 | Asian Genetic Epidemiology Network |
|  | rs634905 | T | C | 0.089 | -0.1045 | 0.0141 | 1.25E-13 | 443,540 | 0.001770806 | 786.8130002 | Asian Genetic Epidemiology Network |
|  | rs638144 | T | G | 0.9112 | 0.1049 | 0.0141 | 1.01E-13 | 443,540 | 0.001780769 | 791.2478636 | Asian Genetic Epidemiology Network |
|  | rs638560 | T | C | 0.0887 | -0.1053 | 0.0141 | 8.14E-14 | 443,540 | 0.001792552 | 796.4926196 | Asian Genetic Epidemiology Network |
|  | rs639900 | T | C | 0.9112 | 0.1048 | 0.0141 | 1.06E-13 | 443,540 | 0.001777376 | 789.7373223 | Asian Genetic Epidemiology Network |
|  | rs640701 | A | G | 0.0888 | -0.1048 | 0.0141 | 1.06E-13 | 443,540 | 0.001777376 | 789.7373223 | Asian Genetic Epidemiology Network |
|  | rs642253 | T | C | 0.0888 | -0.1046 | 0.0141 | 1.19E-13 | 443,540 | 0.001770598 | 786.7205925 | Asian Genetic Epidemiology Network |
|  | rs642846 | A | G | 0.9112 | 0.1045 | 0.0141 | 1.25E-13 | 443,540 | 0.001767214 | 785.2144039 | Asian Genetic Epidemiology Network |
|  | rs644953 | A | G | 0.0888 | -0.1054 | 0.0141 | 7.71E-14 | 443,540 | 0.001797786 | 798.8223349 | Asian Genetic Epidemiology Network |
|  | rs646767 | A | G | 0.9112 | 0.1045 | 0.0142 | 1.85E-13 | 443,540 | 0.001767214 | 785.2144039 | Asian Genetic Epidemiology Network |
|  | rs647577 | A | G | 0.0895 | -0.1055 | 0.0142 | 1.09E-13 | 443,540 | 0.001814003 | 806.0412372 | Asian Genetic Epidemiology Network |
|  | rs647861 | T | C | 0.0896 | -0.1056 | 0.0142 | 1.03E-13 | 443,540 | 0.001819274 | 808.3877892 | Asian Genetic Epidemiology Network |
|  | rs650974 | T | G | 0.085 | -0.1103 | 0.0147 | 6.22E-14 | 443,540 | 0.001892435 | 840.9584273 | Asian Genetic Epidemiology Network |
|  | rs651030 | T | G | 0.9105 | 0.1047 | 0.0142 | 1.67E-13 | 443,540 | 0.001786596 | 793.8414672 | Asian Genetic Epidemiology Network |
|  | rs651407 | T | C | 0.9109 | 0.1053 | 0.0142 | 1.21E-13 | 443,540 | 0.001799845 | 799.7391285 | Asian Genetic Epidemiology Network |
|  | rs651430 | T | C | 0.9108 | 0.1049 | 0.0142 | 1.50E-13 | 443,540 | 0.001788005 | 794.4688945 | Asian Genetic Epidemiology Network |
|  | rs652247 | A | G | 0.0897 | -0.1064 | 0.0143 | 1.00E-13 | 443,540 | 0.001848801 | 821.5324804 | Asian Genetic Epidemiology Network |
|  | rs653575 | T | C | 0.0899 | -0.1054 | 0.0143 | 1.70E-13 | 443,540 | 0.001817858 | 807.7576163 | Asian Genetic Epidemiology Network |
|  | rs6943989 | T | G | 0.8994 | -0.1152 | 0.0132 | 2.61E-18 | 443,540 | 0.002401518 | 1067.72859 | Asian Genetic Epidemiology Network |
|  | rs6946330 | T | C | 0.098 | 0.1168 | 0.0133 | 1.61E-18 | 443,540 | 0.002411839 | 1072.328483 | Asian Genetic Epidemiology Network |
|  | rs6950894 | T | C | 0.2526 | 0.0569 | 0.0094 | 1.42E-09 | 443,540 | 0.001222478 | 542.8789998 | Asian Genetic Epidemiology Network |
|  | rs6953558 | T | C | 0.101 | 0.1192 | 0.0134 | 5.81E-19 | 443,540 | 0.002580261 | 1147.404231 | Asian Genetic Epidemiology Network |
|  | rs6961433 | T | C | 0.8618 | -0.0749 | 0.0116 | 1.07E-10 | 443,540 | 0.001336313 | 593.498655 | Asian Genetic Epidemiology Network |
|  | rs6962630 | C | G | 0.1388 | 0.0788 | 0.012 | 5.15E-11 | 443,540 | 0.001484485 | 659.4045424 | Asian Genetic Epidemiology Network |
|  | rs75851293 | T | C | 0.3758 | -0.0471 | 0.0085 | 3.00E-08 | 443,540 | 0.001040764 | 462.0994136 | Asian Genetic Epidemiology Network |
|  | rs77375735 | T | C | 0.6254 | -0.0483 | 0.0084 | 8.92E-09 | 443,540 | 0.001093075 | 485.3507633 | Asian Genetic Epidemiology Network |
|  | rs77381959 | A | G | 0.4531 | -0.0517 | 0.0084 | 7.52E-10 | 443,540 | 0.001324686 | 588.3280929 | Asian Genetic Epidemiology Network |
|  | rs77585820 | T | C | 0.3879 | 0.0742 | 0.0082 | 1.45E-19 | 443,540 | 0.002614448 | 1162.646601 | Asian Genetic Epidemiology Network |
|  | rs77635234 | A | G | 0.7718 | -0.0523 | 0.0095 | 3.69E-08 | 443,540 | 0.000963505 | 427.7630503 | Asian Genetic Epidemiology Network |
|  | rs77646504 | T | C | 0.6098 | -0.0747 | 0.0082 | 8.26E-20 | 443,540 | 0.002655497 | 1180.950026 | Asian Genetic Epidemiology Network |
|  | rs77828219 | A | G | 0.7342 | -0.0568 | 0.009 | 2.77E-10 | 443,540 | 0.001259204 | 559.2088907 | Asian Genetic Epidemiology Network |
|  | rs77828969 | T | C | 0.3916 | 0.0831 | 0.0094 | 9.54E-19 | 443,540 | 0.003290515 | 1464.286892 | Asian Genetic Epidemiology Network |
|  | rs77873412 | T | C | 0.5104 | 0.0601 | 0.008 | 5.80E-14 | 443,540 | 0.001805224 | 802.1333173 | Asian Genetic Epidemiology Network |
| T2D (adjBMI) | rs1029919 | T | C | 0.8554 | 0.0855 | 0.014 | 1.01E-09 | 278,712 | 0.001808422 | 504.9384168 | Asian Genetic Epidemiology Network |
|  | rs1033958 | T | C | 0.5619 | -0.0471 | 0.0084 | 2.06E-08 | 278,712 | 0.001092205 | 304.7412497 | Asian Genetic Epidemiology Network |
|  | rs1033970 | A | G | 0.4374 | 0.0473 | 0.0084 | 1.79E-08 | 278,712 | 0.00110111 | 307.2287151 | Asian Genetic Epidemiology Network |
|  | rs1036193 | A | G | 0.562 | -0.0472 | 0.0084 | 1.92E-08 | 278,712 | 0.001096792 | 306.0226436 | Asian Genetic Epidemiology Network |
|  | rs115001946 | A | G | 0.607 | 0.0486 | 0.0085 | 1.08E-08 | 278,712 | 0.001126896 | 314.4314711 | Asian Genetic Epidemiology Network |
|  | rs118184783 | T | C | 0.4166 | -0.1363 | 0.0084 | 3.29E-59 | 278,712 | 0.009030409 | 2539.800597 | Asian Genetic Epidemiology Network |
|  | rs118185025 | A | G | 0.4249 | -0.1325 | 0.0084 | 4.71E-56 | 278,712 | 0.00858009 | 2412.052554 | Asian Genetic Epidemiology Network |
|  | rs1196593 | T | C | 0.1713 | -0.1133 | 0.0118 | 7.86E-22 | 278,712 | 0.003644555 | 1019.48953 | Asian Genetic Epidemiology Network |
|  | rs1207881 | T | C | 0.5335 | -0.0691 | 0.0084 | 1.93E-16 | 278,712 | 0.002376688 | 663.9847801 | Asian Genetic Epidemiology Network |
|  | rs1218796 | A | G | 0.2493 | -0.0636 | 0.0098 | 8.60E-11 | 278,712 | 0.001514025 | 422.6136336 | Asian Genetic Epidemiology Network |
|  | rs12198019 | A | G | 0.5126 | 0.0559 | 0.0082 | 9.29E-12 | 278,712 | 0.001561413 | 435.8619248 | Asian Genetic Epidemiology Network |
|  | rs12199742 | T | C | 0.4848 | -0.0587 | 0.0093 | 2.76E-10 | 278,712 | 0.001721253 | 480.5575332 | Asian Genetic Epidemiology Network |
|  | rs12245132 | T | C | 0.6213 | 0.0907 | 0.0086 | 5.27E-26 | 278,712 | 0.003871161 | 1083.124217 | Asian Genetic Epidemiology Network |
|  | rs12246765 | T | C | 0.3784 | -0.0912 | 0.0087 | 1.04E-25 | 278,712 | 0.003912747 | 1094.805511 | Asian Genetic Epidemiology Network |
|  | rs12267476 | A | G | 0.7813 | -0.0765 | 0.01 | 2.01E-14 | 278,712 | 0.001999952 | 558.5235146 | Asian Genetic Epidemiology Network |
|  | rs12281632 | A | G | 0.5792 | 0.0982 | 0.0085 | 7.13E-31 | 278,712 | 0.004700643 | 1316.303643 | Asian Genetic Epidemiology Network |
|  | rs12288444 | C | G | 0.2126 | 0.0816 | 0.0101 | 6.52E-16 | 278,712 | 0.002229302 | 622.717097 | Asian Genetic Epidemiology Network |
|  | rs12289262 | T | C | 0.8522 | 0.1022 | 0.0168 | 1.18E-09 | 278,712 | 0.002631163 | 735.2660408 | Asian Genetic Epidemiology Network |
|  | rs12289775 | A | G | 0.2853 | 0.0803 | 0.0091 | 1.10E-18 | 278,712 | 0.002629582 | 734.822944 | Asian Genetic Epidemiology Network |
|  | rs12307894 | T | C | 0.4993 | 0.1291 | 0.0083 | 1.49E-54 | 278,712 | 0.008333389 | 2342.116523 | Asian Genetic Epidemiology Network |
|  | rs12309269 | T | C | 0.5689 | 0.1109 | 0.0085 | 6.60E-39 | 278,712 | 0.006032635 | 1691.560247 | Asian Genetic Epidemiology Network |
|  | rs12310737 | T | C | 0.6576 | -0.0555 | 0.0087 | 1.78E-10 | 278,712 | 0.001387112 | 387.1389849 | Asian Genetic Epidemiology Network |
|  | rs12312203 | A | G | 0.6881 | 0.0916 | 0.009 | 2.49E-24 | 278,712 | 0.003601537 | 1007.412599 | Asian Genetic Epidemiology Network |
|  | rs12320114 | A | C | 0.6584 | -0.0501 | 0.0089 | 1.81E-08 | 278,712 | 0.00112905 | 315.0331822 | Asian Genetic Epidemiology Network |
|  | rs12328738 | T | C | 0.3299 | -0.0798 | 0.0093 | 9.43E-18 | 278,712 | 0.002815514 | 786.9275499 | Asian Genetic Epidemiology Network |
|  | rs12332447 | T | C | 0.763 | -0.0876 | 0.01 | 1.95E-18 | 278,712 | 0.002775307 | 775.6586135 | Asian Genetic Epidemiology Network |
|  | rs1238521 | T | C | 0.2726 | 0.064 | 0.0094 | 9.86E-12 | 278,712 | 0.001624385 | 453.4690785 | Asian Genetic Epidemiology Network |
|  | rs1243617 | T | C | 0.3052 | 0.0637 | 0.0091 | 2.56E-12 | 278,712 | 0.00172089 | 480.456162 | Asian Genetic Epidemiology Network |
|  | rs1248051 | A | G | 0.2731 | 0.0646 | 0.0094 | 6.32E-12 | 278,712 | 0.001656881 | 462.5557917 | Asian Genetic Epidemiology Network |
|  | rs1252028 | A | G | 0.3052 | 0.0642 | 0.0091 | 1.73E-12 | 278,712 | 0.001748012 | 488.0415057 | Asian Genetic Epidemiology Network |
|  | rs1257311 | T | C | 0.7311 | -0.0651 | 0.0095 | 7.25E-12 | 278,712 | 0.001666324 | 465.1964473 | Asian Genetic Epidemiology Network |
|  | rs1286227 | A | T | 0.6073 | 0.0982 | 0.0087 | 1.52E-29 | 278,712 | 0.004599569 | 1287.869566 | Asian Genetic Epidemiology Network |
|  | rs1288126 | T | C | 0.3933 | -0.0979 | 0.0087 | 2.24E-29 | 278,712 | 0.00457397 | 1280.668946 | Asian Genetic Epidemiology Network |
|  | rs1288491 | T | C | 0.6076 | 0.0983 | 0.0087 | 1.33E-29 | 278,712 | 0.004607696 | 1290.155529 | Asian Genetic Epidemiology Network |
|  | rs1289447 | A | G | 0.3896 | -0.0983 | 0.0087 | 1.33E-29 | 278,712 | 0.004595899 | 1286.837269 | Asian Genetic Epidemiology Network |
|  | rs1289673 | T | C | 0.6084 | 0.0981 | 0.0087 | 1.73E-29 | 278,712 | 0.004585639 | 1283.951291 | Asian Genetic Epidemiology Network |
|  | rs1290788 | T | C | 0.389 | -0.0972 | 0.0087 | 5.56E-29 | 278,712 | 0.004491106 | 1257.363196 | Asian Genetic Epidemiology Network |
|  | rs1291058 | T | C | 0.3032 | -0.0867 | 0.0096 | 1.70E-19 | 278,712 | 0.003176183 | 888.0546138 | Asian Genetic Epidemiology Network |
|  | rs1294228 | T | G | 0.6076 | 0.0977 | 0.0087 | 2.91E-29 | 278,712 | 0.004551619 | 1274.382191 | Asian Genetic Epidemiology Network |
|  | rs1297380 | T | C | 0.3936 | -0.0972 | 0.0086 | 1.28E-29 | 278,712 | 0.004510003 | 1262.677549 | Asian Genetic Epidemiology Network |
|  | rs1300258 | A | G | 0.606 | 0.0975 | 0.0086 | 8.58E-30 | 278,712 | 0.004539501 | 1270.973784 | Asian Genetic Epidemiology Network |
|  | rs1313566 | T | C | 0.5634 | 0.0899 | 0.0096 | 7.64E-21 | 278,712 | 0.003976033 | 1112.583758 | Asian Genetic Epidemiology Network |
|  | rs1317192 | T | C | 0.5269 | -0.0762 | 0.0084 | 1.17E-19 | 278,712 | 0.002894817 | 809.156752 | Asian Genetic Epidemiology Network |
|  | rs1323789 | A | G | 0.5701 | 0.0928 | 0.0085 | 9.49E-28 | 278,712 | 0.004221283 | 1181.501142 | Asian Genetic Epidemiology Network |
|  | rs1324793 | A | G | 0.4302 | -0.0927 | 0.0085 | 1.08E-27 | 278,712 | 0.004212911 | 1179.148154 | Asian Genetic Epidemiology Network |
|  | rs1324993 | T | C | 0.3958 | -0.099 | 0.0087 | 5.30E-30 | 278,712 | 0.004687669 | 1312.653383 | Asian Genetic Epidemiology Network |
|  | rs1330759 | T | C | 0.4813 | 0.0724 | 0.0084 | 6.75E-18 | 278,712 | 0.002617214 | 731.3578389 | Asian Genetic Epidemiology Network |
|  | rs1333987 | T | C | 0.7128 | -0.0649 | 0.0094 | 5.05E-12 | 278,712 | 0.001724533 | 481.4749191 | Asian Genetic Epidemiology Network |
|  | rs1342521 | A | G | 0.505 | -0.0761 | 0.0084 | 1.31E-19 | 278,712 | 0.002895315 | 809.2965349 | Asian Genetic Epidemiology Network |
|  | rs1343405 | T | C | 0.4951 | 0.076 | 0.0084 | 1.46E-19 | 278,712 | 0.002887723 | 807.1680535 | Asian Genetic Epidemiology Network |
|  | rs1345956 | T | C | 0.3104 | 0.0639 | 0.0092 | 3.77E-12 | 278,712 | 0.001748037 | 488.0485827 | Asian Genetic Epidemiology Network |
|  | rs1346796 | A | G | 0.6005 | 0.0983 | 0.0087 | 1.33E-29 | 278,712 | 0.00463625 | 1298.187903 | Asian Genetic Epidemiology Network |
|  | rs1351509 | T | C | 0.3719 | 0.0705 | 0.0088 | 1.13E-15 | 278,712 | 0.002322005 | 648.6723098 | Asian Genetic Epidemiology Network |
|  | rs1351562 | A | G | 0.3022 | 0.0619 | 0.0092 | 1.72E-11 | 278,712 | 0.001615983 | 451.1195519 | Asian Genetic Epidemiology Network |
|  | rs1357051 | A | G | 0.4091 | -0.096 | 0.0087 | 2.61E-28 | 278,712 | 0.0044557 | 1247.406183 | Asian Genetic Epidemiology Network |
|  | rs1359604 | A | G | 0.4075 | -0.0956 | 0.0087 | 4.34E-28 | 278,712 | 0.004413283 | 1235.478538 | Asian Genetic Epidemiology Network |
|  | rs1365858 | T | C | 0.2173 | 0.0596 | 0.0106 | 1.88E-08 | 278,712 | 0.001208308 | 337.1748751 | Asian Genetic Epidemiology Network |
|  | rs1368342 | A | G | 0.4179 | -0.0959 | 0.0088 | 1.18E-27 | 278,712 | 0.004474424 | 1252.671827 | Asian Genetic Epidemiology Network |
|  | rs1373551 | T | C | 0.6932 | -0.0579 | 0.0103 | 1.89E-08 | 278,712 | 0.001425939 | 397.9910477 | Asian Genetic Epidemiology Network |
|  | rs1380699 | A | G | 0.074 | -0.1317 | 0.0226 | 5.63E-09 | 278,712 | 0.002377082 | 664.0952685 | Asian Genetic Epidemiology Network |
|  | rs1381798 | A | G | 0.3069 | 0.0588 | 0.0091 | 1.04E-10 | 278,712 | 0.001470881 | 410.5530208 | Asian Genetic Epidemiology Network |
|  | rs1388657 | A | G | 0.2411 | -0.094 | 0.0137 | 6.82E-12 | 278,712 | 0.00323346 | 904.1210201 | Asian Genetic Epidemiology Network |
|  | rs1394016 | A | G | 0.3182 | 0.0664 | 0.0095 | 2.76E-12 | 278,712 | 0.001913037 | 534.204442 | Asian Genetic Epidemiology Network |
|  | rs1403479 | T | C | 0.3171 | 0.066 | 0.0095 | 3.72E-12 | 278,712 | 0.001886563 | 526.7977002 | Asian Genetic Epidemiology Network |
|  | rs1404415 | A | G | 0.3703 | 0.0759 | 0.0093 | 3.31E-16 | 278,712 | 0.002686587 | 750.7958169 | Asian Genetic Epidemiology Network |
|  | rs1409302 | T | C | 0.3169 | 0.0656 | 0.0095 | 5.01E-12 | 278,712 | 0.001863134 | 520.2434895 | Asian Genetic Epidemiology Network |
|  | rs1412172 | T | C | 0.5698 | -0.0768 | 0.0092 | 6.95E-17 | 278,712 | 0.002891647 | 808.2681833 | Asian Genetic Epidemiology Network |
|  | rs1415035 | T | C | 0.2857 | 0.0682 | 0.0099 | 5.62E-12 | 278,712 | 0.001898408 | 530.1117599 | Asian Genetic Epidemiology Network |
|  | rs1415961 | T | C | 0.3415 | -0.0569 | 0.01 | 1.27E-08 | 278,712 | 0.001456133 | 406.4306188 | Asian Genetic Epidemiology Network |
|  | rs17316029 | T | C | 0.3665 | 0.0628 | 0.0085 | 1.49E-13 | 278,712 | 0.001831344 | 511.3502873 | Asian Genetic Epidemiology Network |
|  | rs17406632 | T | C | 0.4839 | 0.0666 | 0.0083 | 1.02E-15 | 278,712 | 0.002215481 | 618.8476197 | Asian Genetic Epidemiology Network |
|  | rs17408630 | T | C | 0.6186 | -0.0951 | 0.0085 | 4.66E-29 | 278,712 | 0.00426758 | 1194.514807 | Asian Genetic Epidemiology Network |
|  | rs17412764 | A | G | 0.5274 | -0.07 | 0.0084 | 7.86E-17 | 278,712 | 0.002442643 | 682.4559015 | Asian Genetic Epidemiology Network |
|  | rs17420537 | T | C | 0.4661 | -0.0657 | 0.0083 | 2.46E-15 | 278,712 | 0.002148324 | 600.0484507 | Asian Genetic Epidemiology Network |
|  | rs1784403 | T | C | 0.7141 | -0.0724 | 0.0105 | 5.38E-12 | 278,712 | 0.002140328 | 597.8103043 | Asian Genetic Epidemiology Network |
|  | rs1785791 | A | G | 0.889 | -0.0895 | 0.0149 | 1.89E-09 | 278,712 | 0.001580887 | 441.3066492 | Asian Genetic Epidemiology Network |
|  | rs1786624 | T | C | 0.1088 | 0.0939 | 0.0156 | 1.75E-09 | 278,712 | 0.001709879 | 477.3764946 | Asian Genetic Epidemiology Network |
|  | rs185526062 | A | G | 0.7059 | -0.1419 | 0.009 | 5.28E-56 | 278,712 | 0.008360514 | 2349.804509 | Asian Genetic Epidemiology Network |
|  | rs2193597 | T | C | 0.9181 | -0.0941 | 0.0161 | 5.07E-09 | 278,712 | 0.001331629 | 371.6330994 | Asian Genetic Epidemiology Network |
|  | rs2195538 | A | G | 0.9199 | -0.0949 | 0.0155 | 9.21E-10 | 278,712 | 0.001327198 | 370.3948027 | Asian Genetic Epidemiology Network |
|  | rs2195981 | T | C | 0.08 | 0.0953 | 0.0155 | 7.83E-10 | 278,712 | 0.001336884 | 373.101635 | Asian Genetic Epidemiology Network |
|  | rs2209967 | A | G | 0.0851 | 0.0905 | 0.0151 | 2.06E-09 | 278,712 | 0.001275353 | 355.9074879 | Asian Genetic Epidemiology Network |
|  | rs2210407 | T | C | 0.038 | 0.1219 | 0.0217 | 1.94E-08 | 278,712 | 0.001086416 | 303.1242684 | Asian Genetic Epidemiology Network |
|  | rs2215089 | A | G | 0.9035 | -0.0813 | 0.0145 | 2.06E-08 | 278,712 | 0.001152568 | 321.6028961 | Asian Genetic Epidemiology Network |
|  | rs2236091 | T | C | 0.0359 | 0.1266 | 0.0224 | 1.59E-08 | 278,712 | 0.001109466 | 309.5626759 | Asian Genetic Epidemiology Network |
|  | rs2247371 | T | C | 0.9641 | -0.1247 | 0.0223 | 2.25E-08 | 278,712 | 0.001076414 | 300.3306928 | Asian Genetic Epidemiology Network |
|  | rs2625152 | T | C | 0.0946 | -0.0941 | 0.0146 | 1.15E-10 | 278,712 | 0.001516844 | 423.401778 | Asian Genetic Epidemiology Network |
|  | rs2629162 | A | G | 0.9059 | 0.0958 | 0.0144 | 2.88E-11 | 278,712 | 0.001564699 | 436.7807812 | Asian Genetic Epidemiology Network |
|  | rs2634124 | A | G | 0.8984 | 0.1064 | 0.0141 | 4.48E-14 | 278,712 | 0.002066696 | 577.201881 | Asian Genetic Epidemiology Network |
|  | rs2642037 | A | G | 0.915 | 0.1136 | 0.0156 | 3.29E-13 | 278,712 | 0.002007367 | 560.5984516 | Asian Genetic Epidemiology Network |
|  | rs2648726 | T | C | 0.9058 | 0.0832 | 0.015 | 2.91E-08 | 278,712 | 0.001181299 | 329.6292586 | Asian Genetic Epidemiology Network |
|  | rs2660580 | T | C | 0.8484 | 0.0653 | 0.0119 | 4.08E-08 | 278,712 | 0.001096873 | 306.045077 | Asian Genetic Epidemiology Network |
|  | rs2665936 | A | G | 0.9137 | 0.0878 | 0.015 | 4.82E-09 | 278,712 | 0.00121572 | 339.2456604 | Asian Genetic Epidemiology Network |
|  | rs2685820 | T | C | 0.0892 | -0.0951 | 0.015 | 2.30E-10 | 278,712 | 0.001469532 | 410.1758965 | Asian Genetic Epidemiology Network |
|  | rs2696397 | T | C | 0.0995 | -0.0911 | 0.014 | 7.66E-11 | 278,712 | 0.001487214 | 415.1188633 | Asian Genetic Epidemiology Network |
|  | rs2706879 | T | C | 0.1003 | -0.0907 | 0.0141 | 1.25E-10 | 278,712 | 0.001484715 | 414.420335 | Asian Genetic Epidemiology Network |
|  | rs2711018 | A | G | 0.1011 | -0.091 | 0.0141 | 1.09E-10 | 278,712 | 0.001505135 | 420.1283918 | Asian Genetic Epidemiology Network |
|  | rs2711870 | T | C | 0.899 | 0.0926 | 0.0141 | 5.12E-11 | 278,712 | 0.001557159 | 434.6727138 | Asian Genetic Epidemiology Network |
|  | rs2718011 | T | C | 0.8997 | 0.091 | 0.0142 | 1.47E-10 | 278,712 | 0.001494553 | 417.1704587 | Asian Genetic Epidemiology Network |
|  | rs2720835 | A | G | 0.1033 | -0.0852 | 0.0142 | 1.97E-09 | 278,712 | 0.001344797 | 375.3130441 | Asian Genetic Epidemiology Network |
|  | rs2726233 | T | C | 0.1004 | -0.088 | 0.0143 | 7.56E-10 | 278,712 | 0.001398874 | 390.4262409 | Asian Genetic Epidemiology Network |
|  | rs2729340 | T | C | 0.1418 | -0.0914 | 0.0124 | 1.69E-13 | 278,712 | 0.002033233 | 567.8368858 | Asian Genetic Epidemiology Network |
|  | rs2729947 | T | G | 0.7849 | 0.0607 | 0.0109 | 2.56E-08 | 278,712 | 0.00124412 | 347.180496 | Asian Genetic Epidemiology Network |
|  | rs2732971 | T | C | 0.8998 | 0.0905 | 0.0145 | 4.34E-10 | 278,712 | 0.001476865 | 412.2259089 | Asian Genetic Epidemiology Network |
|  | rs2757684 | T | G | 0.1579 | -0.0654 | 0.0116 | 1.72E-08 | 278,712 | 0.001137447 | 317.3789428 | Asian Genetic Epidemiology Network |
|  | rs2825871 | T | C | 0.3953 | -0.1695 | 0.0087 | 1.54E-84 | 278,712 | 0.013735238 | 3881.460924 | Asian Genetic Epidemiology Network |
|  | rs2834461 | A | C | 0.021 | 0.2429 | 0.0326 | 9.27E-14 | 278,712 | 0.002425979 | 677.7888691 | Asian Genetic Epidemiology Network |
|  | rs2836085 | A | G | 0.6148 | 0.2031 | 0.0089 | 2.89E-115 | 278,712 | 0.019537544 | 5553.816968 | Asian Genetic Epidemiology Network |
|  | rs2850443 | A | G | 0.0409 | 0.1356 | 0.021 | 1.07E-10 | 278,712 | 0.001442569 | 402.6392209 | Asian Genetic Epidemiology Network |
|  | rs2851113 | T | C | 0.385 | -0.202 | 0.0088 | 1.33E-116 | 278,712 | 0.019322734 | 5491.55103 | Asian Genetic Epidemiology Network |
|  | rs2868868 | A | G | 0.0559 | 0.1322 | 0.0201 | 4.80E-11 | 278,712 | 0.001844687 | 515.0829093 | Asian Genetic Epidemiology Network |
|  | rs2910621 | T | C | 0.2884 | 0.0637 | 0.0094 | 1.23E-11 | 278,712 | 0.001665482 | 464.9609928 | Asian Genetic Epidemiology Network |
|  | rs34197105 | T | C | 0.8601 | 0.1002 | 0.0122 | 2.16E-16 | 278,712 | 0.002416196 | 675.0489442 | Asian Genetic Epidemiology Network |
|  | rs34204285 | A | G | 0.8519 | 0.1014 | 0.0123 | 1.67E-16 | 278,712 | 0.002594476 | 724.9872424 | Asian Genetic Epidemiology Network |
|  | rs41507350 | T | C | 0.4204 | 0.0748 | 0.0084 | 5.35E-19 | 278,712 | 0.002726618 | 762.0133838 | Asian Genetic Epidemiology Network |
|  | rs55809138 | A | C | 0.5054 | 0.089 | 0.0083 | 7.95E-27 | 278,712 | 0.003960038 | 1108.090284 | Asian Genetic Epidemiology Network |
|  | rs62388784 | A | G | 0.1974 | -0.0707 | 0.0105 | 1.66E-11 | 278,712 | 0.001583854 | 442.1362085 | Asian Genetic Epidemiology Network |
|  | rs62399564 | T | C | 0.2653 | -0.0791 | 0.0094 | 3.93E-17 | 278,712 | 0.002439104 | 681.4647357 | Asian Genetic Epidemiology Network |
|  | rs6943989 | T | G | 0.8992 | -0.1208 | 0.0135 | 3.61E-19 | 278,712 | 0.002645335 | 739.2368751 | Asian Genetic Epidemiology Network |
|  | rs6946330 | T | C | 0.0982 | 0.1227 | 0.0137 | 3.36E-19 | 278,712 | 0.002666495 | 745.1659162 | Asian Genetic Epidemiology Network |
|  | rs6950894 | T | C | 0.2529 | 0.0581 | 0.0097 | 2.10E-09 | 278,712 | 0.001275586 | 355.9727152 | Asian Genetic Epidemiology Network |
|  | rs6953558 | T | C | 0.1014 | 0.1245 | 0.0138 | 1.85E-19 | 278,712 | 0.002824705 | 789.5035891 | Asian Genetic Epidemiology Network |
|  | rs6962630 | C | G | 0.1395 | 0.0807 | 0.0123 | 5.35E-11 | 278,712 | 0.001563515 | 436.4497571 | Asian Genetic Epidemiology Network |
|  | rs6966326 | T | C | 0.8727 | -0.0749 | 0.0131 | 1.08E-08 | 278,712 | 0.001246485 | 347.8413766 | Asian Genetic Epidemiology Network |
|  | rs71627333 | A | G | 0.7874 | -0.0611 | 0.0102 | 2.10E-09 | 278,712 | 0.001249888 | 348.7922263 | Asian Genetic Epidemiology Network |
|  | rs75254889 | T | C | 0.031 | -0.1505 | 0.0274 | 3.96E-08 | 278,712 | 0.001360782 | 379.7802711 | Asian Genetic Epidemiology Network |
|  | rs75851293 | T | C | 0.3753 | -0.0526 | 0.0088 | 2.27E-09 | 278,712 | 0.001297333 | 362.0494534 | Asian Genetic Epidemiology Network |
|  | rs77297139 | T | C | 0.3502 | 0.048 | 0.0088 | 4.91E-08 | 278,712 | 0.001048596 | 292.561052 | Asian Genetic Epidemiology Network |
|  | rs77375735 | T | C | 0.6252 | -0.0497 | 0.0087 | 1.11E-08 | 278,712 | 0.001157607 | 323.0107006 | Asian Genetic Epidemiology Network |
|  | rs77381959 | A | G | 0.4537 | -0.0535 | 0.0087 | 7.78E-10 | 278,712 | 0.001418853 | 396.010525 | Asian Genetic Epidemiology Network |
|  | rs77646504 | T | C | 0.6101 | -0.0783 | 0.0085 | 3.21E-20 | 278,712 | 0.002916808 | 815.3215768 | Asian Genetic Epidemiology Network |
|  | rs77828219 | A | G | 0.7344 | -0.0621 | 0.0093 | 2.43E-11 | 278,712 | 0.001504437 | 419.9333309 | Asian Genetic Epidemiology Network |
|  | rs77828969 | T | C | 0.3916 | 0.0858 | 0.0096 | 3.98E-19 | 278,712 | 0.003507813 | 981.1041512 | Asian Genetic Epidemiology Network |
|  | rs77873412 | T | C | 0.5105 | 0.0645 | 0.0083 | 7.78E-15 | 278,712 | 0.002079208 | 580.7033712 | Asian Genetic Epidemiology Network |
| Metabolic syndrome | rs651821 | C | T | NA | 0.2468 | 0.02936 | 4.20E-17 | 11,929 | NA | 70.66074438 | Zhu, Y. et al study |
|  | rs671 | A | G | NA | -0.3425 | 0.03122 | 5.40E-28 | 12,440 | NA | 120.352566 | Zhu, Y. et al study |
| Gout | rs1260326 | T | C | NA | 0.27002714 | 0.04143656 | 7.19E-11 | 2,158 | NA | 42.46659458 | Nakayama, A. et al study |
|  | rs1014290 | T | C | NA | 0.45107562 | 0.02565137 | 6.50E-26 | 2,158 | NA | 309.2271213 | Nakayama, A. et al study |
|  | rs3114020 | G | A | NA | 0.63657683 | 0.02194696 | 8.66E-35 | 2,158 | NA | 841.3039129 | Nakayama, A. et al study |
|  | rs2285340 | A | G | NA | 0.33647224 | 0.04101876 | 4.61E-11 | 2,158 | NA | 67.28734867 | Nakayama, A. et al study |
|  | rs4073582 | C | T | NA | 0.45742485 | 0.0293915 | 4.03E-20 | 2,158 | NA | 242.2121926 | Nakayama, A. et al study |
|  | rs4766566 | T | C | NA | 0.41210965 | 0.04627983 | 5.39E-09 | 2,158 | NA | 79.29431658 | Nakayama, A. et al study |
| Graves' disease | rs1061537 | A | G | 0.623573 | -0.314305 | 0.0320637 | 1.10E-22 | 212,453 | 0.046376786 | 10331.95746 | Biobank Japan |
|  | rs11065783 | G | A | 0.269497 | 0.290361 | 0.0387993 | 7.23E-14 | 212,453 | 0.033195751 | 7294.620969 | Biobank Japan |
|  | rs11571292 | A | G | 0.613598 | 0.244661 | 0.0316649 | 1.10E-14 | 212,453 | 0.028384601 | 6206.506005 | Biobank Japan |
|  | rs117201373 | G | A | 0.065342 | 0.470098 | 0.0645548 | 3.29E-13 | 212,453 | 0.02699305 | 5893.791807 | Biobank Japan |
|  | rs13136820 | T | C | 0.719252 | -0.202069 | 0.0350801 | 8.40E-09 | 212,453 | 0.016490246 | 3562.10927 | Biobank Japan |
|  | rs148781980 | G | A | 0.177321 | 0.286346 | 0.0408256 | 2.32E-12 | 212,453 | 0.023922294 | 5206.875658 | Biobank Japan |
|  | rs1569723 | A | C | 0.615164 | 0.185733 | 0.0315779 | 4.06E-09 | 212,453 | 0.01633333 | 3527.650658 | Biobank Japan |
|  | rs2049218 | T | C | 0.38865 | -0.17314 | 0.0317374 | 4.89E-08 | 212,453 | 0.014245359 | 3070.176619 | Biobank Japan |
|  | rs2456453 | T | C | 0.340589 | -0.20771 | 0.0347851 | 2.35E-09 | 212,453 | 0.019379011 | 4198.452144 | Biobank Japan |
|  | rs4248153 | G | A | 0.62052 | -0.267266 | 0.0317584 | 3.91E-17 | 212,453 | 0.033640473 | 7395.74871 | Biobank Japan |
|  | rs4903961 | G | C | 0.613356 | 0.234352 | 0.0318771 | 1.96E-13 | 212,453 | 0.02604901 | 5682.152605 | Biobank Japan |
|  | rs9296074 | G | A | 0.468856 | 0.44147 | 0.0309941 | 4.91E-46 | 212,453 | 0.097069803 | 22839.61341 | Biobank Japan |
|  | rs9319588 | T | C | 0.905526 | -0.304726 | 0.0531149 | 9.63E-09 | 212,453 | 0.015887744 | 3429.859934 | Biobank Japan |

| **Supplementary Table 2. Potential bias in UVMR estimates due to sample overlap between GWAS of metabolic risk factors and digestive system cancers based on BBJ.** | | | | | |
| --- | --- | --- | --- | --- | --- |
|  |  | Esophageal cancer (n=1300) | |  |  |
| metabolic risk factors | average F | N of metabolic risk factor | Maximum sample overlap | β | Maximum bias |
| BMI | 59.4640445 | 158284 | 0.008213085 | -0.010050336 | -1.38814E-06 |
| uric acid | 181.8545165 | 109029 | 0.011923433 | 0.009950331 | 6.52401E-07 |
| creatinine | 55.04625352 | 142097 | 0.00914868 | -0.040821995 | -6.78461E-06 |
| Graves’ disease | 6818.42425 | 212,453 | 0.006119 | -0.010050336 | -9.01939E-09 |
|  |  | Gastric cancer (n=6,563) |  |  |  |
| metabolic risk factors | average F | N of metabolic risk factor | Maximum sample overlap | β | Maximum bias |
| BMI | 59.4640445 | 158284 | 0.041463445 | 0.076961041 | 5.36639E-05 |
| uric acid | 181.8545165 | 109029 | 0.060194994 | -0.061875404 | -2.04811E-05 |
| creatinine | 55.04625352 | 142097 | 0.04618676 | -0.072570693 | -6.08907E-05 |
| Graves’ disease | 6818.42425 | 212,453 | 0.030891538 | 0.019802627 | 8.97177E-08 |
|  |  | Colorectal cancer (n=7,062 ) | |  |  |
| metabolic risk factors | average F | N of metabolic risk factor | Maximum sample overlap | β | Maximum bias |
| BMI | 59.4640445 | 158284 | 0.044616007 | 0.139761942 | 0.000104864 |
| uric acid | 181.8545165 | 109029 | 0.064771758 | -0.094310679 | -3.3591E-05 |
| creatinine | 55.04625352 | 142097 | 0.049698445 | 0.067658648 | 6.10855E-05 |
| Graves’ disease | 6818.42425 | 212,453 | 0.033240293 | -0.020202707 | -9.84896E-08 |
|  |  | Hepatocellular carcinoma(n=1,866) | |  |  |
| metabolic risk factors | average F | N of metabolic risk factor | Maximum sample overlap | β | Maximum bias |
| BMI | 59.4640445 | 158284 | 0.011788936 | -0.061875404 | -1.2267E-05 |
| uric acid | 181.8545165 | 109029 | 0.017114713 | 0.173953307 | 1.63711E-05 |
| creatinine | 55.04625352 | 142097 | 0.013131875 | -0.061875404 | -1.4761E-05 |
| Graves’ disease | 6818.42425 | 212,453 | 0.008783119 | -0.020202707 | -2.6024E-08 |
|  |  | Biliary tract cancer (n=339) |  |  |  |
| metabolic risk factors | average F | N of metabolic risk factor | Maximum sample overlap | β | Maximum bias |
| BMI | 59.4640445 | 158284 | 0.00214172 | -0.072570693 | -2.61378E-06 |
| uric acid | 181.8545165 | 109029 | 0.003109265 | 0.223143551 | 3.81521E-06 |
| creatinine | 55.04625352 | 142097 | 0.002385694 | -0.083381609 | -3.61374E-06 |
| Graves’ disease | 6818.42425 | 212,453 | 0.001595647 | -0.127833372 | -2.99156E-08 |
|  |  | Pancreatic carcinoma (n=442) | |  |  |
| metabolic risk factors | average F | N of metabolic risk factor | Maximum sample overlap | β | Maximum bias |
| BMI | 59.4640445 | 158284 | 0.002792449 | -0.261364764 | -1.22738E-05 |
| uric acid | 181.8545165 | 109029 | 0.004053967 | -0.527632742 | -1.17622E-05 |
| creatinine | 55.04625352 | 142097 | 0.003110551 | -0.356674944 | -2.0155E-05 |
| Graves’ disease | 6818.42425 | 212,453 | 0.00208046 | 0.113328685 | 3.45792E-08 |
|  |  |  |  |  |  |
| The bias was calculated as βr/F, where β was plugged-in with the MR-Corr estimate and r was the sample overlap rate between GWAS of the exposure | | | | | |
| (study 1) and the outcome (study 2) and F was the mean F statistic averaged across IVs. The maximum sample overlap rate was calculated as | | | | | |
| min(n1,n2)⁄max(n1,n2)，where n1 and n2 were the sample sizes of study 1 and 2, respectively. | | | | | |

| **Supplementary Table 3. MR analyses of the causal effect of metabolic factors on esophageal cancer** | | | | | | | | | | | |
| --- | --- | --- | --- | --- | --- | --- | --- | --- | --- | --- | --- |
| Trait |  | MR-Egger |  | Weighted median |  | IVW/WR |  | MR-PRESSO | P pleiotropy | Q | P heterogeneity test |
|  | SNP | OR(95%CI) | p | OR(95%CI) | p | OR(95%CI) | p | Global Test P |  |  |  |
| **Life style factors** |  |  |  |  |  |  |  |  |  |  |  |
| Ever/never drinkers | 3 | 11.86(3.49E-9 - 4.02E+10) | 0.8620 | 3061.45(23.07-4.06E+5) | 0.0013 | 22406.01(40.02-1.25E+7) | 0.0019 | N/A | 0.605 | 5.92 | 0.052 |
| Sweet taste | 2 | N/A | N/A | N/A | N/A | 0.60(0.01-28.64) | 0.0793 | N/A | N/A | 15.79 | 0.000 |
| Coffee consumption | 5 | 0.95(0.17-4.88) | 0.9574 | 1.18(0.83-1.68) | 0.3529 | 1.51(0.78-2.91) | 0.2192 | 0.104 | 0.582 | 19.28 | 0.001 |
| **Physical condition** |  |  |  |  |  |  |  |  |  |  |  |
| BMI | 57 | 0.73(0.11-4.77) | 0.7452 | 0.73(0.41-1.30) | 0.2833 | 0.99(0.52-1.90) | 0.9769 | 0.695 | 0.737 | 165.53 | 0.000 |
| Waist circumference (adjBMI) | 4 | 0.96(0.87-1.07) | 0.5478 | 0.98(0.96-1.00) | 0.0706 | 0.99(0.97-1.00) | 0.0764 | 0.614 | 0.702 | 2.12 | 0.548 |
| Waist-hip ratio (adjBMI) | 2 | N/A | N/A | N/A | N/A | 1.01(0.99-1.03) | 0.2276 | N/A | N/A | 0.01 | 0.922 |
| Education status | 3 | 0.45(0.06-3.56) | 0.5889 | 1.19(0.66-2.14) | 0.5597 | 1.19(0.71-1.99) | 0.5084 | N/A | 0.517 | 0.90 | 0.638 |
| **Serum parameters** |  |  |  |  |  |  |  |  |  |  |  |
| HDL-C | 15 | 0.95(0.49-1.84) | 0.8893 | 1.05(0.79-1.40) | 0.7370 | 0.96(0.73-1.26) | 0.7437 | 0.294 | 0.995 | 25.28 | 0.021 |
| LDL-C | 12 | 0.73(0.50-1.07) | 0.1398 | 0.78(0.60-1.02) | 0.0700 | 0.85(0.66-1.10) | 0.2203 | 0.228 | 0.319 | 15.74 | 0.151 |
| TC-C | 16 | 0.58(0.27-1.26) | 0.1910 | 0.77(0.53-1.11) | 0.1636 | 0.82(0.57-1.18) | 0.2884 | 0.367 | 0.339 | 30.15 | 0.011 |
| TG-C | 9 | 1.67(0.66-4.22) | 0.3123 | 1.50(1.12-2.01) | 0.0058 | 1.05(0.65-1.70) | 0.8306 | 0.591 | 0.294 | 37.06 | 0.000 |
| Glycine | 1 | N/A | N/A | N/A | N/A | 1.19(0.92-1.54) | 0.1847 | N/A | N/A | N/A | N/A |
| Uric acid | 24 | 1.10(0.69-1.76) | 0.6780 | 1.12(0.76-1.66) | 0.5694 | 1.01(0.75-1.34) | 0.9716 | 0.388 | 0.610 | 25.18 | 0.341 |
| Creatinine | 55 | 0.71(0.15-3.24) | 0.6566 | 1.18(0.67-2.08) | 0.5719 | 0.96(0.63-1.47) | 0.8478 | 0.125 | 0.683 | 66.79 | 0.114 |
| **Metabolic comorbidities** |  |  |  |  |  |  |  |  |  |  |  |
| T2D | 149 | 0.94(0.70-1.28) | 0.7104 | 1.03(0.90-1.17) | 0.6754 | 1.03(0.95-1.13) | 0.4633 | 0.429 | 0.547 | 150.71 | 0.423 |
| T2D (adjBMI) | 132 | 1.16(0.87-1.55) | 0.3118 | 1.08(0.93-1.25) | 0.3276 | 1.02(0.92-1.16) | 0.7463 | 0.529 | 0.338 | 129.32 | 0.525 |
| Metabolic syndrome | 1 | N/A | N/A | N/A | N/A | 1.61(1.15-2.25) | 0.0057 | N/A | N/A | N/A | N/A |
| Gout | 4 | 1.62(0.94-2.81) | 0.2224 | 0.97(0.85-1.10) | 0.5966 | 0.90(0.67-1.21) | 0.4713 | 0.058 | 0.149 | 22.27 | 0.000 |
| Graves' disease | 12 | 1.01(0.75-1.37) | 0.9498 | 1.01(0.89-1.14) | 0.8698 | 0.99(0.90-1.08) | 0.7567 | 0.877 | 0.872 | 6.11 | 0.866 |

| **Supplementary Table 4. MR analyses of the causal effect of metabolic factors on gastric cancer** | | | | | | | | | | | |
| --- | --- | --- | --- | --- | --- | --- | --- | --- | --- | --- | --- |
| Trait |  | MR-Egger |  | Weighted median |  | IVW/WR |  | MR-PRESSO | P pleiotropy | Q | P heterogeneity test |
|  | SNP | OR(95%CI) | p | OR(95%CI) | p | OR(95%CI) | p | Global Test P |  |  |  |
| **Life style factors** |  |  |  |  |  |  |  |  |  |  |  |
| Ever/never drinkers | 5 | 0.95(0.93-0.98) | 0.0422 | 0.95(0.93-0.98) | 0.0001 | 0.95(0.93-0.98) | 0.0000 | 0.889 | 0.823 | 0.43 | 0.979 |
| Sweet taste | 3 | 1.43(0.65-3.16) | 0.5326 | 1.44(1.21-1.71) | 0.0000 | 1.46(1.07-1.98) | 0.0144 | N/A | 0.968 | 6.46 | 0.039 |
| Coffee consumption | 6 | 1.34(1.08-1.66) | 0.0590 | 1.19(1.05-1.34) | 0.0060 | 1.12(0.96-1.30) | 0.1488 | 0.217 | 0.124 | 15.15 | 0.009 |
| **Physical condition** |  |  |  |  |  |  |  |  |  |  |  |
| BMI | 57 | 1.07(0.65-1.75) | 0.8004 | 1.07(0.83-1.39) | 0.6050 | 1.08(0.91-1.29) | 0.3560 | 0.690 | 0.942 | 49.83 | 0.706 |
| Waist circumference (adjBMI) | 4 | 0.95(0.91-1.00) | 0.1892 | 1.00(0.99-1.01) | 0.4933 | 1.00(0.99-1.01) | 0.5960 | 0.156 | 0.169 | 6.71 | 0.082 |
| Waist-hip ratio (adjBMI) | 2 | N/A | N/A | N/A | N/A | 1.00(0.99-1.01) | 0.4530 | N/A | N/A | 0.34 | 0.557 |
| Education status | 3 | 1.21(0.11-12.9) | 0.8999 | 0.81(0.60-1.08) | 0.1437 | 0.86(0.55-1.32) | 0.4857 | N/A | 0.817 | 6.88 | 0.032 |
| **Serum parameters** |  |  |  |  |  |  |  |  |  |  |  |
| HDL-C | 15 | 0.88(0.71-1.09) | 0.2707 | 0.94(0.83-1.07) | 0.3577 | 1.01(0.92-1.11) | 0.8487 | 0.265 | 0.193 | 14.94 | 0.311 |
| LDL-C | 12 | 0.96(0.79-1.17) | 0.6998 | 1.01(0.89-1.15) | 0.8828 | 0.92(0.81-1.04) | 0.1912 | 0.150 | 0.552 | 18.08 | 0.080 |
| TC-C | 16 | 0.84(0.62-1.14) | 0.2753 | 0.86(0.72-1.02) | 0.0889 | 0.88(0.76-1.01) | 0.0715 | 0.138 | 0.731 | 21.81 | 0.112 |
| TG-C | 9 | 0.84(0.68-1.02) | 0.1232 | 0.93(0.82-1.05) | 0.2389 | 0.96(0.86-1.07) | 0.4948 | 0.351 | 0.155 | 9.50 | 0.302 |
| Glycine | 1 | N/A | N/A | N/A | N/A | 0.95(0.84-1.06) | 0.3567 | N/A | N/A | N/A | N/A |
| Uric acid | 26 | 1.13(0.90-1.42) | 0.3004 | 0.97(0.81-1.14) | 0.6827 | 0.94(0.81-1.09) | 0.4183 | 0.077 | 0.052 | 36.35 | 0.066 |
| Creatinine | 55 | 1.14(0.57-2.29) | 0.7093 | 1.04(0.80-1.35) | 0.7539 | 0.93(0.76-1.13) | 0.4497 | 0.104 | 0.544 | 67.37 | 0.104 |
| **Metabolic comorbidities** |  |  |  |  |  |  |  |  |  |  |  |
| T2D | 151 | 1.05(0.92-1.21) | 0.4786 | 0.99(0.93-1.06) | 0.7931 | 0.98(0.94-1.02) | 0.2801 | 0.426 | 0.292 | 153.36 | 0.409 |
| T2D (adjBMI) | 134 | 1.04(0.91-1.19) | 0.5768 | 0.96(0.90-1.03) | 0.2472 | 0.97(0.93-1.02) | 0.2606 | 0.187 | 0.331 | 147.04 | 0.191 |
| Metabolic syndrome | 2 | N/A | N/A | N/A | N/A | 0.82(0.73-0.93) | 0.0021 | N/A | N/A | 1.77 | 0.184 |
| Gout | 6 | 0.94(0.72-1.21) | 0.6462 | 0.99(0.94-1.04) | 0.5894 | 0.98(0.92-1.05) | 0.6009 | 0.151 | 0.723 | 13.86 | 0.016 |
| Graves' disease | 13 | 1.00(0.85-1.17) | 0.9608 | 1.05(0.99-1.11) | 0.1147 | 1.02(0.98-1.07) | 0.3293 | 0.189 | 0.742 | 15.78 | 0.202 |

| **Supplementary Table 5. MR analyses of the causal effect of metabolic factors on colorectal cancer** | | | | | | | | | | | |
| --- | --- | --- | --- | --- | --- | --- | --- | --- | --- | --- | --- |
| Trait |  | MR-Egger |  | Weighted median |  | IVW/WR |  | MR-PRESSO | P pleiotropy | Q | P heterogeneity test |
|  | SNP | OR(95%CI) | p | OR(95%CI) | p | OR(95%CI) | p | Global Test P |  |  |  |
| **Life style factors** |  |  |  |  |  |  |  |  |  |  |  |
| Ever/never drinkers | 4 | 0.23(0.02-2.78) | 0.3667 | 0.73(0.26-1.99) | 0.5340 | 0.84(0.23-3.07) | 0.7952 | 0.307 | 0.362 | 5.90 | 0.117 |
| Sweet taste | 2 | N/A | N/A | N/A | N/A | 1.04(0.68-1.60) | 0.8572 | N/A | N/A | 0.62 | 0.431 |
| Coffee consumption | 5 | 0.78(0.58-1.06) | 0.2107 | 1.02(0.88-1.19) | 0.7678 | 1.04(0.88-1.21) | 0.6589 | 0.332 | 0.136 | 5.78 | 0.220 |
| **Physical condition** |  |  |  |  |  |  |  |  |  |  |  |
| BMI | 57 | 1.14(0.60-2.17) | 0.6946 | 1.12(0.86-1.45) | 0.4113 | 1.15(0.92-1.44) | 0.2138 | 0.686 | 56.000 | 101.85 | 0.000 |
| Waist circumference (adjBMI) | 4 | 0.98(0.89-1.07) | 0.6950 | 0.99(0.99-1.00) | 0.2432 | 1.00(0.99-1.01) | 0.8815 | 0.067 | 0.704 | 9.56 | 0.023 |
| Waist-hip ratio (adjBMI) | 2 | N/A | N/A | N/A | N/A | 1.00(1.00-1.01) | 0.4580 | N/A | N/A | 0.22 | 0.639 |
| Education status | 3 | 0.83(0.25-2.75) | 0.8155 | 0.92(0.70-1.19) | 0.5332 | 0.92(0.73-1.15) | 0.4556 | N/A | 0.897 | 1.79 | 0.409 |
| **Serum parameters** |  |  |  |  |  |  |  |  |  |  |  |
| HDL-C | 14 | 0.89(0.72-1.10) | 0.2969 | 0.97(0.86-1.09) | 0.6031 | 1.04(0.94-1.15) | 0.4224 | 0.219 | 0.131 | 16.62 | 0.217 |
| LDL-C | 12 | 0.92(0.70-1.21) | 0.5668 | 1.00(0.89-1.13) | 0.9788 | 1.02(0.85-1.22) | 0.8586 | 0.149 | 0.360 | 40.83 | 0.000 |
| TC-C | 16 | 0.85(0.53-1.34) | 0.4902 | 1.04(0.86-1.24) | 0.6950 | 1.08(0.87-1.35) | 0.4704 | 0.992 | 0.251 | 55.73 | 0.000 |
| TG-C | 9 | 1.00(0.78-1.27) | 0.9939 | 1.01(0.90-1.12) | 0.9088 | 0.97(0.86-1.09) | 0.5891 | 0.274 | 0.781 | 11.27 | 0.187 |
| Glycine | 1 | N/A | N/A | N/A | N/A | 1.09(0.97-1.22) | 0.1507 | N/A | N/A | N/A | N/A |
| Uric acid | 26 | 1.07(0.76-1.52) | 0.6942 | 0.91(0.77-1.07) | 0.2496 | 0.91(0.77-1.07) | 0.2469 | 0.448 | 0.250 | 83.26 | 0.000 |
| Creatinine | 51 | 1.55(0.83-2.90) | 0.1743 | 1.10(0.85-1.42) | 0.4657 | 1.07(0.90-1.29) | 0.4411 | 0.313 | 0.233 | 54.26 | 0.315 |
| **Metabolic comorbidities** |  |  |  |  |  |  |  |  |  |  |  |
| T2D | 151 | 0.94(0.83-1.08) | 0.3775 | 0.98(0.92-1.04) | 0.4449 | 0.99(0.96-1.03) | 0.7180 | 0.906 | 0.415 | 127.58 | 0.908 |
| T2D (adjBMI) | 134 | 0.99(0.87-1.12) | 0.8279 | 0.97(0.91-1.04) | 0.4095 | 0.98(0.94-1.02) | 0.2590 | 0.463 | 0.877 | 133.39 | 0.474 |
| Metabolic syndrome | 1 | N/A | N/A | N/A | N/A | 1.02(0.88-1.18) | 0.7910 | N/A | N/A | N/A | N/A |
| Gout | 4 | 0.86(0.70-1.04) | 0.2629 | 0.99(0.94-1.04) | 0.6459 | 1.00(0.95-1.06) | 0.8931 | 0.313 | 0.246 | 5.01 | 0.171 |
| Graves' disease | 10 | 0.99(0.83-1.18) | 0.9278 | 1.00(0.94-1.06) | 0.9589 | 0.98(0.93-1.03) | 0.3576 | 0.154 | 0.856 | 13.84 | 0.128 |

| **Supplementary Table 6. MR analyses of the causal effect of metabolic factors on hepatocellular carcinoma** | | | | | | | | | | | |
| --- | --- | --- | --- | --- | --- | --- | --- | --- | --- | --- | --- |
| Trait |  | MR-Egger |  | Weighted median |  | IVW/WR |  | MR-PRESSO | P pleiotropy | Q | P heterogeneity test |
|  | SNP | OR(95%CI) | p | OR(95%CI) | p | OR(95%CI) | p | Global Test P |  |  |  |
| **Life style factors** |  |  |  |  |  |  |  |  |  |  |  |
| Ever/never drinkers | 5 | 1.23(1.05-1.21) | 0.0486 | 1.11(1.07-1.16) | 0.0000 | 1.11(1.05-1.18) | 0.0003 | 0.809 | 0.575 | 8.10 | 0.088 |
| Sweet taste | 3 | 0.42(0.24-0.74) | 0.2058 | 0.41(0.29-0.58) | 0.0000 | 0.41(0.30-0.56) | 0.0000 | N/A | 0.936 | 0.05 | 0.976 |
| Coffee consumption | 6 | 0.57(0.35-0.92) | 0.0820 | 0.71(0.57-0.89) | 0.0024 | 0.69(0.53-0.90) | 0.0065 | 0.213 | 0.391 | 13.90 | 0.016 |
| **Physical condition** |  |  |  |  |  |  |  |  |  |  |  |
| BMI | 57 | 0.86(0.35-2.13) | 0.7420 | 0.94(0.58-1.53) | 0.8027 | 0.94(0.68-1.29) | 0.7046 | 0.548 | 0.833 | 53.98 | 0.552 |
| Waist circumference (adjBMI) | 4 | 1.02(0.94-1.11) | 0.6684 | 1.00(0.98-1.01) | 0.7602 | 0.99(0.98-1.01) | 0.3965 | 0.761 | 0.588 | 1.33 | 0.722 |
| Waist-hip ratio (adjBMI) | 2 | N/A | N/A | N/A | N/A | 1.00(0.96-1.03) | 0.8914 | N/A | N/A | 6.37 | 0.012 |
| Education status | 3 | 1.35(0.06-31.26) | 0.8809 | 0.88(0.53-1.46) | 0.6193 | 0.89(0.50-1.58) | 0.6862 | N/A | 0.831 | 3.62 | 0.163 |
| **Serum parameters** |  |  |  |  |  |  |  |  |  |  |  |
| HDL-C | 15 | 0.98(0.67-1.44) | 0.9268 | 1.05(0.84-1.33) | 0.6588 | 1.06(0.90-1.25) | 0.5048 | 0.590 | 0.677 | 11.21 | 0.594 |
| LDL-C | 12 | 0.86(0.62-1.20) | 0.3952 | 0.98(0.78-1.23) | 0.8470 | 0.93(0.75-1.15) | 0.5037 | 0.240 | 0.555 | 15.50 | 0.162 |
| TC-C | 16 | 0.75(0.39-1.43) | 0.3908 | 1.10(0.80-1.52) | 0.5597 | 1.04(0.76-1.41) | 0.8233 | 0.089 | 0.279 | 31.40 | 0.008 |
| TG-C | 9 | 0.87(0.48-1.58) | 0.6514 | 0.98(0.78-1.23) | 0.8487 | 1.14(0.84-1.55) | 0.3882 | 0.071 | 0.326 | 21.47 | 0.006 |
| Glycine | 1 | N/A | N/A | N/A | N/A | 1.07(0.86-1.33) | 0.5428 | N/A | N/A | N/A | N/A |
| Uric acid | 26 | 1.06(0.62-1.82) | 0.8274 | 1.07(0.77-1.48) | 0.6885 | 1.19(0.85-1.65) | 0.3101 | 0.294 | 0.610 | 52.82 | 0.001 |
| Creatinine | 55 | 0.32(0.09-1.10) | 0.0758 | 1.06(0.66-1.71) | 0.8075 | 0.94(0.66-1.34) | 0.7273 | 0.116 | 0.080 | 67.06 | 0.109 |
| **Metabolic comorbidities** |  |  |  |  |  |  |  |  |  |  |  |
| T2D | 151 | 1.19(0.91-1.56) | 0.1997 | 1.08(0.96-1.21) | 0.2143 | 1.02(0.94-1.10) | 0.6529 | 0.113 | 0.228 | 171.71 | 0.108 |
| T2D (adjBMI) | 134 | 1.10(0.85-1.42） | 0.4716 | 1.01(0.89-1.15) | 0.8652 | 0.96(0.89-1.05) | 0.4494 | 0.170 | 0.309 | 148.49 | 0.170 |
| Metabolic syndrome | 2 | N/A | N/A | N/A | N/A | 1.40(0.76-2.56) | 0.2805 | N/A | N/A | 12.48 | 0.000 |
| Gout | 6 | 0.68(0.47-0.97) | 0.1023 | 1.03(0.93-1.15) | 0.5699 | 1.06(0.92-1.23) | 0.3992 | 0.617 | 0.064 | 20.57 | 0.001 |
| Graves' disease | 13 | 1.12(0.67-1.89) | 0.6696 | 1.02(0.90-1.15) | 0.7762 | 0.98(0.84-1.13) | 0.7523 | 0.742 | 0.592 | 47.80 | 0.000 |

| **Supplementary Table 7. MR analyses of the causal effect of metabolic factors on biliary tract cancer** | | | | | | | | | | | |
| --- | --- | --- | --- | --- | --- | --- | --- | --- | --- | --- | --- |
| Trait |  | MR-Egger |  | Weighted median |  | IVW/WR |  | MR-PRESSO | P pleiotropy | Q | P heterogeneity test |
|  | SNP | OR(95%CI) | p | OR(95%CI) | p | OR(95%CI) | p | Global Test P |  |  |  |
| **Life style factors** |  |  |  |  |  |  |  |  |  |  |  |
| Ever/never drinkers | 5 | 0.98(0.88-1.09) | 0.7541 | 0.98(0.89-1.08) | 0.6454 | 0.98(0.89-1.07) | 0.6363 | 0.997 | 0.899 | 1.35 | 0.853 |
| Sweet taste | 3 | 0.81(0.21-3.06) | 0.8062 | 1.27(0.60-2.64) | 0.5304 | 1.39(0.67-2.88) | 0.3697 | N/A | 0.513 | 1.36 | 0.506 |
| Coffee consumption | 6 | 1.17(0.53-2.58) | 0.7256 | 1.03(0.68-1.57) | 0.2116 | 0.99(0.66-1.49) | 0.9568 | 0.470 | 0.651 | 6.03 | 0.304 |
| **Physical condition** |  |  |  |  |  |  |  |  |  |  |  |
| BMI | 57 | 2.93(0.35-24.27) | 0.3229 | 1.37(0.45-4.14) | 0.5823 | 0.93(0.44-1.94) | 0.8425 | 0.875 | 0.260 | 44.28 | 0.871 |
| Waist circumference (adjBMI) | 4 | 1.03(0.83-1.27) | 0.8361 | 0.99(0.96-1.03) | 0.7547 | 0.99(0.96-1.02) | 0.4084 | 0.515 | 0.753 | 2.49 | 0.477 |
| Waist-hip ratio (adjBMI) | 2 | N/A | N/A | N/A | N/A | 1.02(0.99-1.06) | 0.1526 | N/A | N/A | 1.03 | 0.310 |
| Education status | 3 | 0.06(0.001-3.15) | 0.3961 | 0.45(0.13-1.56) | 0.2110 | 0.63(0.23-1.69) | 0.3574 | N/A | 0.442 | 2.00 | 0.367 |
| **Serum parameters** |  |  |  |  |  |  |  |  |  |  |  |
| HDL-C | 14 | 1.91(0.65-5.64) | 0.2630 | 1.38(0.77-2.45) | 0.2784 | 1.27(0.79-2.02) | 0.3200 | 0.152 | 0.423 | 18.95 | 0.125 |
| LDL-C | 12 | 1.21(0.60-2.43) | 0.6134 | 1.63(0.95-2.83) | 0.0785 | 1.36(0.87-2.15) | 0.1808 | 0.327 | 0.652 | 12.76 | 0.309 |
| TC-C | 16 | 1.66(0.58-4.78) | 0.3620 | 2.10(1.03-4.28) | 0.0417 | 1.49(0.90-2.45) | 0.1193 | 0.534 | 0.819 | 13.87 | 0.535 |
| TG-C | 9 | 0.49(0.21-1.14) | 0.1421 | 0.49(0.30-0.82) | 0.0060 | 0.53(0.34-0.81) | 0.0036 | 0.926 | 0.838 | 3.37 | 0.909 |
| Glycine | 1 | N/A | N/A | N/A | N/A | 0.79(0.48-1.30) | 0.3461 | N/A | N/A | N/A | N/A |
| Uric acid | 26 | 1.82(0.65-5.14) | 0.2673 | 1.49(0.73-3.05) | 0.2692 | 1.25(0.66-2.38) | 0.4999 | 0.076 | 0.369 | 36.95 | 0.058 |
| Creatinine | 55 | 4.79(0.29-78.93) | 0.2779 | 0.96(0.32-2.94) | 0.9498 | 0.92(0.42-2.03) | 0.8330 | 0.240 | 0.234 | 60.61 | 0.250 |
| **Metabolic comorbidities** |  |  |  |  |  |  |  |  |  |  |  |
| T2D | 151 | 1.40(0.78-2.52) | 0.2674 | 1.02(0.79-1.32) | 0.8804 | 1.03(0.87-1.21) | 0.7644 | 0.813 | 0.285 | 134.91 | 0.806 |
| T2D (adjBMI) | 134 | 1.81(1.03-3.17) | 0.0413 | 1.17(0.88-1.55) | 0.2846 | 1.02(0.85-1.22) | 0.8648 | 0.761 | 0.036 | 121.15 | 0.761 |
| Metabolic syndrome | 2 | N/A | N/A | N/A | N/A | 0.69(0.36-1.32) | 0.2570 | N/A | N/A | 2.66 | 0.103 |
| Gout | 6 | 1.21(0.66-2.20) | 0.5708 | 1.01(0.83-1.23) | 0.9228 | 1.00(0.85-1.18) | 0.9587 | 0.786 | 0.565 | 2.91 | 0.713 |
| Graves' disease | 13 | 0.68(0.37-1.26) | 0.2470 | 0.82(0.65-1.04) | 0.0971 | 0.88(0.74-1.05) | 0.1461 | 0.419 | 0.418 | 12.69 | 0.392 |

| **Supplementary Table 8. MR analyses of the causal effect of metabolic factors on pancreatic carcinoma** | | | | | | | | | | | |
| --- | --- | --- | --- | --- | --- | --- | --- | --- | --- | --- | --- |
| Trait |  | MR-Egger |  | Weighted median |  | IVW/WR |  | MR-PRESSO | P pleiotropy | Q | P heterogeneity test |
|  | SNP | OR(95%CI) | p | OR(95%CI) | p | OR(95%CI) | p | Global Test P |  |  |  |
| **Life style factors** |  |  |  |  |  |  |  |  |  |  |  |
| Ever/never drinkers | 5 | 1.05(0.95-1.16) | 0.3864 | 1.01(0.93-1.09) | 0.8878 | 1.01(0.92-1.10) | 0.9119 | 0.493 | 0.164 | 4.89 | 0.299 |
| Sweet taste | 3 | 1.02(0.32-3.25) | 0.9834 | 0.94(0.48-1.86) | 0.8633 | 0.91(0.48-1.72) | 0.7709 | N/A | 0.862 | 0.85 | 0.653 |
| Coffee consumption | 6 | 0.83(0.46-1.48) | 0.5607 | 0.87(0.60-1.26) | 0.4484 | 0.91(0.66-1.26) | 0.5739 | 0.828 | 0.720 | 2.33 | 0.801 |
| **Physical condition** |  |  |  |  |  |  |  |  |  |  |  |
| BMI | 57 | 0.84(0.10-6.93) | 0.8718 | 0.51(0.18-1.44) | 0.2060 | 0.77(0.37-1.60) | 0.4815 | 0.075 | 0.931 | 71.76 | 0.076 |
| Waist circumference (adjBMI) | 4 | 0.91(0.76-1.08) | 0.3774 | 0.99(0.96-1.02) | 0.4113 | 0.99(0.96-1.02) | 0.5217 | 0.629 | 0.409 | 1.88 | 0.598 |
| Waist-hip ratio (adjBMI) | 2 | N/A | N/A | N/A | N/A | 1.02(0.99-1.04) | 0.3002 | N/A | N/A | 0.92 | 0.338 |
| Education status | 3 | 3.01(0.09-97.67) | 0.6460 | 0.51(0.19-1.40) | 0.1923 | 0.59(0.25-1.40) | 0.2308 | N/A | 0.516 | 1.31 | 0.518 |
| **Serum parameters** |  |  |  |  |  |  |  |  |  |  |  |
| HDL-C | 14 | 1.16(0.54-2.52) | 0.7124 | 1.11(0.71-1.76) | 0.6396 | 0.95(0.68-1.33) | 0.7771 | 0.876 | 0.588 | 7.24 | 0.889 |
| LDL-C | 12 | 0.88(0.51-1.53) | 0.6667 | 0.97(0.61-1.55) | 0.9028 | 0.86(0.59-1.24) | 0.4171 | 0.946 | 0.894 | 4.35 | 0.959 |
| TC-C | 16 | 0.80(0.32-2.01) | 0.6406 | 0.86(0.47-1.59) | 0.6362 | 0.82(0.53-1.27) | 0.3649 | 0.982 | 0.957 | 5.92 | 0.981 |
| TG-C | 9 | 0.99(0.41-2.43) | 0.9872 | 0.84(0.54-1.30) | 0.4316 | 0.73(0.47-1.13) | 0.1585 | 0.293 | 0.457 | 10.93 | 0.206 |
| Glycine | 1 | N/A | N/A | N/A | N/A | 1.41(0.91-2.18) | 0.1269 | N/A | N/A | N/A | N/A |
| Uric acid | 26 | 0.64(0.29-1.40) | 0.2743 | 0.79(0.43-1.45) | 0.4462 | 0.59(0.37-0.96) | 0.0346 | 0.401 | 0.824 | 27.29 | 0.341 |
| Creatinine | 55 | 2.41(0.22-27.00) | 0.4787 | 0.61(0.23-1.64) | 0.3260 | 0.70(0.35-1.38) | 0.2997 | 0.290 | 0.299 | 58.70 | 0.307 |
| **Metabolic comorbidities** |  |  |  |  |  |  |  |  |  |  |  |
| T2D | 151 | 1.61(0.96-2.69) | 0.0716 | 1.19(0.96-1.49) | 0.1132 | 1.06(0.92-1.23) | 0.0738 | 0.645 | 0.102 | 142.19 | 0.663 |
| T2D (adjBMI) | 134 | 1.39(0.83-2.33) | 0.2069 | 1.13(0.88-1.46) | 0.3798 | 1.00(0.85-1.18) | 0.9867 | 0.190 | 0.181 | 146.43 | 0.201 |
| Metabolic syndrome | 2 | N/A | N/A | N/A | N/A | 0.98(0.69-1.40) | 0.9282 | N/A | N/A | 0.16 | 0.688 |
| Gout | 6 | 1.16(0.69-1.96) | 0.6091 | 1.01(0.87-1.16) | 0.9381 | 1.01(0.87-1.16) | 0.9251 | 0.873 | 0.612 | 1.81 | 0.874 |
| Graves' disease | 13 | 0.92(0.53-1.60) | 0.7853 | 1.01(0.82(1.24) | 0.9090 | 1.12(0.95-1.31) | 0.1681 | 0.396 | 0.497 | 13.30 | 0.347 |

Supplementary Table 9. The statistical power of IVs for digestive system cancers

| **Exposure** | **rsID** | **esophageal cancer** | **gastric cancer** | **colorectal cancer** | **hepatocellular carcinoma** | **biliary tract cancer** | **pancreatic carcinoma** |
| --- | --- | --- | --- | --- | --- | --- | --- |
| Ever/never drinkers | rs1260326 | NA | 0.05 | 0.05 | 0.05 | 0.05 | 0.05 |
|  | rs1229984 | NA | 0.05 | 0.06 | 0.06 | 0.05 | 0.05 |
|  | rs3043 | NA | 0.05 | 0.05 | 0.05 | 0.05 | 0.05 |
|  | rs8187929 | NA | 0.05 | 0.05 | 0.05 | 0.05 | 0.05 |
|  | rs671 | NA | 0.98 | 1.00 | 1.00 | 0.06 | 0.05 |
| Sweet taste | rs671 | 0.51 | 1.00 | 0.07 | 0.94 | 0.16 | 0.10 |
|  | rs4552669 | 0.09 | 0.3 | 0.05 | 0.17 | 0.06 | 0.05 |
|  | rs13347339 | 0.09 | 0.31 | 0.05 | 0.18 | 0.06 | 0.05 |
| Coffee consumption | rs6681426 | 0.16 | 0.08 | 0.05 | 0.11 | 0.05 | 0.05 |
|  | rs1260326 | 0.24 | 0.10 | 0.06 | 0.15 | 0.05 | 0.05 |
|  | rs4410790 | 0.77 | 0.29 | 0.08 | 0.51 | 0.05 | 0.05 |
|  | rs671 | 0.93 | 0.43 | 0.10 | 0.71 | 0.05 | 0.05 |
|  | rs58806801 | 0.15 | 0.08 | 0.05 | 0.10 | 0.05 | 0.05 |
|  | rs5760444 | 0.15 | 0.08 | 0.05 | 0.10 | 0.05 | 0.05 |
| BMI | rs10062657 | 0.05 | 0.05 | 0.06 | 0.05 | 0.05 | 0.05 |
|  | rs1011939 | 0.05 | 0.05 | 0.05 | 0.05 | 0.05 | 0.05 |
|  | rs10208649 | 0.05 | 0.05 | 0.05 | 0.05 | 0.05 | 0.05 |
|  | rs1035491 | 0.05 | 0.05 | 0.05 | 0.05 | 0.05 | 0.05 |
|  | rs10764373 | 0.05 | 0.05 | 0.05 | 0.05 | 0.05 | 0.05 |
|  | rs10795945 | 0.05 | 0.05 | 0.05 | 0.05 | 0.05 | 0.05 |
|  | rs10835389 | 0.05 | 0.05 | 0.05 | 0.05 | 0.05 | 0.05 |
|  | rs11030100 | 0.05 | 0.05 | 0.06 | 0.05 | 0.05 | 0.05 |
|  | rs11191021 | 0.05 | 0.05 | 0.05 | 0.05 | 0.05 | 0.05 |
|  | rs11602339 | 0.05 | 0.05 | 0.05 | 0.05 | 0.05 | 0.05 |
|  | rs11642015 | 0.05 | 0.05 | 0.05 | 0.05 | 0.05 | 0.05 |
|  | rs12597682 | 0.05 | 0.05 | 0.05 | 0.05 | 0.05 | 0.05 |
|  | rs12617004 | 0.05 | 0.05 | 0.05 | 0.05 | 0.05 | 0.05 |
|  | rs1518170 | 0.05 | 0.05 | 0.05 | 0.05 | 0.05 | 0.05 |
|  | rs1568079 | 0.05 | 0.05 | 0.05 | 0.05 | 0.05 | 0.05 |
|  | rs16937956 | 0.05 | 0.05 | 0.05 | 0.05 | 0.05 | 0.05 |
|  | rs16978956 | 0.05 | 0.05 | 0.05 | 0.05 | 0.05 | 0.05 |
|  | rs1832886 | 0.05 | 0.05 | 0.05 | 0.05 | 0.05 | 0.05 |
|  | rs1907240 | 0.05 | 0.05 | 0.05 | 0.05 | 0.05 | 0.05 |
|  | rs1996023 | 0.05 | 0.05 | 0.05 | 0.05 | 0.05 | 0.05 |
|  | rs2206271 | 0.05 | 0.05 | 0.05 | 0.05 | 0.05 | 0.05 |
|  | rs2390669 | 0.05 | 0.05 | 0.05 | 0.05 | 0.05 | 0.05 |
|  | rs2540034 | 0.05 | 0.05 | 0.05 | 0.05 | 0.05 | 0.05 |
|  | rs35560038 | 0.05 | 0.05 | 0.08 | 0.05 | 0.05 | 0.05 |
|  | rs3888190 | 0.05 | 0.05 | 0.05 | 0.05 | 0.05 | 0.05 |
|  | rs3932549 | 0.05 | 0.05 | 0.05 | 0.05 | 0.05 | 0.05 |
|  | rs4357030 | 0.05 | 0.05 | 0.05 | 0.05 | 0.05 | 0.05 |
|  | rs4366055 | 0.05 | 0.05 | 0.05 | 0.05 | 0.05 | 0.05 |
|  | rs4409766 | 0.05 | 0.05 | 0.05 | 0.05 | 0.05 | 0.05 |
|  | rs4686392 | 0.05 | 0.05 | 0.06 | 0.05 | 0.05 | 0.05 |
|  | rs4790981 | 0.05 | 0.05 | 0.05 | 0.05 | 0.05 | 0.05 |
|  | rs4811309 | 0.05 | 0.05 | 0.05 | 0.05 | 0.05 | 0.05 |
|  | rs491055 | 0.05 | 0.05 | 0.05 | 0.05 | 0.05 | 0.05 |
|  | rs5015933 | 0.05 | 0.05 | 0.05 | 0.05 | 0.05 | 0.05 |
|  | rs55934576 | 0.05 | 0.05 | 0.05 | 0.05 | 0.05 | 0.05 |
|  | rs60808706 | 0.05 | 0.05 | 0.07 | 0.05 | 0.05 | 0.05 |
|  | rs62116682 | 0.05 | 0.05 | 0.05 | 0.05 | 0.05 | 0.05 |
|  | rs633715 | 0.05 | 0.05 | 0.06 | 0.05 | 0.05 | 0.05 |
|  | rs6567160 | 0.05 | 0.05 | 0.06 | 0.05 | 0.05 | 0.05 |
|  | rs6734118 | 0.05 | 0.05 | 0.05 | 0.05 | 0.05 | 0.05 |
|  | rs6881648 | 0.05 | 0.05 | 0.05 | 0.05 | 0.05 | 0.05 |
|  | rs6882046 | 0.05 | 0.05 | 0.05 | 0.05 | 0.05 | 0.05 |
|  | rs6913361 | 0.05 | 0.05 | 0.05 | 0.05 | 0.05 | 0.05 |
|  | rs6947395 | 0.05 | 0.05 | 0.05 | 0.05 | 0.05 | 0.05 |
|  | rs7020996 | 0.05 | 0.05 | 0.06 | 0.05 | 0.05 | 0.05 |
|  | rs713586 | 0.05 | 0.05 | 0.05 | 0.05 | 0.05 | 0.05 |
|  | rs7559954 | 0.05 | 0.05 | 0.05 | 0.05 | 0.05 | 0.05 |
|  | rs75766425 | 0.05 | 0.05 | 0.05 | 0.05 | 0.05 | 0.05 |
|  | rs7692081 | 0.05 | 0.05 | 0.05 | 0.05 | 0.05 | 0.05 |
|  | rs77489951 | 0.05 | 0.05 | 0.05 | 0.05 | 0.05 | 0.05 |
|  | rs77636220 | 0.05 | 0.05 | 0.05 | 0.05 | 0.05 | 0.05 |
|  | rs80234489 | 0.05 | 0.05 | 0.05 | 0.05 | 0.05 | 0.05 |
|  | rs9266629 | 0.05 | 0.05 | 0.05 | 0.05 | 0.05 | 0.05 |
|  | rs939584 | 0.05 | 0.05 | 0.06 | 0.05 | 0.05 | 0.05 |
|  | rs9397585 | 0.05 | 0.05 | 0.05 | 0.05 | 0.05 | 0.05 |
|  | rs9425762 | 0.05 | 0.05 | 0.05 | 0.05 | 0.05 | 0.05 |
|  | rs9568867 | 0.05 | 0.05 | 0.05 | 0.05 | 0.05 | 0.05 |
| Waist circumference (adjBMI) | rs3791679 | 0.09 | 0.05 | 0.05 | 0.05 | 0.06 | 0.06 |
|  | rs8030379 | 0.08 | 0.05 | 0.05 | 0.05 | 0.06 | 0.06 |
|  | rs3809128 | 0.12 | 0.05 | 0.05 | 0.05 | 0.07 | 0.07 |
|  | rs2057291 | 0.09 | 0.05 | 0.05 | 0.05 | 0.06 | 0.06 |
| Waist-hip ratio (adjBMI) | rs1982963 | 0.16 | 0.05 | 0.05 | 0.05 | 0.16 | 0.2 |
|  | rs5020946 | 0.12 | 0.05 | 0.05 | 0.05 | 0.13 | 0.15 |
| Education | rs280536 | 0.11 | 0.23 | 0.11 | 0.08 | 0.11 | 0.15 |
|  | rs77827642 | 0.12 | 0.25 | 0.12 | 0.08 | 0.12 | 0.17 |
|  | rs2688072 | 0.12 | 0.25 | 0.12 | 0.08 | 0.12 | 0.17 |
| HDL-C | rs3764261 | 0.05 | 0.05 | 0.07 | 0.06 | 0.11 | 0.05 |
|  | rs662799 | 0.05 | 0.05 | 0.07 | 0.06 | 0.09 | 0.05 |
|  | rs1800588 | 0.05 | 0.05 | 0.06 | 0.06 | 0.08 | 0.05 |
|  | rs1011685 | 0.05 | 0.05 | 0.06 | 0.05 | 0.06 | 0.05 |
|  | rs16940212 | 0.05 | 0.05 | 0.06 | 0.05 | 0.06 | 0.05 |
|  | rs1883025 | 0.05 | 0.05 | 0.05 | 0.05 | 0.06 | 0.05 |
|  | rs3786247 | 0.05 | 0.05 | 0.06 | 0.05 | 0.06 | 0.05 |
|  | rs4420638 | 0.05 | 0.05 | 0.05 | 0.05 | 0.06 | 0.05 |
|  | rs1883023 | 0.05 | 0.05 | 0.05 | 0.05 | 0.05 | 0.05 |
|  | rs11066280 | 0.05 | 0.05 | 0.05 | 0.05 | 0.06 | 0.05 |
|  | rs3760782 | 0.05 | 0.05 | 0.05 | 0.05 | 0.05 | 0.05 |
|  | rs1109166 | 0.05 | 0.05 | 0.05 | 0.05 | 0.05 | 0.05 |
|  | rs6124760 | 0.05 | 0.05 | 0.05 | 0.05 | 0.05 | 0.05 |
|  | rs948937 | 0.05 | 0.05 | 0.05 | 0.05 | 0.05 | 0.05 |
|  | rs445925 | 0.05 | 0.05 | 0.05 | 0.05 | 0.06 | 0.05 |
| LDL-C | rs445925 | 0.21 | 0.28 | 0.06 | 0.09 | 0.29 | 0.10 |
|  | rs599839 | 0.07 | 0.07 | 0.05 | 0.05 | 0.07 | 0.05 |
|  | rs10119 | 0.09 | 0.1 | 0.05 | 0.06 | 0.1 | 0.06 |
|  | rs12916 | 0.06 | 0.06 | 0.05 | 0.05 | 0.07 | 0.05 |
|  | rs2738464 | 0.06 | 0.06 | 0.05 | 0.05 | 0.07 | 0.05 |
|  | rs8051431 | 0.06 | 0.06 | 0.05 | 0.05 | 0.07 | 0.05 |
|  | rs10172650 | 0.06 | 0.06 | 0.05 | 0.05 | 0.06 | 0.05 |
|  | rs2980869 | 0.05 | 0.06 | 0.05 | 0.05 | 0.06 | 0.05 |
|  | rs174533 | 0.06 | 0.06 | 0.05 | 0.05 | 0.06 | 0.05 |
|  | rs1799955 | 0.05 | 0.06 | 0.05 | 0.05 | 0.06 | 0.05 |
|  | rs505151 | 0.05 | 0.06 | 0.05 | 0.05 | 0.06 | 0.05 |
|  | rs12117661 | 0.05 | 0.06 | 0.05 | 0.05 | 0.06 | 0.05 |
| TC-C | rs7254892 | 0.16 | 0.29 | 0.16 | 0.07 | 0.26 | 0.08 |
|  | rs599839 | 0.07 | 0.09 | 0.07 | 0.05 | 0.08 | 0.06 |
|  | rs12916 | 0.07 | 0.09 | 0.07 | 0.05 | 0.08 | 0.06 |
|  | rs2980869 | 0.06 | 0.09 | 0.06 | 0.05 | 0.07 | 0.05 |
|  | rs9958734 | 0.07 | 0.09 | 0.07 | 0.05 | 0.09 | 0.06 |
|  | rs10172650 | 0.06 | 0.07 | 0.06 | 0.05 | 0.07 | 0.05 |
|  | rs1865063 | 0.06 | 0.07 | 0.06 | 0.05 | 0.07 | 0.05 |
|  | rs1883025 | 0.06 | 0.07 | 0.06 | 0.05 | 0.07 | 0.05 |
|  | rs1122531 | 0.06 | 0.07 | 0.06 | 0.05 | 0.07 | 0.05 |
|  | rs2070895 | 0.06 | 0.07 | 0.06 | 0.05 | 0.07 | 0.05 |
|  | rs7776054 | 0.06 | 0.07 | 0.06 | 0.05 | 0.07 | 0.05 |
|  | rs10402592 | 0.06 | 0.07 | 0.06 | 0.05 | 0.07 | 0.05 |
|  | rs780092 | 0.06 | 0.07 | 0.06 | 0.05 | 0.06 | 0.05 |
|  | rs174533 | 0.06 | 0.07 | 0.06 | 0.05 | 0.06 | 0.05 |
|  | rs1800774 | 0.05 | 0.06 | 0.06 | 0.05 | 0.06 | 0.05 |
|  | rs17122278 | 0.05 | 0.06 | 0.06 | 0.05 | 0.06 | 0.05 |
| TG-C | rs662799 | 0.06 | 0.09 | 0.07 | 0.06 | 0.33 | 0.17 |
|  | rs780094 | 0.05 | 0.06 | 0.05 | 0.05 | 0.10 | 0.07 |
|  | rs17482753 | 0.05 | 0.06 | 0.05 | 0.05 | 0.09 | 0.07 |
|  | rs1051921 | 0.05 | 0.06 | 0.05 | 0.05 | 0.07 | 0.06 |
|  | rs995000 | 0.05 | 0.05 | 0.05 | 0.05 | 0.07 | 0.06 |
|  | rs1800588 | 0.05 | 0.06 | 0.05 | 0.05 | 0.08 | 0.06 |
|  | rs2954018 | 0.05 | 0.06 | 0.05 | 0.05 | 0.07 | 0.06 |
|  | rs157582 | 0.05 | 0.06 | 0.05 | 0.05 | 0.08 | 0.06 |
|  | rs7165077 | 0.05 | 0.05 | 0.05 | 0.05 | 0.06 | 0.06 |
| Glycine | rs1047891 | 0.38 | 0.12 | 0.44 | 0.11 | 0.15 | 0.55 |
| Uric acid | rs11202346 | 0.05 | 0.05 | 0.05 | 0.05 | 0.05 | 0.05 |
|  | rs11231455 | 0.05 | 0.05 | 0.08 | 0.09 | 0.06 | 0.10 |
|  | rs1165196 | 0.05 | 0.05 | 0.05 | 0.06 | 0.05 | 0.05 |
|  | rs1260326 | 0.05 | 0.05 | 0.05 | 0.05 | 0.05 | 0.05 |
|  | rs1549287 | 0.05 | 0.05 | 0.05 | 0.05 | 0.05 | 0.05 |
|  | rs16856823 | 0.05 | 0.05 | 0.05 | 0.05 | 0.05 | 0.05 |
|  | rs1805100 | 0.05 | 0.05 | 0.05 | 0.06 | 0.05 | 0.05 |
|  | rs1886603 | 0.05 | 0.05 | 0.05 | 0.05 | 0.05 | 0.05 |
|  | rs4077450 | 0.05 | 0.05 | 0.05 | 0.06 | 0.05 | 0.05 |
|  | rs4148155 | 0.05 | 0.05 | 0.08 | 0.06 | 0.05 | 0.10 |
|  | rs4724828 | 0.05 | 0.05 | 0.05 | 0.05 | 0.05 | 0.05 |
|  | rs4766566 | 0.05 | 0.05 | 0.06 | 0.06 | 0.05 | 0.06 |
|  | rs477138 | 0.05 | 0.05 | 0.13 | 0.14 | 0.08 | 0.15 |
|  | rs59350108 | 0.05 | 0.05 | 0.05 | 0.05 | 0.05 | 0.05 |
|  | rs6026578 | 0.05 | 0.05 | 0.05 | 0.05 | 0.05 | 0.05 |
|  | rs6445559 | 0.05 | 0.05 | 0.05 | 0.05 | 0.05 | 0.05 |
|  | rs6460047 | 0.05 | 0.05 | 0.05 | 0.05 | 0.05 | 0.05 |
|  | rs647304 | 0.05 | 0.05 | 0.07 | 0.07 | 0.05 | 0.07 |
|  | rs73575095 | 0.05 | 0.05 | 0.05 | 0.05 | 0.05 | 0.05 |
|  | rs7570707 | 0.05 | 0.05 | 0.05 | 0.05 | 0.05 | 0.05 |
|  | rs7679724 | 0.05 | 0.05 | 0.10 | 0.11 | 0.05 | 0.11 |
|  | rs7835379 | 0.05 | 0.05 | 0.05 | 0.05 | 0.05 | 0.05 |
|  | rs9394948 | 0.05 | 0.05 | 0.05 | 0.05 | 0.05 | 0.05 |
|  | rs9416703 | 0.05 | 0.05 | 0.05 | 0.05 | 0.05 | 0.05 |
|  | rs963837 | 0.05 | 0.05 | 0.05 | 0.05 | 0.05 | 0.05 |
|  | rs9895661 | 0.05 | 0.05 | 0.06 | 0.05 | 0.08 | 0.06 |
| Creatinine | rs10459012 | 0.05 | 0.05 | 0.05 | 0.05 | 0.05 | 0.05 |
|  | rs10518732 | 0.05 | 0.05 | 0.05 | 0.05 | 0.05 | 0.05 |
|  | rs10794486 | 0.05 | 0.05 | 0.05 | 0.05 | 0.05 | 0.05 |
|  | rs10857147 | 0.05 | 0.05 | 0.05 | 0.05 | 0.05 | 0.05 |
|  | rs11123169 | 0.05 | 0.05 | 0.05 | 0.05 | 0.05 | 0.05 |
|  | rs11742501 | 0.05 | 0.05 | 0.05 | 0.05 | 0.05 | 0.05 |
|  | rs118082206 | 0.05 | 0.05 | 0.05 | 0.05 | 0.05 | 0.05 |
|  | rs1275609 | 0.05 | 0.05 | 0.05 | 0.05 | 0.05 | 0.05 |
|  | rs12916630 | 0.05 | 0.05 | 0.05 | 0.05 | 0.05 | 0.05 |
|  | rs12935539 | 0.05 | 0.05 | 0.05 | 0.05 | 0.05 | 0.05 |
|  | rs142516820 | 0.05 | 0.05 | 0.05 | 0.05 | 0.05 | 0.05 |
|  | rs1533988 | 0.05 | 0.05 | 0.05 | 0.05 | 0.05 | 0.06 |
|  | rs16856823 | 0.05 | 0.05 | 0.05 | 0.05 | 0.05 | 0.05 |
|  | rs16942751 | 0.05 | 0.05 | 0.05 | 0.05 | 0.05 | 0.05 |
|  | rs16972495 | 0.05 | 0.05 | 0.05 | 0.05 | 0.05 | 0.05 |
|  | rs17001974 | 0.05 | 0.05 | 0.05 | 0.05 | 0.05 | 0.05 |
|  | rs1705694 | 0.05 | 0.05 | 0.05 | 0.05 | 0.05 | 0.05 |
|  | rs241812 | 0.05 | 0.05 | 0.05 | 0.05 | 0.05 | 0.05 |
|  | rs2511162 | 0.05 | 0.05 | 0.05 | 0.05 | 0.05 | 0.05 |
|  | rs2736609 | 0.05 | 0.05 | 0.05 | 0.05 | 0.05 | 0.05 |
|  | rs2781656 | 0.05 | 0.05 | 0.05 | 0.05 | 0.05 | 0.05 |
|  | rs307558 | 0.05 | 0.05 | 0.05 | 0.05 | 0.05 | 0.05 |
|  | rs316020 | 0.05 | 0.05 | 0.05 | 0.05 | 0.05 | 0.05 |
|  | rs34720381 | 0.05 | 0.05 | 0.05 | 0.05 | 0.05 | 0.05 |
|  | rs3734861 | 0.05 | 0.05 | 0.05 | 0.05 | 0.05 | 0.05 |
|  | rs3782787 | 0.05 | 0.05 | 0.05 | 0.05 | 0.05 | 0.05 |
|  | rs3812036 | 0.05 | 0.05 | 0.05 | 0.05 | 0.05 | 0.05 |
|  | rs4399402 | 0.05 | 0.05 | 0.05 | 0.05 | 0.05 | 0.05 |
|  | rs4665987 | 0.05 | 0.05 | 0.05 | 0.05 | 0.05 | 0.05 |
|  | rs4690095 | 0.05 | 0.05 | 0.05 | 0.05 | 0.05 | 0.05 |
|  | rs4715491 | 0.05 | 0.05 | 0.05 | 0.05 | 0.05 | 0.05 |
|  | rs4859682 | 0.05 | 0.05 | 0.05 | 0.05 | 0.05 | 0.05 |
|  | rs549752 | 0.05 | 0.05 | 0.05 | 0.05 | 0.05 | 0.05 |
|  | rs6026578 | 0.05 | 0.05 | 0.05 | 0.05 | 0.05 | 0.05 |
|  | rs67332916 | 0.05 | 0.05 | 0.05 | 0.05 | 0.05 | 0.05 |
|  | rs6851943 | 0.05 | 0.05 | 0.05 | 0.05 | 0.05 | 0.05 |
|  | rs6907843 | 0.05 | 0.05 | 0.05 | 0.05 | 0.05 | 0.05 |
|  | rs7123489 | 0.05 | 0.05 | 0.05 | 0.05 | 0.05 | 0.05 |
|  | rs715 | 0.05 | 0.05 | 0.05 | 0.05 | 0.05 | 0.05 |
|  | rs716877 | 0.05 | 0.05 | 0.05 | 0.05 | 0.05 | 0.05 |
|  | rs7177266 | 0.05 | 0.05 | 0.05 | 0.05 | 0.05 | 0.05 |
|  | rs7212715 | 0.05 | 0.05 | 0.05 | 0.05 | 0.05 | 0.05 |
|  | rs7247977 | 0.05 | 0.05 | 0.05 | 0.05 | 0.05 | 0.05 |
|  | rs7475348 | 0.05 | 0.05 | 0.05 | 0.05 | 0.05 | 0.05 |
|  | rs75174967 | 0.05 | 0.05 | 0.05 | 0.05 | 0.05 | 0.05 |
|  | rs75530000 | 0.05 | 0.05 | 0.05 | 0.05 | 0.05 | 0.05 |
|  | rs75834729 | 0.05 | 0.05 | 0.05 | 0.05 | 0.05 | 0.05 |
|  | rs7714709 | 0.05 | 0.05 | 0.05 | 0.05 | 0.05 | 0.05 |
|  | rs848302 | 0.05 | 0.05 | 0.05 | 0.05 | 0.05 | 0.05 |
|  | rs881858 | 0.05 | 0.05 | 0.05 | 0.05 | 0.05 | 0.05 |
|  | rs898696 | 0.05 | 0.05 | 0.05 | 0.05 | 0.05 | 0.05 |
|  | rs9272117 | 0.05 | 0.05 | 0.05 | 0.05 | 0.05 | 0.05 |
|  | rs963837 | 0.05 | 0.05 | 0.05 | 0.05 | 0.05 | 0.05 |
|  | rs9887774 | 0.05 | 0.05 | 0.05 | 0.05 | 0.05 | 0.05 |
|  | rs9895661 | 0.05 | 0.05 | 0.05 | 0.05 | 0.05 | 0.05 |
| T2D | rs115001946 | 0.05 | 0.05 | 0.05 | 0.05 | 0.05 | 0.05 |
|  | rs118185025 | 0.05 | 0.05 | 0.05 | 0.05 | 0.05 | 0.05 |
|  | rs1196593 | 0.05 | 0.05 | 0.05 | 0.05 | 0.05 | 0.05 |
|  | rs1207881 | 0.05 | 0.05 | 0.05 | 0.05 | 0.05 | 0.05 |
|  | rs12199742 | 0.05 | 0.05 | 0.05 | 0.05 | 0.05 | 0.05 |
|  | rs1220583 | 0.05 | 0.05 | 0.05 | 0.05 | 0.05 | 0.05 |
|  | rs12245132 | 0.05 | 0.05 | 0.05 | 0.05 | 0.05 | 0.05 |
|  | rs12246765 | 0.05 | 0.05 | 0.05 | 0.05 | 0.05 | 0.05 |
|  | rs12267476 | 0.05 | 0.05 | 0.05 | 0.05 | 0.05 | 0.05 |
|  | rs12281632 | 0.05 | 0.05 | 0.05 | 0.05 | 0.05 | 0.05 |
|  | rs12288444 | 0.05 | 0.05 | 0.05 | 0.05 | 0.05 | 0.05 |
|  | rs12289262 | 0.05 | 0.05 | 0.05 | 0.05 | 0.05 | 0.05 |
|  | rs12289775 | 0.05 | 0.05 | 0.05 | 0.05 | 0.05 | 0.05 |
|  | rs12307894 | 0.05 | 0.05 | 0.05 | 0.05 | 0.05 | 0.05 |
|  | rs12309269 | 0.05 | 0.05 | 0.05 | 0.05 | 0.05 | 0.05 |
|  | rs12310737 | 0.05 | 0.05 | 0.05 | 0.05 | 0.05 | 0.05 |
|  | rs12312203 | 0.05 | 0.05 | 0.05 | 0.05 | 0.05 | 0.05 |
|  | rs12328738 | 0.05 | 0.05 | 0.05 | 0.05 | 0.05 | 0.05 |
|  | rs12332447 | 0.05 | 0.05 | 0.05 | 0.05 | 0.05 | 0.05 |
|  | rs1243617 | 0.05 | 0.05 | 0.05 | 0.05 | 0.05 | 0.05 |
|  | rs1252028 | 0.05 | 0.05 | 0.05 | 0.05 | 0.05 | 0.05 |
|  | rs1257311 | 0.05 | 0.05 | 0.05 | 0.05 | 0.05 | 0.05 |
|  | rs1286227 | 0.05 | 0.05 | 0.05 | 0.05 | 0.05 | 0.05 |
|  | rs1288126 | 0.05 | 0.05 | 0.05 | 0.05 | 0.05 | 0.05 |
|  | rs1288491 | 0.05 | 0.05 | 0.05 | 0.05 | 0.05 | 0.05 |
|  | rs1289447 | 0.05 | 0.05 | 0.05 | 0.05 | 0.05 | 0.05 |
|  | rs1289673 | 0.05 | 0.05 | 0.05 | 0.05 | 0.05 | 0.05 |
|  | rs1290788 | 0.05 | 0.05 | 0.05 | 0.05 | 0.05 | 0.05 |
|  | rs1291058 | 0.05 | 0.05 | 0.05 | 0.05 | 0.05 | 0.05 |
|  | rs1294228 | 0.05 | 0.05 | 0.05 | 0.05 | 0.05 | 0.05 |
|  | rs1297380 | 0.05 | 0.05 | 0.05 | 0.05 | 0.05 | 0.05 |
|  | rs1300258 | 0.05 | 0.05 | 0.05 | 0.05 | 0.05 | 0.05 |
|  | rs1313566 | 0.05 | 0.05 | 0.05 | 0.05 | 0.05 | 0.05 |
|  | rs1317192 | 0.05 | 0.05 | 0.05 | 0.05 | 0.05 | 0.05 |
|  | rs1323789 | 0.05 | 0.05 | 0.05 | 0.05 | 0.05 | 0.05 |
|  | rs1324793 | 0.05 | 0.05 | 0.05 | 0.05 | 0.05 | 0.05 |
|  | rs1324993 | 0.05 | 0.05 | 0.05 | 0.05 | 0.05 | 0.05 |
|  | rs1330759 | 0.05 | 0.05 | 0.05 | 0.05 | 0.05 | 0.05 |
|  | rs1333987 | 0.05 | 0.05 | 0.05 | 0.05 | 0.05 | 0.05 |
|  | rs1342521 | 0.05 | 0.05 | 0.05 | 0.05 | 0.05 | 0.05 |
|  | rs1343405 | 0.05 | 0.05 | 0.05 | 0.05 | 0.05 | 0.05 |
|  | rs1345956 | 0.05 | 0.05 | 0.05 | 0.05 | 0.05 | 0.05 |
|  | rs1346796 | 0.05 | 0.05 | 0.05 | 0.05 | 0.05 | 0.05 |
|  | rs1351509 | 0.05 | 0.05 | 0.05 | 0.05 | 0.05 | 0.05 |
|  | rs1357051 | 0.05 | 0.05 | 0.05 | 0.05 | 0.05 | 0.05 |
|  | rs1359604 | 0.05 | 0.05 | 0.05 | 0.05 | 0.05 | 0.05 |
|  | rs1365858 | 0.05 | 0.05 | 0.05 | 0.05 | 0.05 | 0.05 |
|  | rs1368342 | 0.05 | 0.05 | 0.05 | 0.05 | 0.05 | 0.05 |
|  | rs1373551 | 0.05 | 0.05 | 0.05 | 0.05 | 0.05 | 0.05 |
|  | rs1380699 | 0.05 | 0.05 | 0.05 | 0.05 | 0.05 | 0.05 |
|  | rs1381798 | 0.05 | 0.05 | 0.05 | 0.05 | 0.05 | 0.05 |
|  | rs1388657 | 0.05 | 0.05 | 0.05 | 0.05 | 0.05 | 0.05 |
|  | rs1403479 | 0.05 | 0.05 | 0.05 | 0.05 | 0.05 | 0.05 |
|  | rs1404415 | 0.05 | 0.05 | 0.05 | 0.05 | 0.05 | 0.05 |
|  | rs1412172 | 0.05 | 0.05 | 0.05 | 0.05 | 0.05 | 0.05 |
|  | rs1415035 | 0.05 | 0.05 | 0.05 | 0.05 | 0.05 | 0.05 |
|  | rs17126254 | 0.05 | 0.05 | 0.05 | 0.05 | 0.05 | 0.05 |
|  | rs17406632 | 0.05 | 0.05 | 0.05 | 0.05 | 0.05 | 0.05 |
|  | rs17408630 | 0.05 | 0.05 | 0.05 | 0.05 | 0.05 | 0.05 |
|  | rs17412764 | 0.05 | 0.05 | 0.05 | 0.05 | 0.05 | 0.05 |
|  | rs17420537 | 0.05 | 0.05 | 0.05 | 0.05 | 0.05 | 0.05 |
|  | rs1782586 | 0.05 | 0.05 | 0.05 | 0.05 | 0.05 | 0.05 |
|  | rs1784403 | 0.05 | 0.05 | 0.05 | 0.05 | 0.05 | 0.05 |
|  | rs1785791 | 0.05 | 0.05 | 0.05 | 0.05 | 0.05 | 0.05 |
|  | rs1786624 | 0.05 | 0.05 | 0.05 | 0.05 | 0.05 | 0.05 |
|  | rs185526062 | 0.05 | 0.05 | 0.05 | 0.05 | 0.05 | 0.05 |
|  | rs2193597 | 0.05 | 0.05 | 0.05 | 0.05 | 0.05 | 0.05 |
|  | rs2195538 | 0.05 | 0.05 | 0.05 | 0.05 | 0.05 | 0.05 |
|  | rs2203154 | 0.05 | 0.05 | 0.05 | 0.05 | 0.05 | 0.05 |
|  | rs2215089 | 0.05 | 0.05 | 0.05 | 0.05 | 0.05 | 0.05 |
|  | rs2236091 | 0.05 | 0.05 | 0.05 | 0.05 | 0.05 | 0.05 |
|  | rs2247371 | 0.05 | 0.05 | 0.05 | 0.05 | 0.05 | 0.05 |
|  | rs2269604 | 0.05 | 0.05 | 0.05 | 0.05 | 0.05 | 0.05 |
|  | rs2270241 | 0.05 | 0.05 | 0.05 | 0.05 | 0.05 | 0.05 |
|  | rs2625152 | 0.05 | 0.05 | 0.05 | 0.05 | 0.05 | 0.05 |
|  | rs2634124 | 0.05 | 0.05 | 0.05 | 0.05 | 0.05 | 0.05 |
|  | rs2642037 | 0.05 | 0.05 | 0.05 | 0.05 | 0.05 | 0.05 |
|  | rs2648726 | 0.05 | 0.05 | 0.05 | 0.05 | 0.05 | 0.05 |
|  | rs2660580 | 0.05 | 0.05 | 0.05 | 0.05 | 0.05 | 0.05 |
|  | rs2665936 | 0.05 | 0.05 | 0.05 | 0.05 | 0.05 | 0.05 |
|  | rs2680815 | 0.05 | 0.05 | 0.05 | 0.05 | 0.05 | 0.05 |
|  | rs2685820 | 0.05 | 0.05 | 0.05 | 0.05 | 0.05 | 0.05 |
|  | rs2696397 | 0.05 | 0.05 | 0.05 | 0.05 | 0.05 | 0.05 |
|  | rs2711018 | 0.05 | 0.05 | 0.05 | 0.05 | 0.05 | 0.05 |
|  | rs2720835 | 0.05 | 0.05 | 0.05 | 0.05 | 0.05 | 0.05 |
|  | rs2726233 | 0.05 | 0.05 | 0.05 | 0.05 | 0.05 | 0.05 |
|  | rs2729340 | 0.05 | 0.05 | 0.05 | 0.05 | 0.05 | 0.05 |
|  | rs2729947 | 0.05 | 0.05 | 0.05 | 0.05 | 0.05 | 0.05 |
|  | rs2732971 | 0.05 | 0.05 | 0.05 | 0.05 | 0.05 | 0.05 |
|  | rs2825871 | 0.05 | 0.05 | 0.05 | 0.05 | 0.05 | 0.05 |
|  | rs2834461 | 0.05 | 0.05 | 0.05 | 0.05 | 0.05 | 0.05 |
|  | rs2836085 | 0.05 | 0.05 | 0.05 | 0.05 | 0.05 | 0.05 |
|  | rs2851113 | 0.05 | 0.05 | 0.05 | 0.05 | 0.05 | 0.05 |
|  | rs2868868 | 0.05 | 0.05 | 0.05 | 0.05 | 0.05 | 0.05 |
|  | rs41403944 | 0.05 | 0.05 | 0.05 | 0.05 | 0.05 | 0.05 |
|  | rs41507350 | 0.05 | 0.05 | 0.05 | 0.05 | 0.05 | 0.05 |
|  | rs4268029 | 0.05 | 0.05 | 0.05 | 0.05 | 0.05 | 0.05 |
|  | rs55809138 | 0.05 | 0.05 | 0.05 | 0.05 | 0.05 | 0.05 |
|  | rs57759769 | 0.05 | 0.05 | 0.05 | 0.05 | 0.05 | 0.05 |
|  | rs57782738 | 0.05 | 0.05 | 0.05 | 0.05 | 0.05 | 0.05 |
|  | rs57869750 | 0.05 | 0.05 | 0.05 | 0.05 | 0.05 | 0.05 |
|  | rs57909385 | 0.05 | 0.05 | 0.05 | 0.05 | 0.05 | 0.05 |
|  | rs610603 | 0.05 | 0.05 | 0.05 | 0.05 | 0.05 | 0.05 |
|  | rs622723 | 0.05 | 0.05 | 0.05 | 0.05 | 0.05 | 0.05 |
|  | rs62362195 | 0.05 | 0.05 | 0.05 | 0.05 | 0.05 | 0.05 |
|  | rs62388784 | 0.05 | 0.05 | 0.05 | 0.05 | 0.05 | 0.05 |
|  | rs62390553 | 0.05 | 0.05 | 0.05 | 0.05 | 0.05 | 0.05 |
|  | rs623976 | 0.05 | 0.05 | 0.05 | 0.05 | 0.05 | 0.05 |
|  | rs62399564 | 0.05 | 0.05 | 0.05 | 0.05 | 0.05 | 0.05 |
|  | rs628504 | 0.05 | 0.05 | 0.05 | 0.05 | 0.05 | 0.05 |
|  | rs629510 | 0.05 | 0.05 | 0.05 | 0.05 | 0.05 | 0.05 |
|  | rs629601 | 0.05 | 0.05 | 0.05 | 0.05 | 0.05 | 0.05 |
|  | rs629831 | 0.05 | 0.05 | 0.05 | 0.05 | 0.05 | 0.05 |
|  | rs629959 | 0.05 | 0.05 | 0.05 | 0.05 | 0.05 | 0.05 |
|  | rs630782 | 0.05 | 0.05 | 0.05 | 0.05 | 0.05 | 0.05 |
|  | rs630788 | 0.05 | 0.05 | 0.05 | 0.05 | 0.05 | 0.05 |
|  | rs631462 | 0.05 | 0.05 | 0.05 | 0.05 | 0.05 | 0.05 |
|  | rs632028 | 0.05 | 0.05 | 0.05 | 0.05 | 0.05 | 0.05 |
|  | rs632348 | 0.05 | 0.05 | 0.05 | 0.05 | 0.05 | 0.05 |
|  | rs634905 | 0.05 | 0.05 | 0.05 | 0.05 | 0.05 | 0.05 |
|  | rs638144 | 0.05 | 0.05 | 0.05 | 0.05 | 0.05 | 0.05 |
|  | rs638560 | 0.05 | 0.05 | 0.05 | 0.05 | 0.05 | 0.05 |
|  | rs639900 | 0.05 | 0.05 | 0.05 | 0.05 | 0.05 | 0.05 |
|  | rs640701 | 0.05 | 0.05 | 0.05 | 0.05 | 0.05 | 0.05 |
|  | rs642253 | 0.05 | 0.05 | 0.05 | 0.05 | 0.05 | 0.05 |
|  | rs642846 | 0.05 | 0.05 | 0.05 | 0.05 | 0.05 | 0.05 |
|  | rs644953 | 0.05 | 0.05 | 0.05 | 0.05 | 0.05 | 0.05 |
|  | rs646767 | 0.05 | 0.05 | 0.05 | 0.05 | 0.05 | 0.05 |
|  | rs647577 | 0.05 | 0.05 | 0.05 | 0.05 | 0.05 | 0.05 |
|  | rs647861 | 0.05 | 0.05 | 0.05 | 0.05 | 0.05 | 0.05 |
|  | rs650974 | 0.05 | 0.05 | 0.05 | 0.05 | 0.05 | 0.05 |
|  | rs651030 | 0.05 | 0.05 | 0.05 | 0.05 | 0.05 | 0.05 |
|  | rs651407 | 0.05 | 0.05 | 0.05 | 0.05 | 0.05 | 0.05 |
|  | rs651430 | 0.05 | 0.05 | 0.05 | 0.05 | 0.05 | 0.05 |
|  | rs652247 | 0.05 | 0.05 | 0.05 | 0.05 | 0.05 | 0.05 |
|  | rs653575 | 0.05 | 0.05 | 0.05 | 0.05 | 0.05 | 0.05 |
|  | rs6943989 | 0.05 | 0.05 | 0.05 | 0.05 | 0.05 | 0.05 |
|  | rs6946330 | 0.05 | 0.05 | 0.05 | 0.05 | 0.05 | 0.05 |
|  | rs6950894 | 0.05 | 0.05 | 0.05 | 0.05 | 0.05 | 0.05 |
|  | rs6953558 | 0.05 | 0.05 | 0.05 | 0.05 | 0.05 | 0.05 |
|  | rs6961433 | 0.05 | 0.05 | 0.05 | 0.05 | 0.05 | 0.05 |
|  | rs6962630 | 0.05 | 0.05 | 0.05 | 0.05 | 0.05 | 0.05 |
|  | rs75851293 | 0.05 | 0.05 | 0.05 | 0.05 | 0.05 | 0.05 |
|  | rs77375735 | 0.05 | 0.05 | 0.05 | 0.05 | 0.05 | 0.05 |
|  | rs77381959 | 0.05 | 0.05 | 0.05 | 0.05 | 0.05 | 0.05 |
|  | rs77585820 | 0.05 | 0.05 | 0.05 | 0.05 | 0.05 | 0.05 |
|  | rs77635234 | 0.05 | 0.05 | 0.05 | 0.05 | 0.05 | 0.05 |
|  | rs77646504 | 0.05 | 0.05 | 0.05 | 0.05 | 0.05 | 0.05 |
|  | rs77828219 | 0.05 | 0.05 | 0.05 | 0.05 | 0.05 | 0.05 |
|  | rs77828969 | 0.05 | 0.05 | 0.05 | 0.05 | 0.05 | 0.05 |
|  | rs77873412 | 0.05 | 0.05 | 0.05 | 0.05 | 0.05 | 0.05 |
| T2D (adjBMI) | rs1029919 | 0.05 | 0.05 | 0.05 | 0.05 | 0.05 | 0.05 |
|  | rs1033958 | 0.05 | 0.05 | 0.05 | 0.05 | 0.05 | 0.05 |
|  | rs1033970 | 0.05 | 0.05 | 0.05 | 0.05 | 0.05 | 0.05 |
|  | rs1036193 | 0.05 | 0.05 | 0.05 | 0.05 | 0.05 | 0.05 |
|  | rs115001946 | 0.05 | 0.05 | 0.05 | 0.05 | 0.05 | 0.05 |
|  | rs118184783 | 0.05 | 0.05 | 0.05 | 0.05 | 0.05 | 0.05 |
|  | rs118185025 | 0.05 | 0.05 | 0.05 | 0.05 | 0.05 | 0.05 |
|  | rs1196593 | 0.05 | 0.05 | 0.05 | 0.05 | 0.05 | 0.05 |
|  | rs1207881 | 0.05 | 0.05 | 0.05 | 0.05 | 0.05 | 0.05 |
|  | rs1218796 | 0.05 | 0.05 | 0.05 | 0.05 | 0.05 | 0.05 |
|  | rs12198019 | 0.05 | 0.05 | 0.05 | 0.05 | 0.05 | 0.05 |
|  | rs12199742 | 0.05 | 0.05 | 0.05 | 0.05 | 0.05 | 0.05 |
|  | rs12245132 | 0.05 | 0.05 | 0.05 | 0.05 | 0.05 | 0.05 |
|  | rs12246765 | 0.05 | 0.05 | 0.05 | 0.05 | 0.05 | 0.05 |
|  | rs12267476 | 0.05 | 0.05 | 0.05 | 0.05 | 0.05 | 0.05 |
|  | rs12281632 | 0.05 | 0.05 | 0.05 | 0.05 | 0.05 | 0.05 |
|  | rs12288444 | 0.05 | 0.05 | 0.05 | 0.05 | 0.05 | 0.05 |
|  | rs12289262 | 0.05 | 0.05 | 0.05 | 0.05 | 0.05 | 0.05 |
|  | rs12289775 | 0.05 | 0.05 | 0.05 | 0.05 | 0.05 | 0.05 |
|  | rs12307894 | 0.05 | 0.05 | 0.05 | 0.05 | 0.05 | 0.05 |
|  | rs12309269 | 0.05 | 0.05 | 0.05 | 0.05 | 0.05 | 0.05 |
|  | rs12310737 | 0.05 | 0.05 | 0.05 | 0.05 | 0.05 | 0.05 |
|  | rs12312203 | 0.05 | 0.05 | 0.05 | 0.05 | 0.05 | 0.05 |
|  | rs12320114 | 0.05 | 0.05 | 0.05 | 0.05 | 0.05 | 0.05 |
|  | rs12328738 | 0.05 | 0.05 | 0.05 | 0.05 | 0.05 | 0.05 |
|  | rs12332447 | 0.05 | 0.05 | 0.05 | 0.05 | 0.05 | 0.05 |
|  | rs1238521 | 0.05 | 0.05 | 0.05 | 0.05 | 0.05 | 0.05 |
|  | rs1243617 | 0.05 | 0.05 | 0.05 | 0.05 | 0.05 | 0.05 |
|  | rs1248051 | 0.05 | 0.05 | 0.05 | 0.05 | 0.05 | 0.05 |
|  | rs1252028 | 0.05 | 0.05 | 0.05 | 0.05 | 0.05 | 0.05 |
|  | rs1257311 | 0.05 | 0.05 | 0.05 | 0.05 | 0.05 | 0.05 |
|  | rs1286227 | 0.05 | 0.05 | 0.05 | 0.05 | 0.05 | 0.05 |
|  | rs1288126 | 0.05 | 0.05 | 0.05 | 0.05 | 0.05 | 0.05 |
|  | rs1288491 | 0.05 | 0.05 | 0.05 | 0.05 | 0.05 | 0.05 |
|  | rs1289447 | 0.05 | 0.05 | 0.05 | 0.05 | 0.05 | 0.05 |
|  | rs1289673 | 0.05 | 0.05 | 0.05 | 0.05 | 0.05 | 0.05 |
|  | rs1290788 | 0.05 | 0.05 | 0.05 | 0.05 | 0.05 | 0.05 |
|  | rs1291058 | 0.05 | 0.05 | 0.05 | 0.05 | 0.05 | 0.05 |
|  | rs1294228 | 0.05 | 0.05 | 0.05 | 0.05 | 0.05 | 0.05 |
|  | rs1297380 | 0.05 | 0.05 | 0.05 | 0.05 | 0.05 | 0.05 |
|  | rs1300258 | 0.05 | 0.05 | 0.05 | 0.05 | 0.05 | 0.05 |
|  | rs1313566 | 0.05 | 0.05 | 0.05 | 0.05 | 0.05 | 0.05 |
|  | rs1317192 | 0.05 | 0.05 | 0.05 | 0.05 | 0.05 | 0.05 |
|  | rs1323789 | 0.05 | 0.05 | 0.05 | 0.05 | 0.05 | 0.05 |
|  | rs1324793 | 0.05 | 0.05 | 0.05 | 0.05 | 0.05 | 0.05 |
|  | rs1324993 | 0.05 | 0.05 | 0.05 | 0.05 | 0.05 | 0.05 |
|  | rs1330759 | 0.05 | 0.05 | 0.05 | 0.05 | 0.05 | 0.05 |
|  | rs1333987 | 0.05 | 0.05 | 0.05 | 0.05 | 0.05 | 0.05 |
|  | rs1342521 | 0.05 | 0.05 | 0.05 | 0.05 | 0.05 | 0.05 |
|  | rs1343405 | 0.05 | 0.05 | 0.05 | 0.05 | 0.05 | 0.05 |
|  | rs1345956 | 0.05 | 0.05 | 0.05 | 0.05 | 0.05 | 0.05 |
|  | rs1346796 | 0.05 | 0.05 | 0.05 | 0.05 | 0.05 | 0.05 |
|  | rs1351509 | 0.05 | 0.05 | 0.05 | 0.05 | 0.05 | 0.05 |
|  | rs1351562 | 0.05 | 0.05 | 0.05 | 0.05 | 0.05 | 0.05 |
|  | rs1357051 | 0.05 | 0.05 | 0.05 | 0.05 | 0.05 | 0.05 |
|  | rs1359604 | 0.05 | 0.05 | 0.05 | 0.05 | 0.05 | 0.05 |
|  | rs1365858 | 0.05 | 0.05 | 0.05 | 0.05 | 0.05 | 0.05 |
|  | rs1368342 | 0.05 | 0.05 | 0.05 | 0.05 | 0.05 | 0.05 |
|  | rs1373551 | 0.05 | 0.05 | 0.05 | 0.05 | 0.05 | 0.05 |
|  | rs1380699 | 0.05 | 0.05 | 0.05 | 0.05 | 0.05 | 0.05 |
|  | rs1381798 | 0.05 | 0.05 | 0.05 | 0.05 | 0.05 | 0.05 |
|  | rs1388657 | 0.05 | 0.05 | 0.05 | 0.05 | 0.05 | 0.05 |
|  | rs1394016 | 0.05 | 0.05 | 0.05 | 0.05 | 0.05 | 0.05 |
|  | rs1403479 | 0.05 | 0.05 | 0.05 | 0.05 | 0.05 | 0.05 |
|  | rs1404415 | 0.05 | 0.05 | 0.05 | 0.05 | 0.05 | 0.05 |
|  | rs1409302 | 0.05 | 0.05 | 0.05 | 0.05 | 0.05 | 0.05 |
|  | rs1412172 | 0.05 | 0.05 | 0.05 | 0.05 | 0.05 | 0.05 |
|  | rs1415035 | 0.05 | 0.05 | 0.05 | 0.05 | 0.05 | 0.05 |
|  | rs1415961 | 0.05 | 0.05 | 0.05 | 0.05 | 0.05 | 0.05 |
|  | rs17316029 | 0.05 | 0.05 | 0.05 | 0.05 | 0.05 | 0.05 |
|  | rs17406632 | 0.05 | 0.05 | 0.05 | 0.05 | 0.05 | 0.05 |
|  | rs17408630 | 0.05 | 0.05 | 0.05 | 0.05 | 0.05 | 0.05 |
|  | rs17412764 | 0.05 | 0.05 | 0.05 | 0.05 | 0.05 | 0.05 |
|  | rs17420537 | 0.05 | 0.05 | 0.05 | 0.05 | 0.05 | 0.05 |
|  | rs1784403 | 0.05 | 0.05 | 0.05 | 0.05 | 0.05 | 0.05 |
|  | rs1785791 | 0.05 | 0.05 | 0.05 | 0.05 | 0.05 | 0.05 |
|  | rs1786624 | 0.05 | 0.05 | 0.05 | 0.05 | 0.05 | 0.05 |
|  | rs185526062 | 0.05 | 0.05 | 0.05 | 0.05 | 0.05 | 0.05 |
|  | rs2193597 | 0.05 | 0.05 | 0.05 | 0.05 | 0.05 | 0.05 |
|  | rs2195538 | 0.05 | 0.05 | 0.05 | 0.05 | 0.05 | 0.05 |
|  | rs2195981 | 0.05 | 0.05 | 0.05 | 0.05 | 0.05 | 0.05 |
|  | rs2209967 | 0.05 | 0.05 | 0.05 | 0.05 | 0.05 | 0.05 |
|  | rs2210407 | 0.05 | 0.05 | 0.05 | 0.05 | 0.05 | 0.05 |
|  | rs2215089 | 0.05 | 0.05 | 0.05 | 0.05 | 0.05 | 0.05 |
|  | rs2236091 | 0.05 | 0.05 | 0.05 | 0.05 | 0.05 | 0.05 |
|  | rs2247371 | 0.05 | 0.05 | 0.05 | 0.05 | 0.05 | 0.05 |
|  | rs2625152 | 0.05 | 0.05 | 0.05 | 0.05 | 0.05 | 0.05 |
|  | rs2629162 | 0.05 | 0.05 | 0.05 | 0.05 | 0.05 | 0.05 |
|  | rs2634124 | 0.05 | 0.05 | 0.05 | 0.05 | 0.05 | 0.05 |
|  | rs2642037 | 0.05 | 0.05 | 0.05 | 0.05 | 0.05 | 0.05 |
|  | rs2648726 | 0.05 | 0.05 | 0.05 | 0.05 | 0.05 | 0.05 |
|  | rs2660580 | 0.05 | 0.05 | 0.05 | 0.05 | 0.05 | 0.05 |
|  | rs2665936 | 0.05 | 0.05 | 0.05 | 0.05 | 0.05 | 0.05 |
|  | rs2685820 | 0.05 | 0.05 | 0.05 | 0.05 | 0.05 | 0.05 |
|  | rs2696397 | 0.05 | 0.05 | 0.05 | 0.05 | 0.05 | 0.05 |
|  | rs2706879 | 0.05 | 0.05 | 0.05 | 0.05 | 0.05 | 0.05 |
|  | rs2711018 | 0.05 | 0.05 | 0.05 | 0.05 | 0.05 | 0.05 |
|  | rs2711870 | 0.05 | 0.05 | 0.05 | 0.05 | 0.05 | 0.05 |
|  | rs2718011 | 0.05 | 0.05 | 0.05 | 0.05 | 0.05 | 0.05 |
|  | rs2720835 | 0.05 | 0.05 | 0.05 | 0.05 | 0.05 | 0.05 |
|  | rs2726233 | 0.05 | 0.05 | 0.05 | 0.05 | 0.05 | 0.05 |
|  | rs2729340 | 0.05 | 0.05 | 0.05 | 0.05 | 0.05 | 0.05 |
|  | rs2729947 | 0.05 | 0.05 | 0.05 | 0.05 | 0.05 | 0.05 |
|  | rs2732971 | 0.05 | 0.05 | 0.05 | 0.05 | 0.05 | 0.05 |
|  | rs2757684 | 0.05 | 0.05 | 0.05 | 0.05 | 0.05 | 0.05 |
|  | rs2825871 | 0.05 | 0.05 | 0.05 | 0.05 | 0.05 | 0.05 |
|  | rs2834461 | 0.05 | 0.05 | 0.05 | 0.05 | 0.05 | 0.05 |
|  | rs2836085 | 0.05 | 0.05 | 0.05 | 0.05 | 0.05 | 0.05 |
|  | rs2850443 | 0.05 | 0.05 | 0.05 | 0.05 | 0.05 | 0.05 |
|  | rs2851113 | 0.05 | 0.05 | 0.06 | 0.06 | 0.06 | 0.05 |
|  | rs2868868 | 0.05 | 0.05 | 0.05 | 0.05 | 0.05 | 0.05 |
|  | rs2910621 | 0.05 | 0.05 | 0.05 | 0.05 | 0.05 | 0.05 |
|  | rs34197105 | 0.05 | 0.05 | 0.05 | 0.05 | 0.05 | 0.05 |
|  | rs34204285 | 0.05 | 0.05 | 0.05 | 0.05 | 0.05 | 0.05 |
|  | rs41507350 | 0.05 | 0.05 | 0.05 | 0.05 | 0.05 | 0.05 |
|  | rs55809138 | 0.05 | 0.05 | 0.05 | 0.05 | 0.05 | 0.05 |
|  | rs62388784 | 0.05 | 0.05 | 0.05 | 0.05 | 0.05 | 0.05 |
|  | rs62399564 | 0.05 | 0.05 | 0.05 | 0.05 | 0.05 | 0.05 |
|  | rs6943989 | 0.05 | 0.05 | 0.05 | 0.05 | 0.05 | 0.05 |
|  | rs6946330 | 0.05 | 0.05 | 0.05 | 0.05 | 0.05 | 0.05 |
|  | rs6950894 | 0.05 | 0.05 | 0.05 | 0.05 | 0.05 | 0.05 |
|  | rs6953558 | 0.05 | 0.05 | 0.05 | 0.05 | 0.05 | 0.05 |
|  | rs6962630 | 0.05 | 0.05 | 0.05 | 0.05 | 0.05 | 0.05 |
|  | rs6966326 | 0.05 | 0.05 | 0.05 | 0.05 | 0.05 | 0.05 |
|  | rs71627333 | 0.05 | 0.05 | 0.05 | 0.05 | 0.05 | 0.05 |
|  | rs75254889 | 0.05 | 0.05 | 0.05 | 0.05 | 0.05 | 0.05 |
|  | rs75851293 | 0.05 | 0.05 | 0.05 | 0.05 | 0.05 | 0.05 |
|  | rs77297139 | 0.05 | 0.05 | 0.05 | 0.05 | 0.05 | 0.05 |
|  | rs77375735 | 0.05 | 0.05 | 0.05 | 0.05 | 0.05 | 0.05 |
|  | rs77381959 | 0.05 | 0.05 | 0.05 | 0.05 | 0.05 | 0.05 |
|  | rs77646504 | 0.05 | 0.05 | 0.05 | 0.05 | 0.05 | 0.05 |
|  | rs77828219 | 0.05 | 0.05 | 0.05 | 0.05 | 0.05 | 0.05 |
|  | rs77828969 | 0.05 | 0.05 | 0.05 | 0.05 | 0.05 | 0.05 |
|  | rs77873412 | 0.05 | 0.05 | 0.05 | 0.05 | 0.05 | 0.05 |
| Metabolic syndrome | rs651821 | NA | NA | NA | NA | NA | NA |
|  | rs671 | NA | NA | NA | NA | NA | NA |
| Gout | rs1260326 | NA | NA | NA | NA | NA | NA |
|  | rs1014290 | NA | NA | NA | NA | NA | NA |
|  | rs3114020 | NA | NA | NA | NA | NA | NA |
|  | rs2285340 | NA | NA | NA | NA | NA | NA |
|  | rs4073582 | NA | NA | NA | NA | NA | NA |
|  | rs4766566 | NA | NA | NA | NA | NA | NA |
| Graves' disease | rs1061537 | 0.05 | 0.05 | 0.06 | 0.05 | 0.08 | 0.08 |
|  | rs11065783 | 0.05 | 0.05 | 0.06 | 0.05 | 0.07 | 0.07 |
|  | rs11571292 | 0.05 | 0.05 | 0.06 | 0.05 | 0.07 | 0.07 |
|  | rs117201373 | 0.05 | 0.05 | 0.06 | 0.05 | 0.07 | 0.07 |
|  | rs13136820 | 0.05 | 0.05 | 0.06 | 0.05 | 0.06 | 0.06 |
|  | rs148781980 | 0.05 | 0.05 | 0.06 | 0.05 | 0.07 | 0.07 |
|  | rs1569723 | 0.05 | 0.05 | 0.06 | 0.05 | 0.06 | 0.06 |
|  | rs2049218 | 0.05 | 0.05 | 0.06 | 0.05 | 0.06 | 0.06 |
|  | rs2456453 | 0.05 | 0.05 | 0.06 | 0.05 | 0.06 | 0.06 |
|  | rs4248153 | 0.05 | 0.05 | 0.06 | 0.05 | 0.07 | 0.07 |
|  | rs4903961 | 0.05 | 0.05 | 0.06 | 0.05 | 0.07 | 0.07 |
|  | rs9296074 | 0.05 | 0.05 | 0.08 | 0.06 | 0.10 | 0.12 |
|  | rs9319588 | 0.05 | 0.05 | 0.06 | 0.05 | 0.06 | 0.06 |

Supplementary Table 10. The SNPs related to six types of DSCs

| SNP | se | pval | beta | effect_allele | other_allele | eaf | exposure | id.exposure |
| --- | --- | --- | --- | --- | --- | --- | --- | --- |
| rs1229984 | 0.0519895 | 3.01E-44 | 0.725421 | C | T | 0.261948 | Esophageal cancer | bbj-a-117 |
| rs4646776 | 0.0465468 | 1.85E-25 | 0.485393 | C | G | 0.240533 | Esophageal cancer | bbj-a-117 |
| rs760077 | 0.0262477 | 2.07E-36 | 0.330759 | T | A | 0.849044 | Gastric cancer | bbj-a-119 |
| rs3805495 | 0.0181743 | 8.92E-20 | -0.165411 | T | C | 0.57871 | Gastric cancer | bbj-a-119 |
| rs1050437 | 0.0186533 | 1.24E-08 | -0.106218 | T | C | 0.369641 | Gastric cancer | bbj-a-119 |
| rs9368777 | 0.0290577 | 2.86E-09 | 0.172592 | C | G | 0.893154 | Gastric cancer | bbj-a-119 |
| rs2978977 | 0.0183041 | 2.78E-43 | 0.252482 | A | C | 0.493194 | Gastric cancer | bbj-a-119 |
| rs12022676 | 0.0191492 | 9.63E-11 | 0.123947 | A | G | 0.282814 | Colorectal cancer | bbj-a-107 |
| rs7722513 | 0.0201413 | 1.27E-13 | -0.14924 | G | C | 0.715325 | Colorectal cancer | bbj-a-107 |
| rs4871022 | 0.0185523 | 1.28E-24 | -0.190014 | T | C | 0.680316 | Colorectal cancer | bbj-a-107 |
| rs2437841 | 0.0174438 | 2.27E-08 | -0.0975154 | A | G | 0.459556 | Colorectal cancer | bbj-a-107 |
| rs10811654 | 0.0174328 | 4.23E-09 | -0.102417 | G | A | 0.471424 | Colorectal cancer | bbj-a-107 |
| rs704017 | 0.0181317 | 1.16E-09 | 0.110347 | G | A | 0.344962 | Colorectal cancer | bbj-a-107 |
| rs76681197 | 0.0187282 | 5.55E-10 | 0.116163 | G | A | 0.667314 | Colorectal cancer | bbj-a-107 |
| rs12241008 | 0.0198501 | 3.20E-11 | 0.131745 | C | T | 0.253869 | Colorectal cancer | bbj-a-107 |
| rs9733843 | 0.0179651 | 9.83E-15 | -0.139075 | G | A | 0.382312 | Colorectal cancer | bbj-a-107 |
| rs11066015 | 0.0199428 | 1.49E-13 | -0.147345 | A | G | 0.239107 | Colorectal cancer | bbj-a-107 |
| rs11874392 | 0.0184227 | 2.29E-20 | -0.170369 | T | A | 0.655505 | Colorectal cancer | bbj-a-107 |
| rs6140071 | 0.0216981 | 1.55E-09 | 0.131041 | T | C | 0.202251 | Colorectal cancer | bbj-a-107 |
| rs2049942 | 0.0185455 | 1.01E-09 | -0.113264 | G | A | 0.672422 | Colorectal cancer | bbj-a-107 |
| rs1131500 | 0.0338153 | 1.97E-08 | 0.189858 | T | C | 0.586876 | hepatocellular carcinoma | bbj-a-158 |
| rs113777417 | 0.0340474 | 2.39E-09 | 0.203222 | C | A | 0.589394 | hepatocellular carcinoma | bbj-a-158 |
| rs8107030 | 0.0597699 | 7.96E-10 | 0.367335 | G | A | 0.100965 | hepatocellular carcinoma | bbj-a-158 |


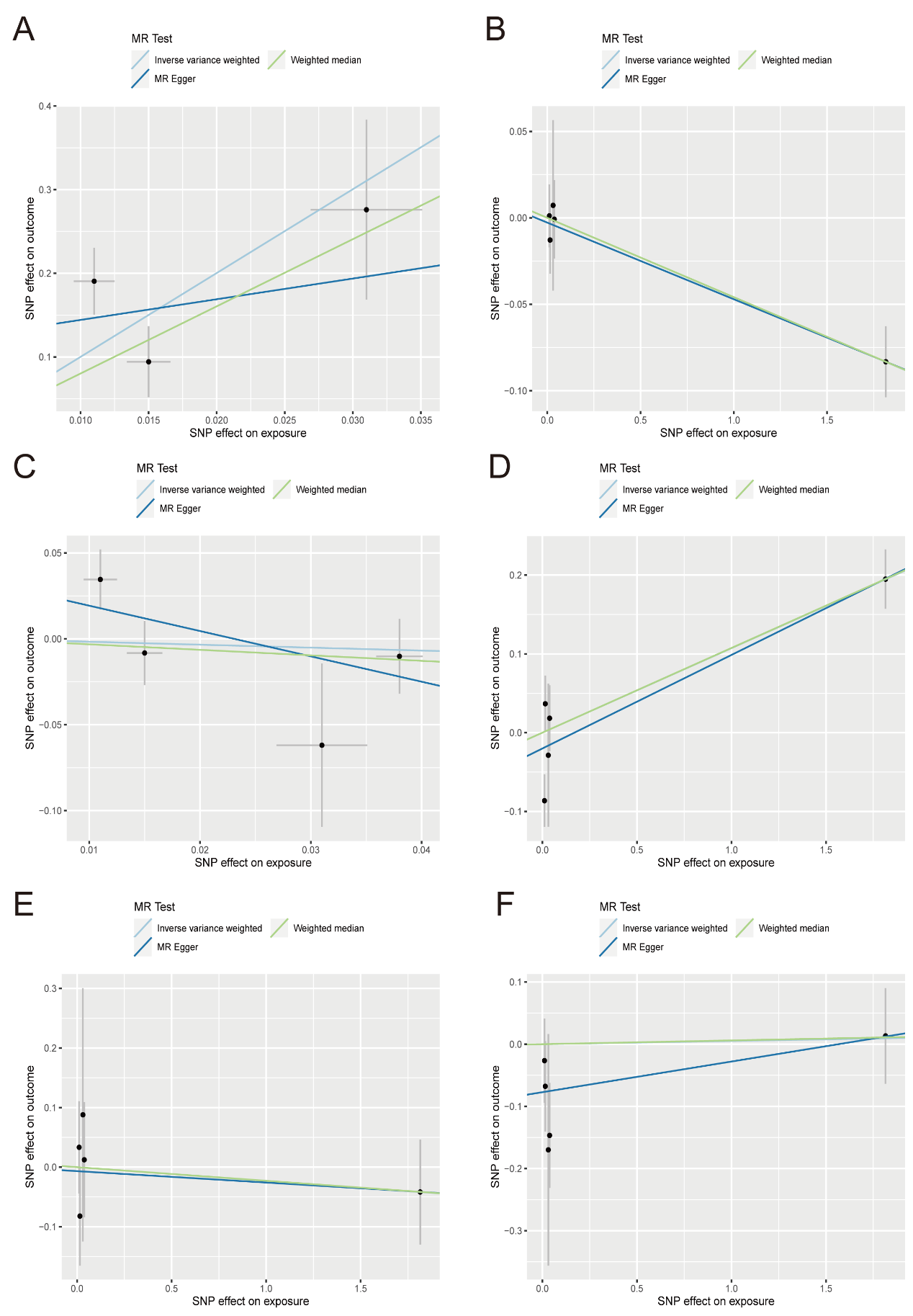


Supplementary Figure 1. Scatter plots for MR analyses of the causal effect of “Ever/never drinkers” on digestive system cancers. (A) esophageal cancer; (B) gastric cancer; (C) colorectal cancer; (D) hepatocellular carcinoma; (E) biliary tract cancer; (F) pancreatic carcinoma.


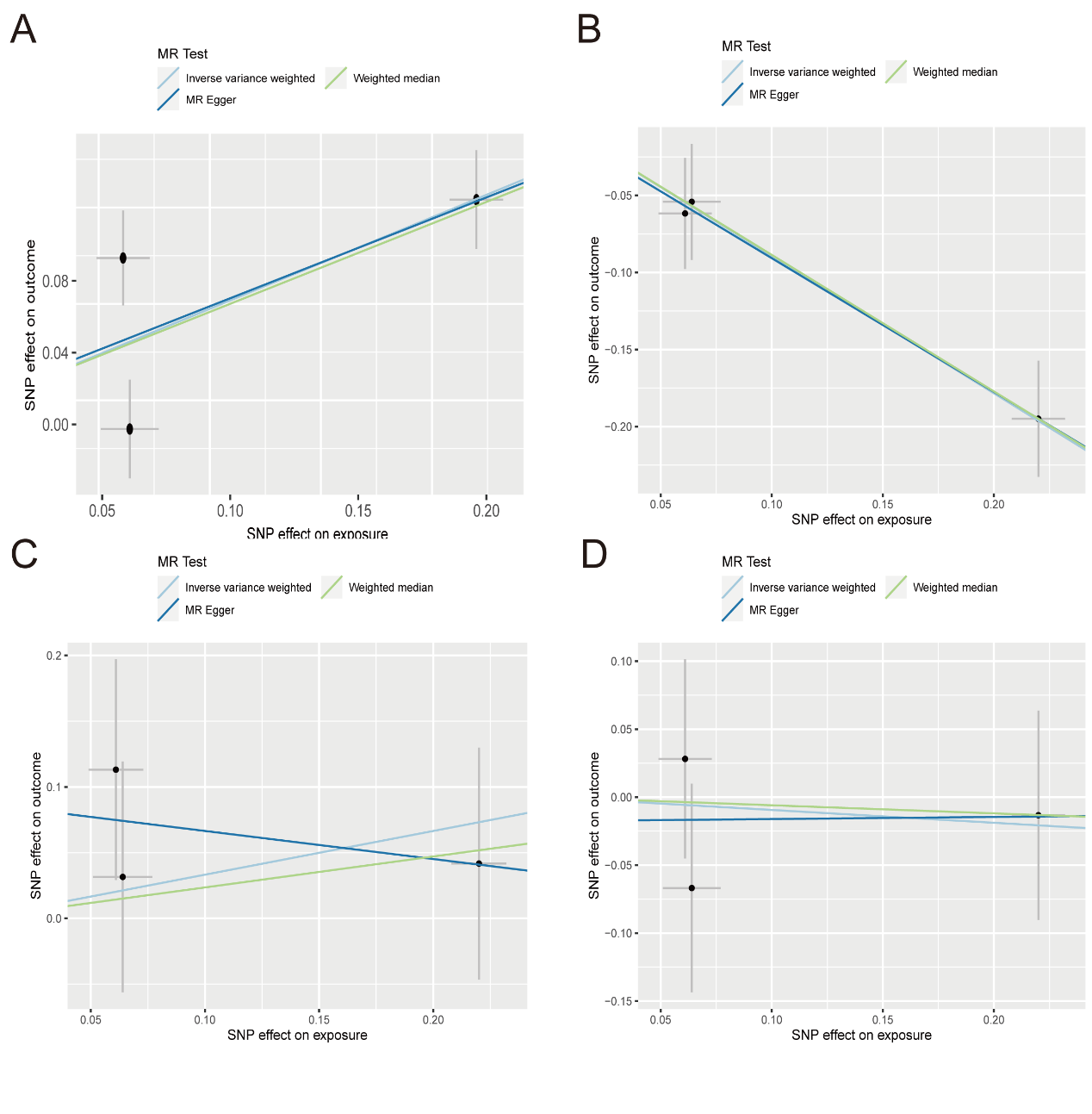


Supplementary Figure 2. Scatter plots for MR analyses of the causal effect of “Sweet taste” on digestive system cancers. (A) gastric cancer; (B) hepatocellular carcinoma; (C) biliary tract cancer; (D) pancreatic carcinoma.


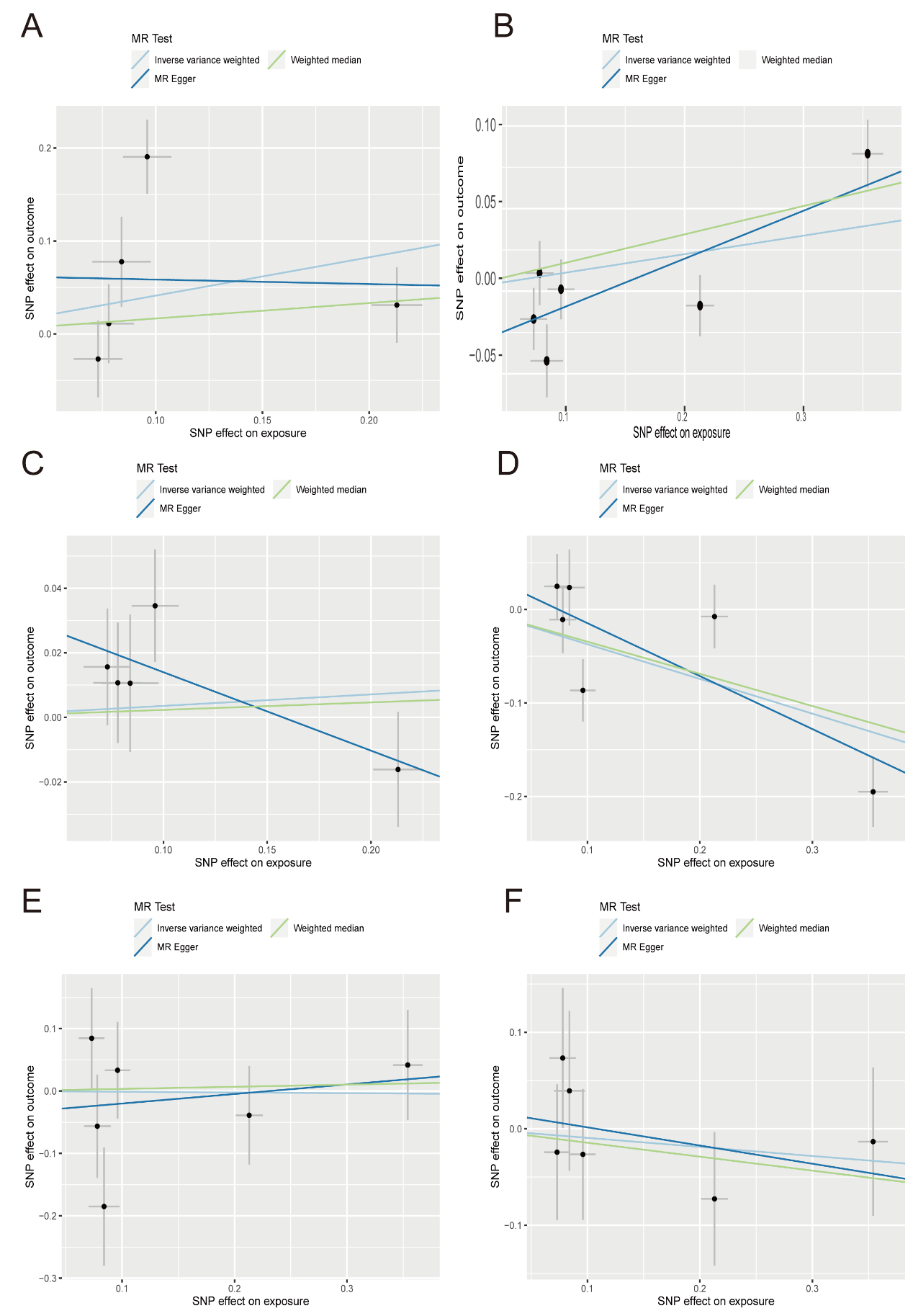


Supplementary Figure 3. Scatter plots for MR analyses of the causal effect of “Coffee consumption” on digestive system cancers. (A) esophageal cancer; (B) gastric cancer; (C) colorectal cancer; (D) hepatocellular carcinoma; (E) biliary tract cancer; (F) pancreatic carcinoma.


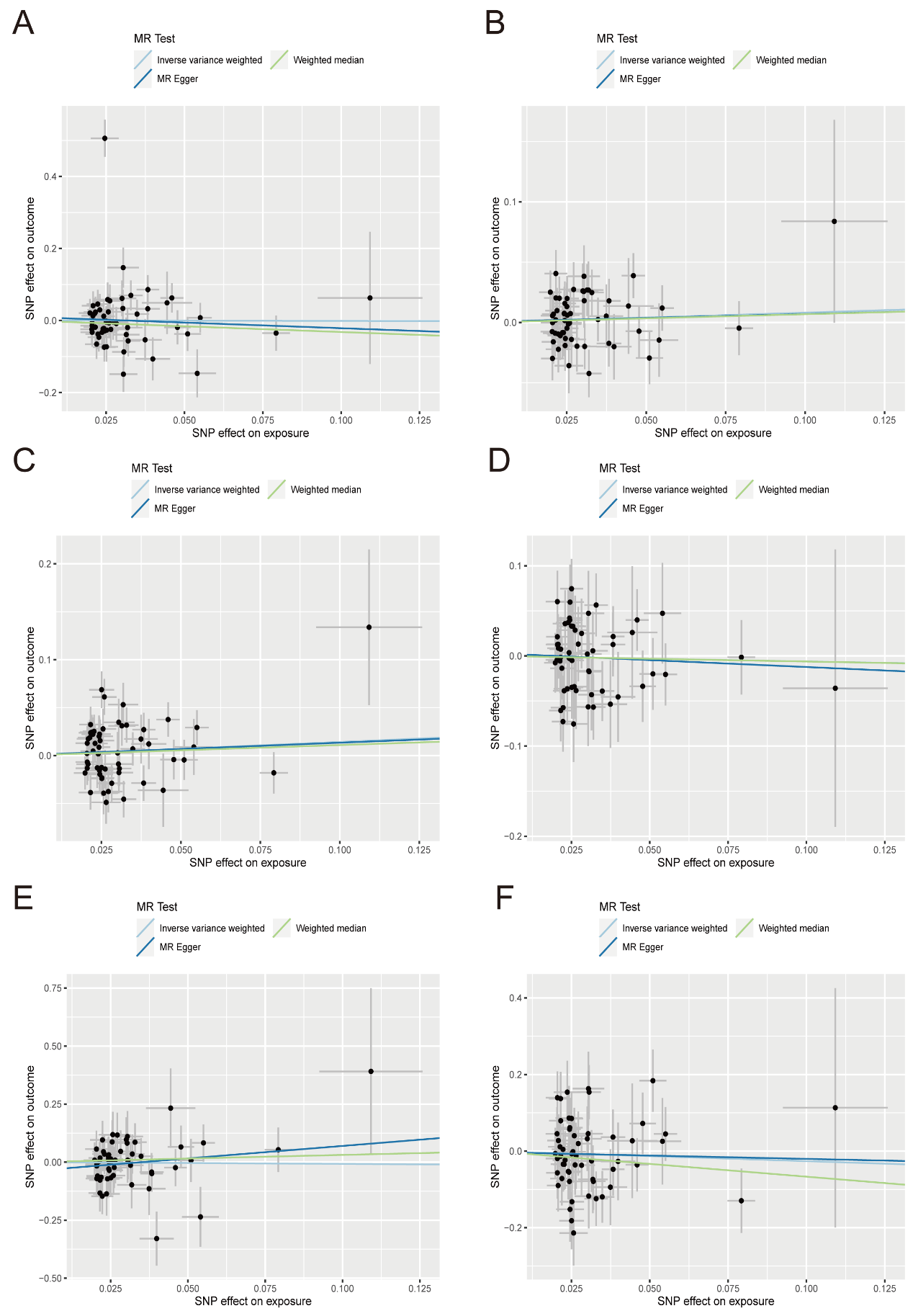


Supplementary Figure 4. Scatter plots for MR analyses of the causal effect of “BMI” on digestive system cancers. (A) esophageal cancer; (B) gastric cancer; (C) colorectal cancer; (D) hepatocellular carcinoma; (E) biliary tract cancer; (F) pancreatic carcinoma.


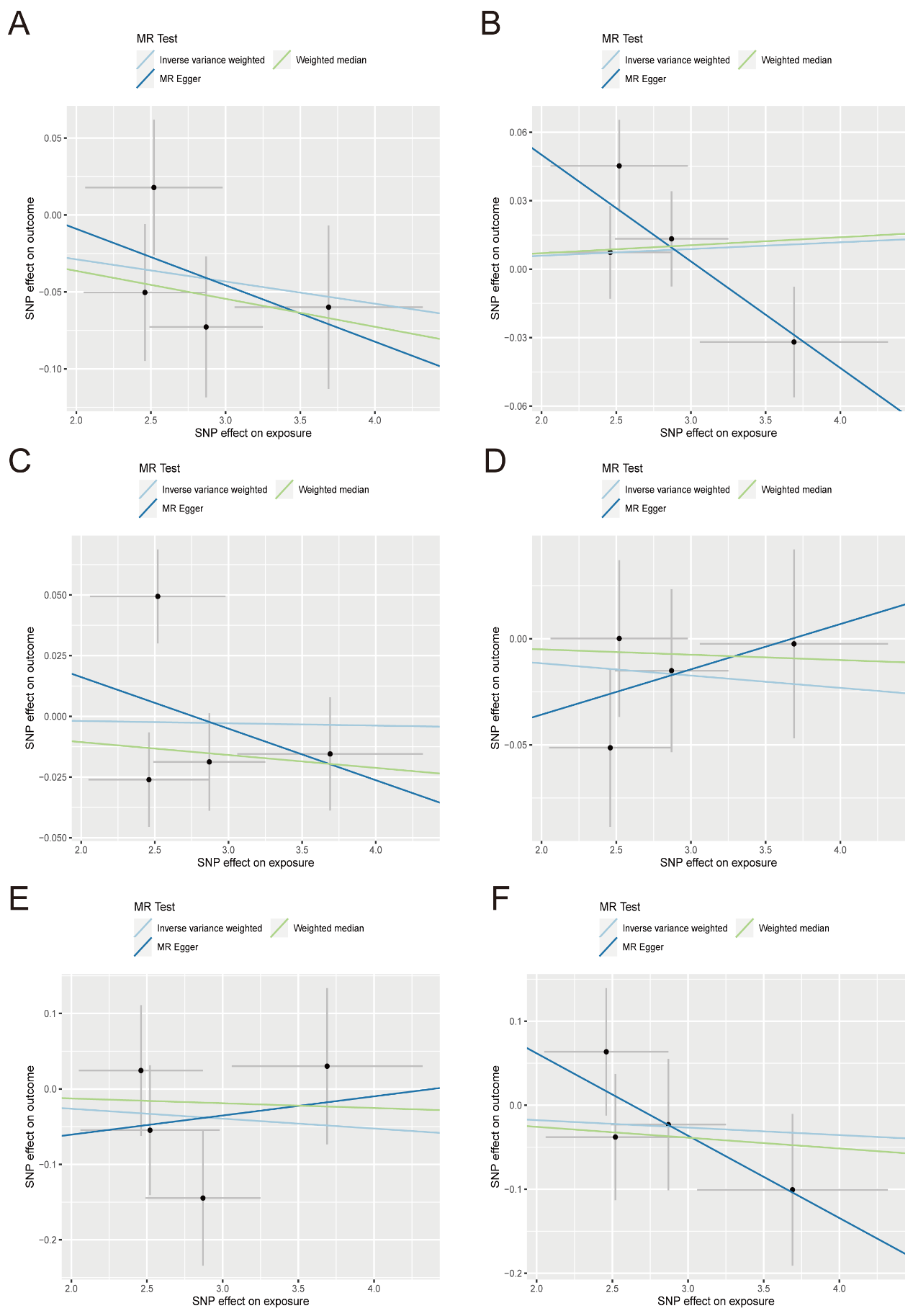


Supplementary Figure 5. Scatter plots for MR analyses of the causal effect of “Waist circumference (adjBMI)” on digestive system cancers. (A) esophageal cancer; (B) gastric cancer; (C) colorectal cancer; (D) hepatocellular carcinoma; (E) biliary tract cancer; (F) pancreatic carcinoma.


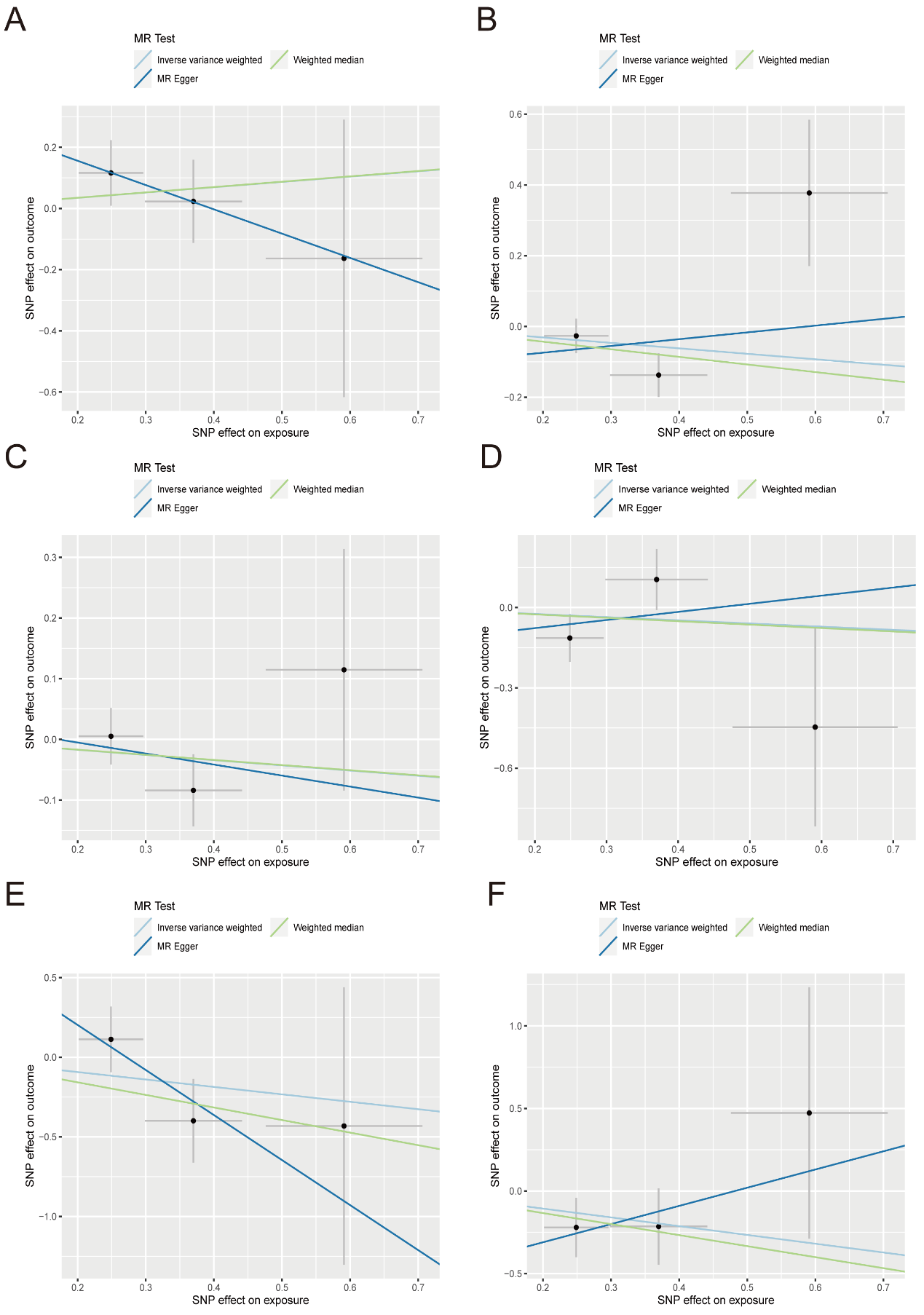


Supplementary Figure 6. Scatter plots for MR analyses of the causal effect of “Education status” on digestive system cancers. (A) esophageal cancer; (B) gastric cancer; (C) colorectal cancer; (D) hepatocellular carcinoma; (E) biliary tract cancer; (F) pancreatic carcinoma.


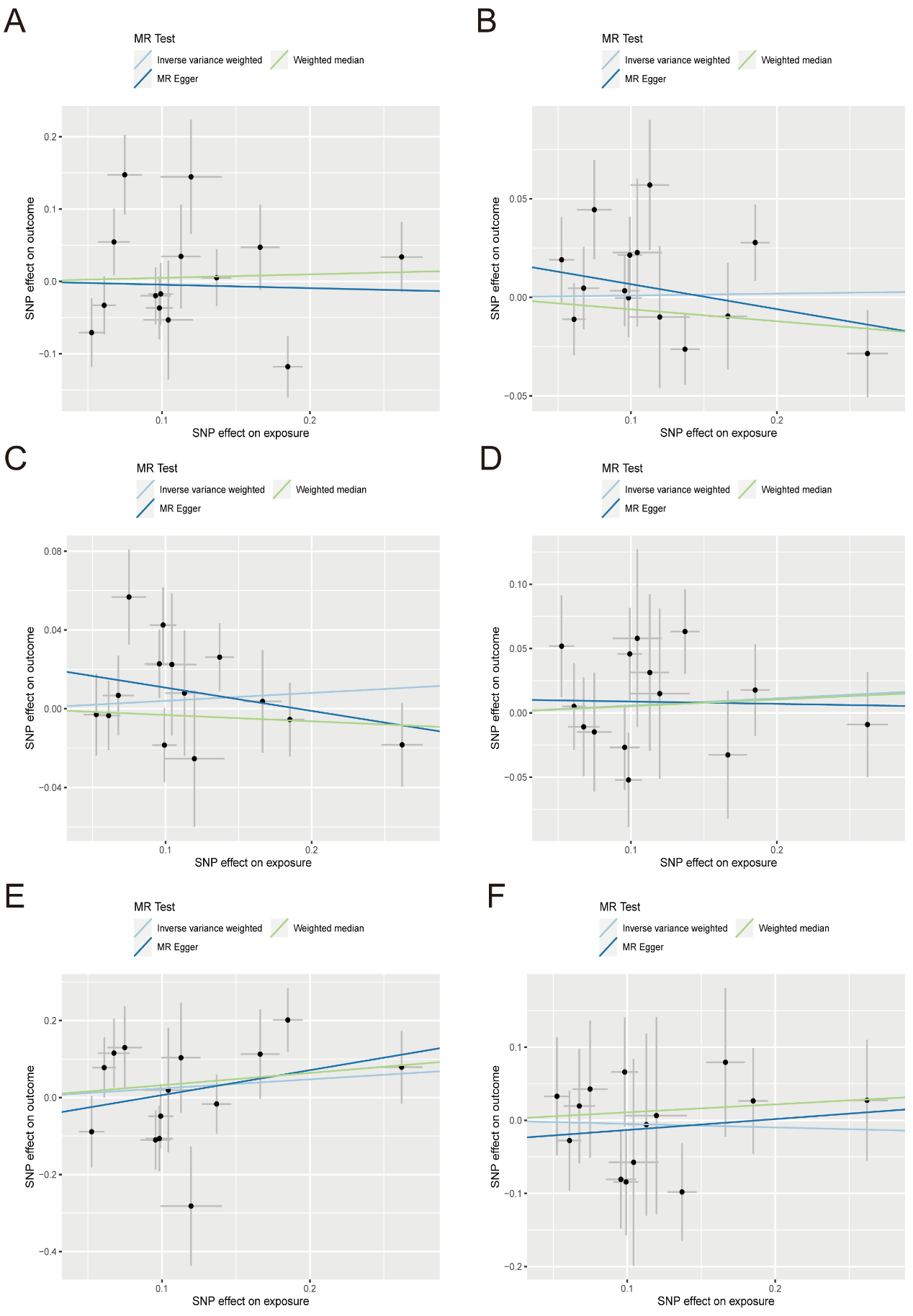


Supplementary Figure7. Scatter plots for MR analyses of the causal effect of “HDL-C” on digestive system cancers. (A) esophageal cancer; (B) gastric cancer; (C) colorectal cancer; (D) hepatocellular carcinoma; (E) biliary tract cancer; (F) pancreatic carcinoma.


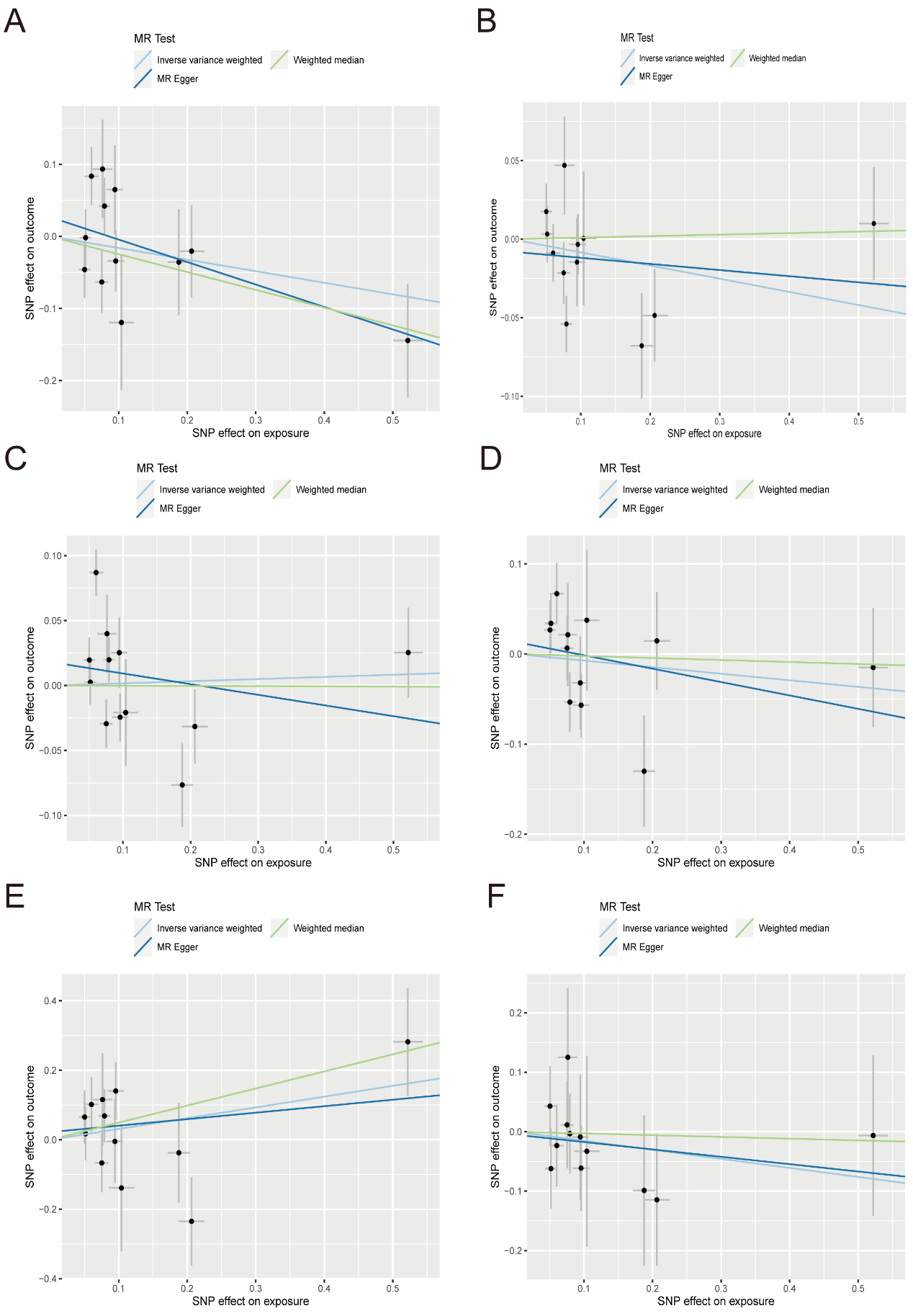


Supplementary Figure 8. Scatter plots for MR analyses of the causal effect of “LDL-C” on digestive system cancers. (A) esophageal cancer; (B) gastric cancer; (C) colorectal cancer; (D) hepatocellular carcinoma; (E) biliary tract cancer; (F) pancreatic carcinoma.


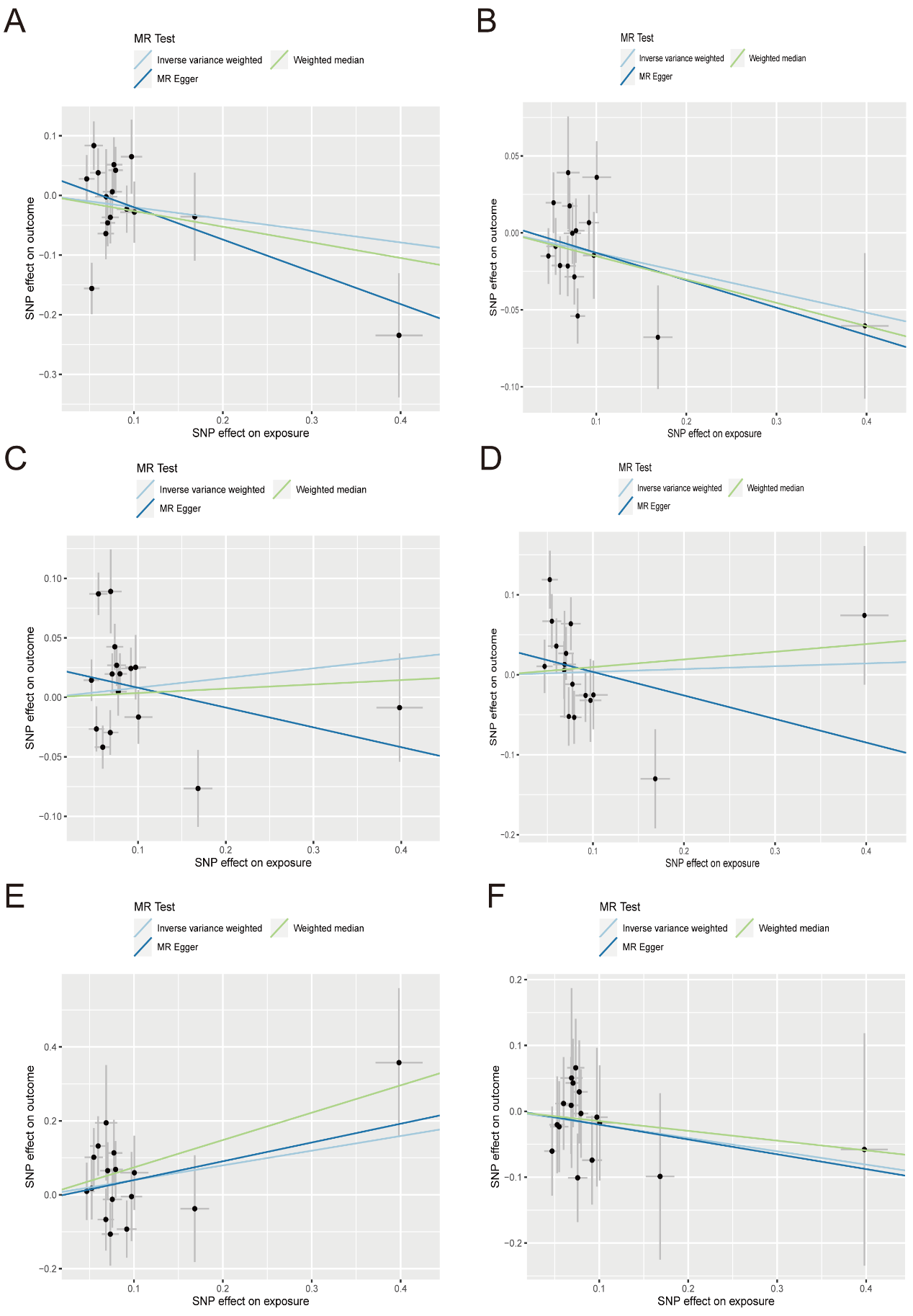


Supplementary Figure 9. Scatter plots for MR analyses of the causal effect of “TC-C” on digestive system cancers. (A) esophageal cancer; (B) gastric cancer; (C) colorectal cancer; (D) hepatocellular carcinoma; (E) biliary tract cancer; (F) pancreatic carcinoma.


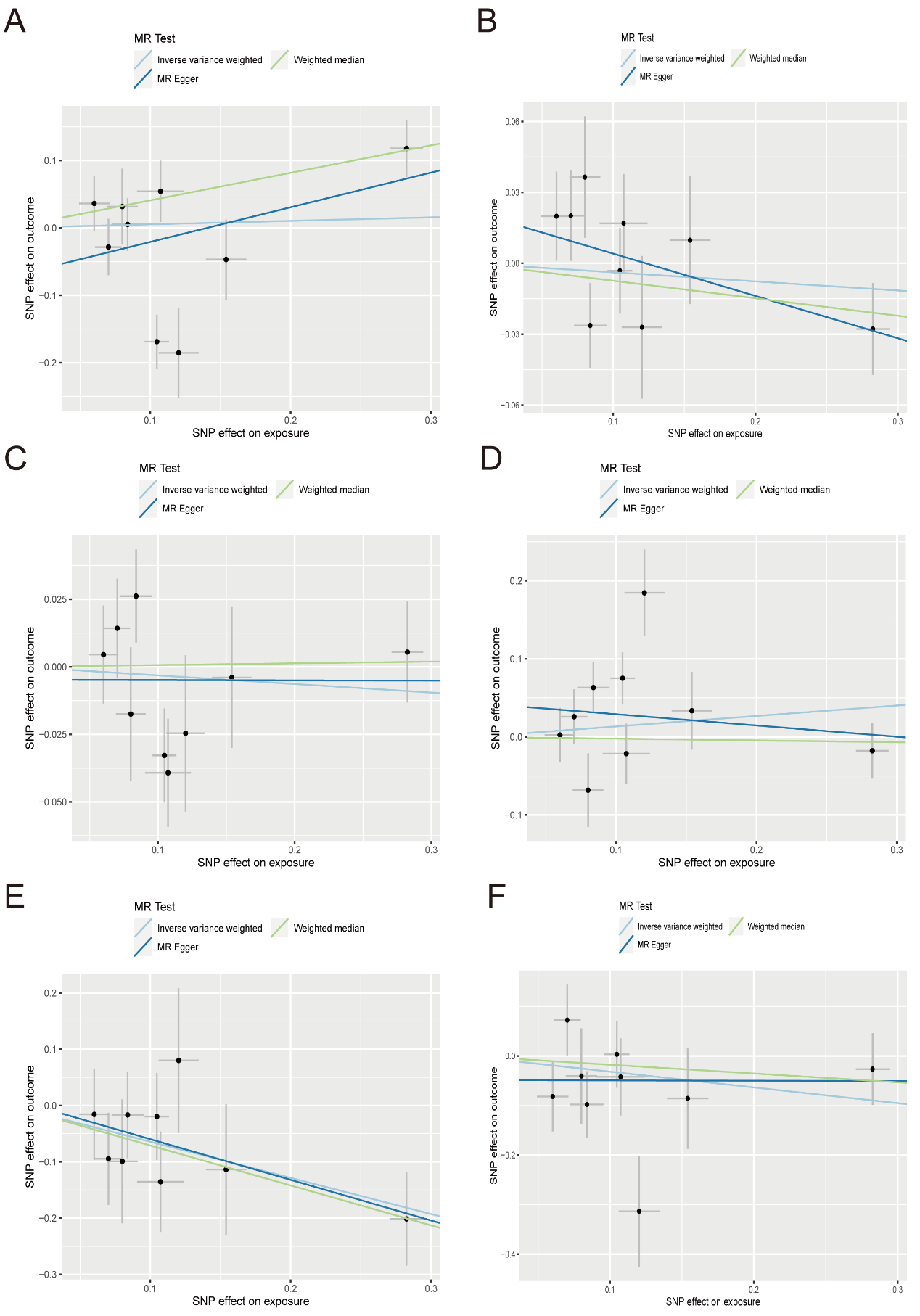


Supplementary Figure 10. Scatter plots for MR analyses of the causal effect of “TG-C” on digestive system cancers. (A) esophageal cancer; (B) gastric cancer; (C) colorectal cancer; (D) hepatocellular carcinoma; (E) biliary tract cancer; (F) pancreatic carcinoma.


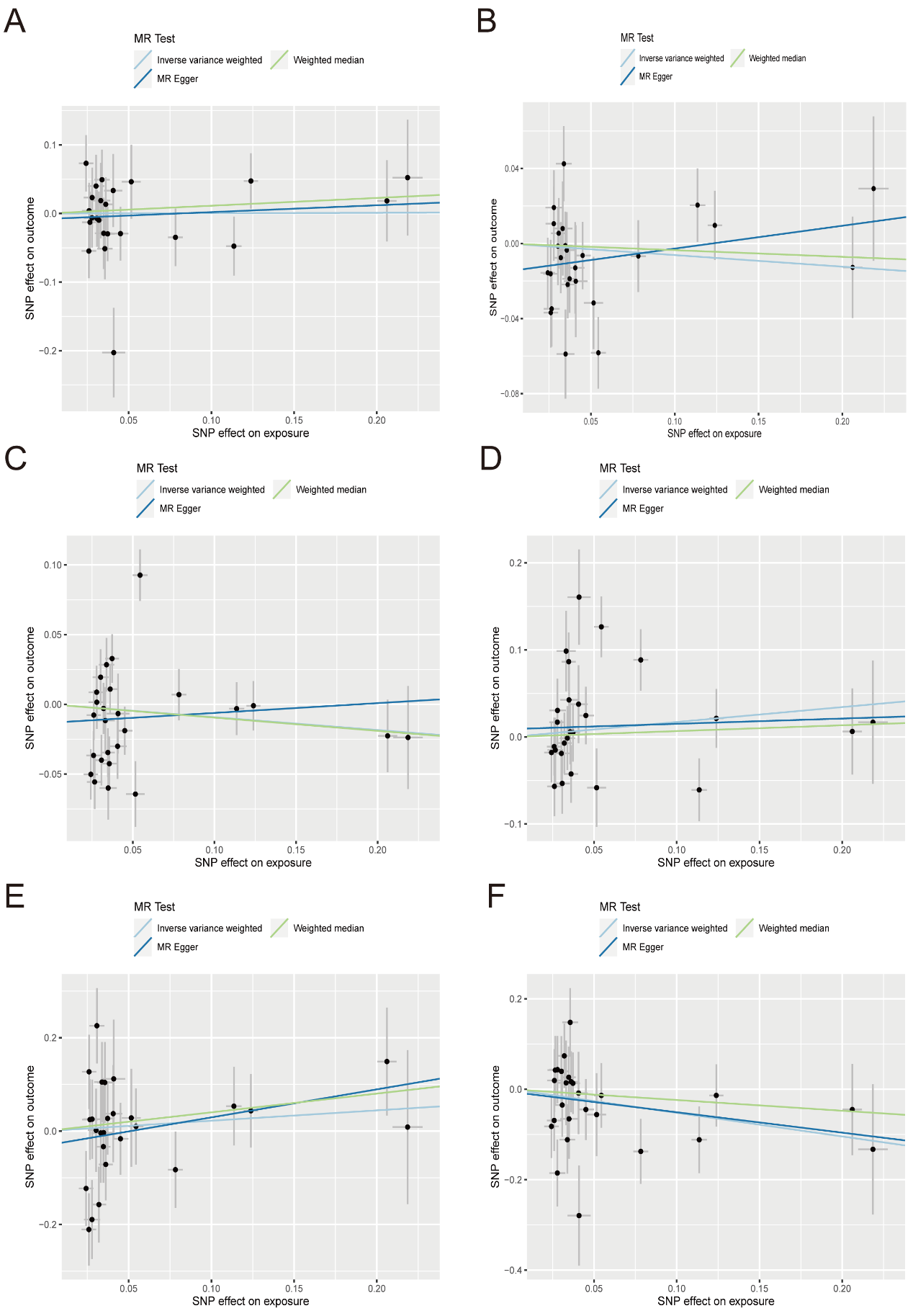


Supplementary Figure 11. Scatter plots for MR analyses of the causal effect of “Uric acid” on digestive system cancers. (A) esophageal cancer; (B) gastric cancer; (C) colorectal cancer; (D) hepatocellular carcinoma; (E) biliary tract cancer; (F) pancreatic carcinoma.


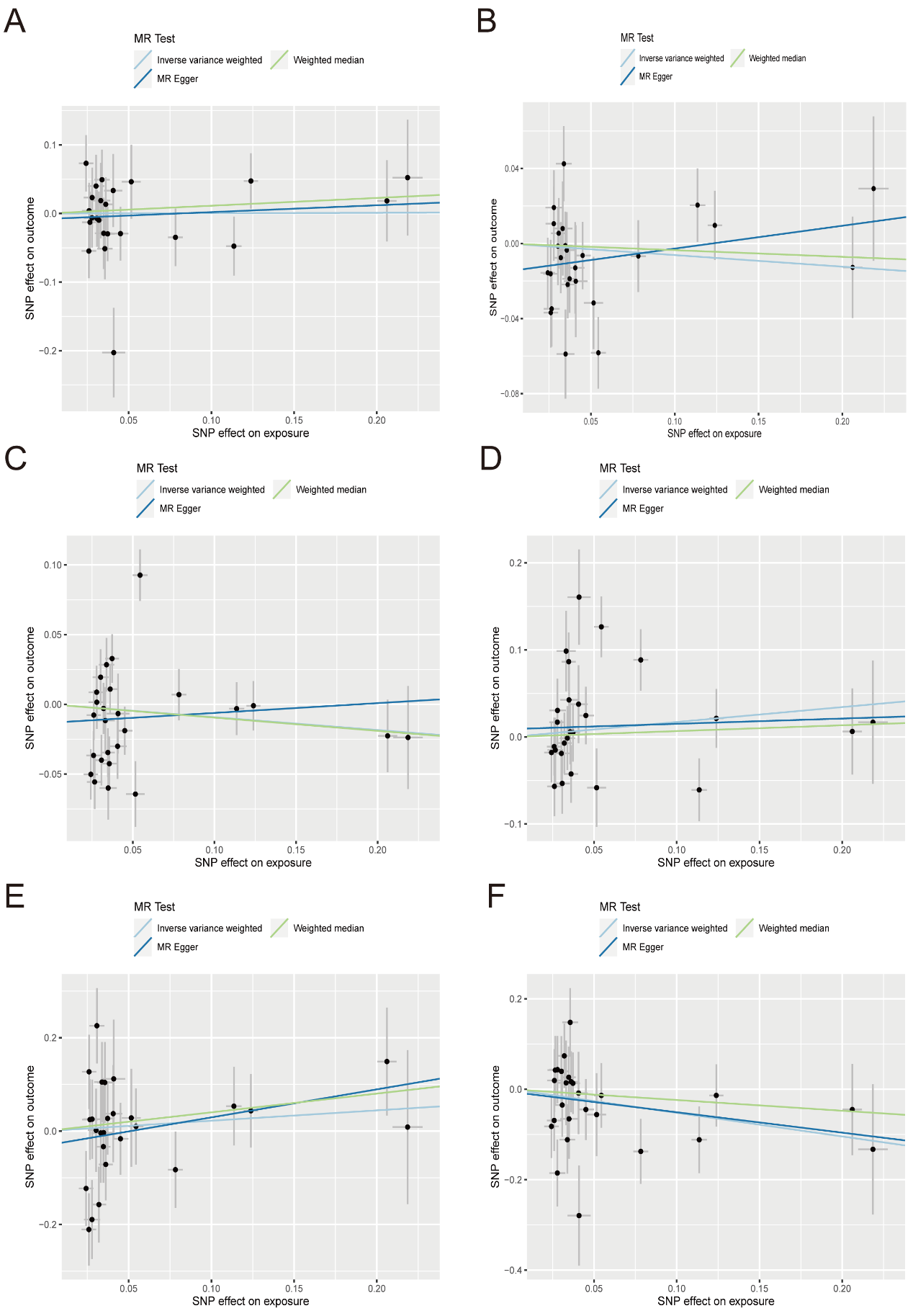


Supplementary Figure 12. Scatter plots for MR analyses of the causal effect of “Creatinine” on digestive system cancers. (A) esophageal cancer; (B) gastric cancer; (C) colorectal cancer; (D) hepatocellular carcinoma; (E) biliary tract cancer; (F) pancreatic carcinoma.


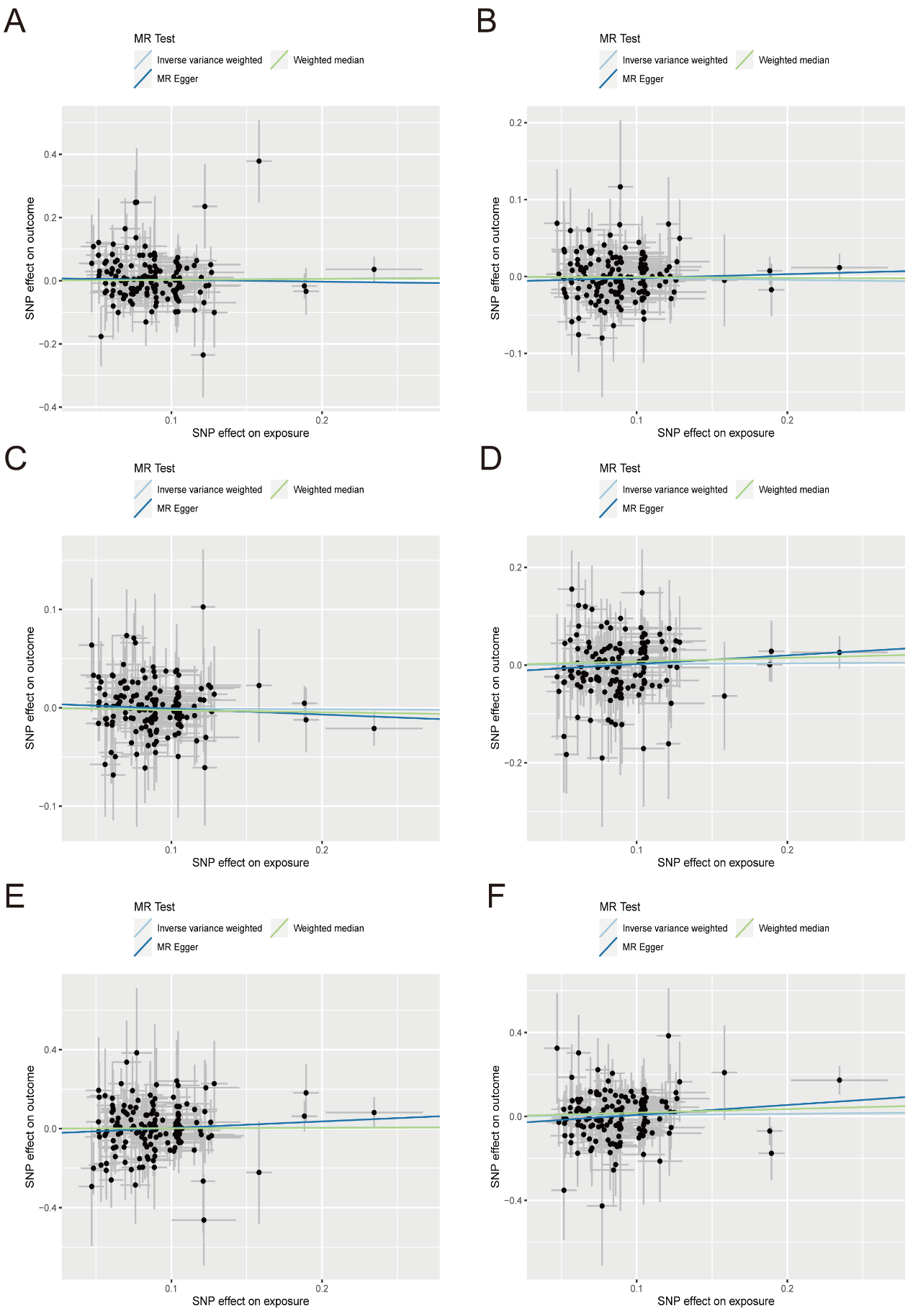


Supplementary Figure 13. Scatter plots for MR analyses of the causal effect of “T2D” on digestive system cancers. (A) esophageal cancer; (B) gastric cancer; (C) colorectal cancer; (D) hepatocellular carcinoma; (E) biliary tract cancer; (F) pancreatic carcinoma.


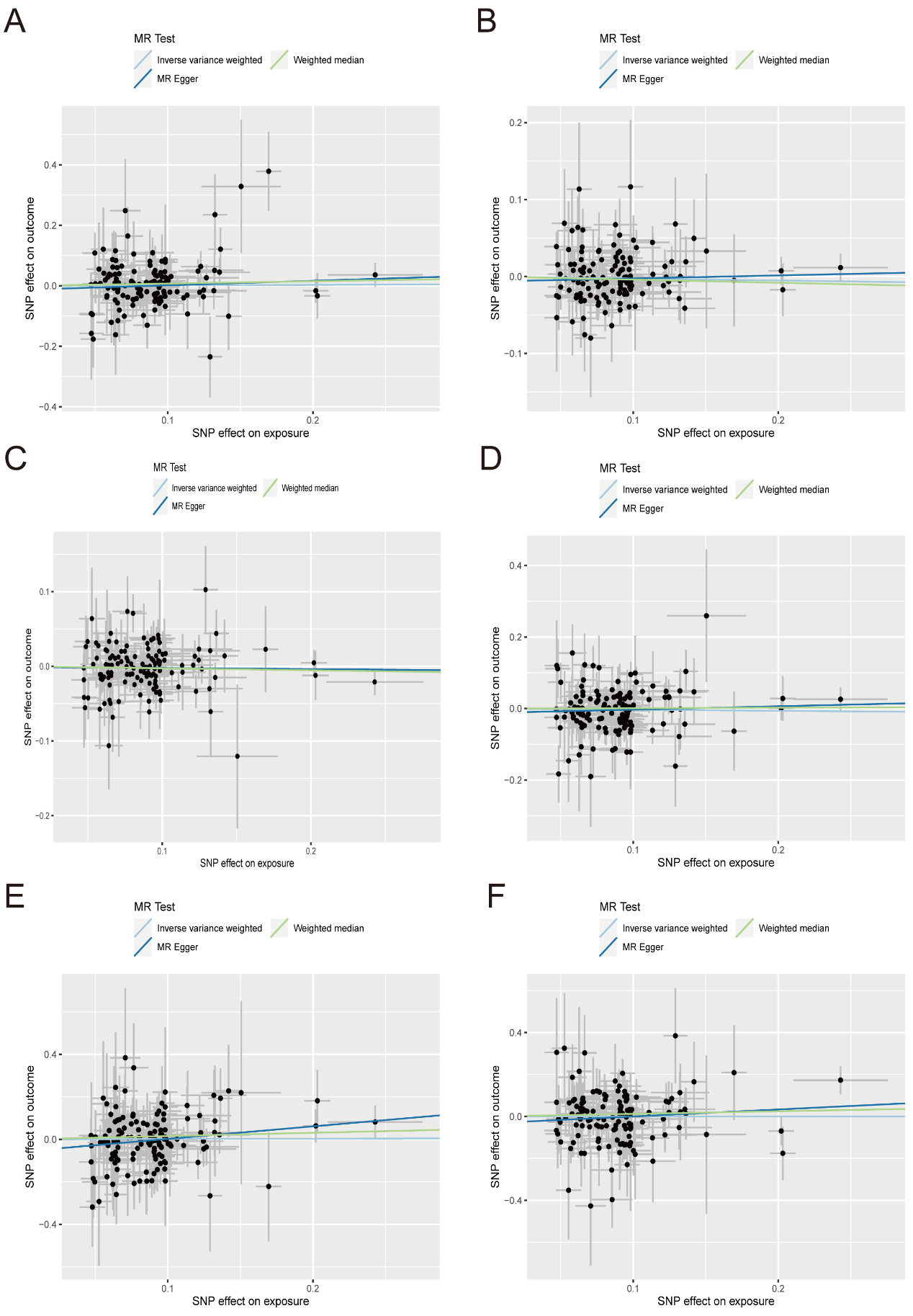


Supplementary Figure 14. Scatter plots for MR analyses of the causal effect of “T2D (adjBMI)” on digestive system cancers. (A) esophageal cancer; (B) gastric cancer; (C) colorectal cancer; (D) hepatocellular carcinoma; (E) biliary tract cancer; (F) pancreatic carcinoma.


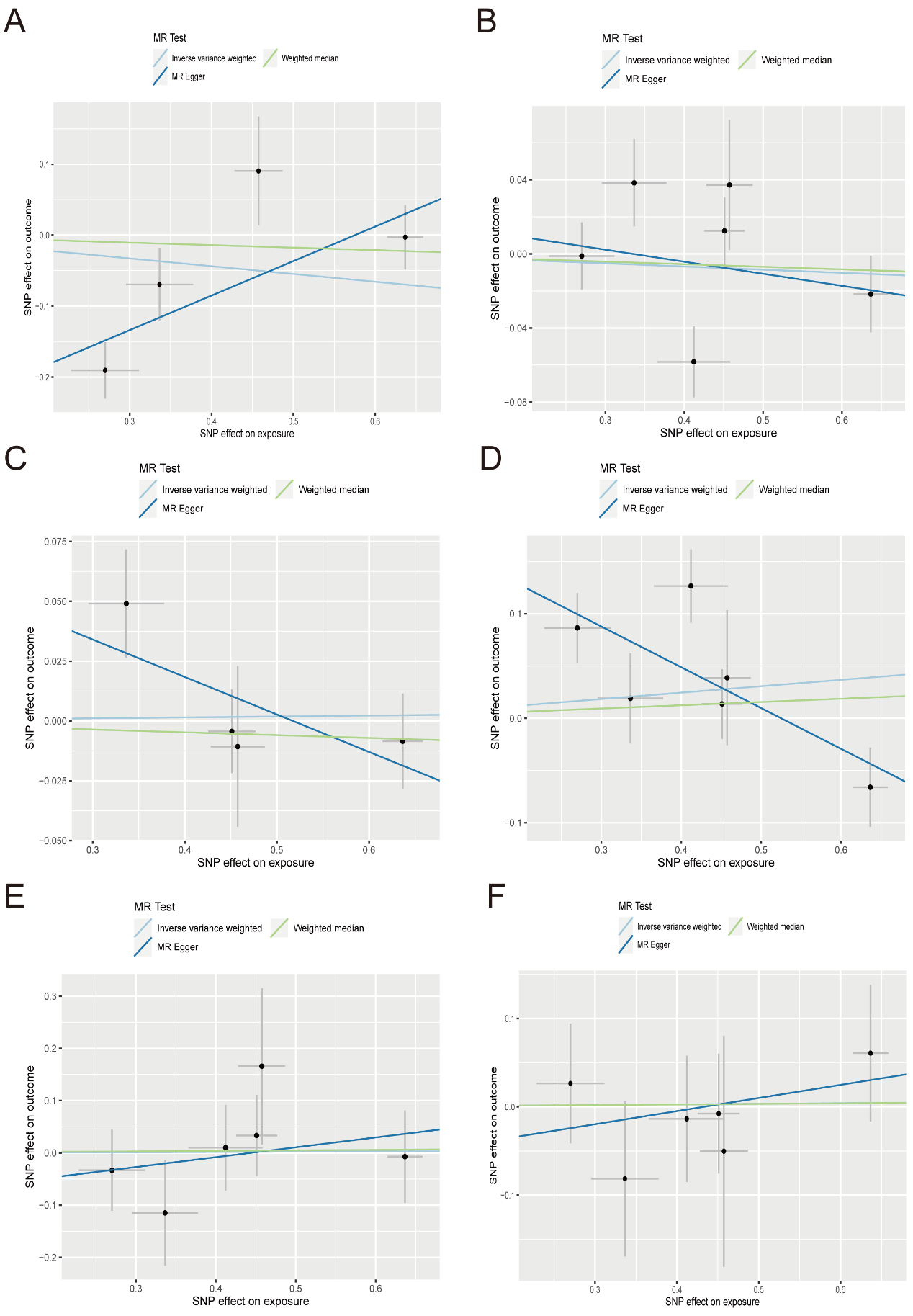


Supplementary Figure 15. Scatter plots for MR analyses of the causal effect of “Gout” on digestive system cancers. (A) esophageal cancer; (B) gastric cancer; (C) colorectal cancer; (D) hepatocellular carcinoma; (E) biliary tract cancer; (F) pancreatic carcinoma.


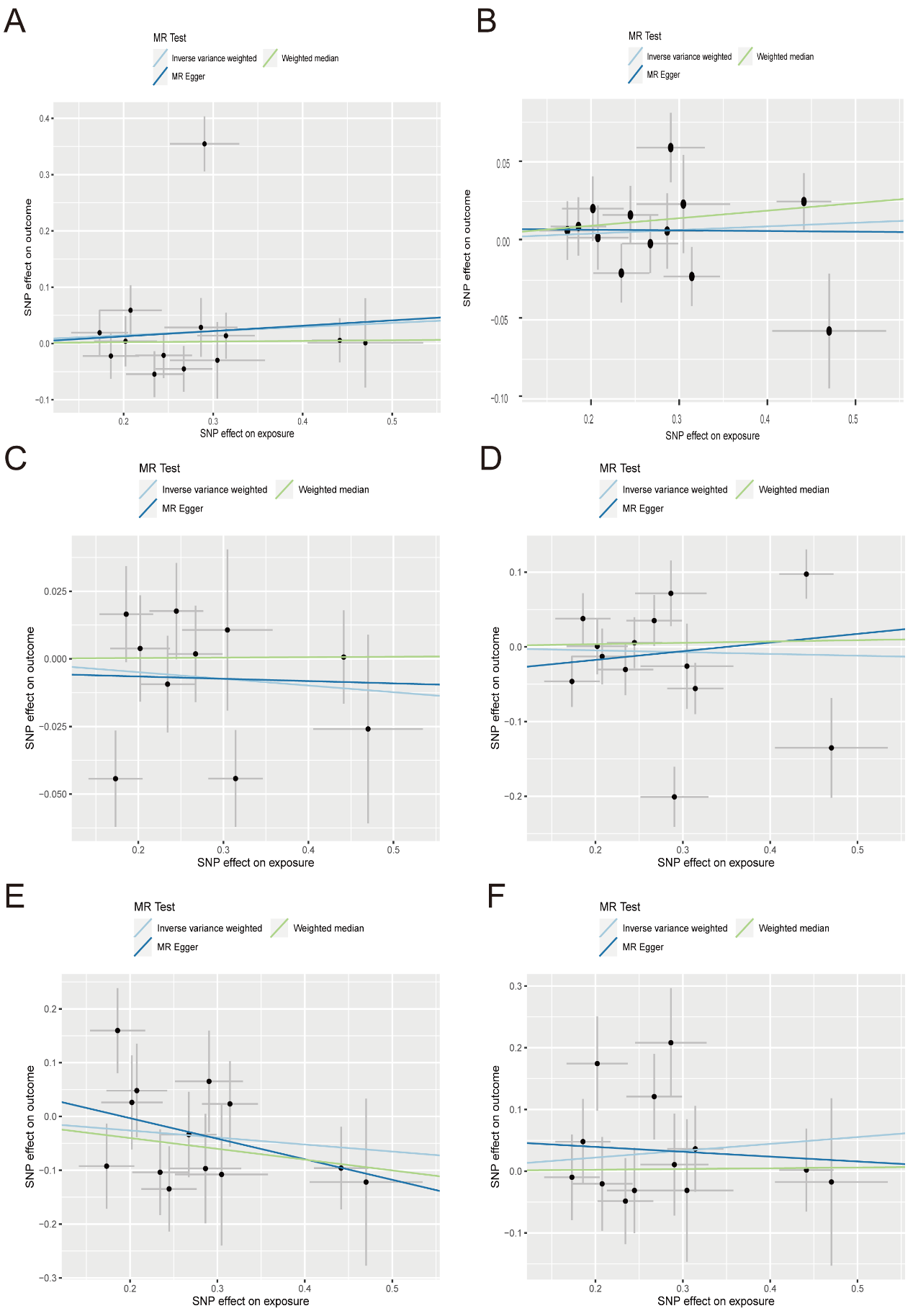


Supplementary Figure 16. Scatter plots for MR analyses of the causal effect of “Graves' disease” on digestive system cancers. (A) esophageal cancer; (B) gastric cancer; (C) colorectal cancer; (D) hepatocellular carcinoma; (E) biliary tract cancer; (F) pancreatic carcinoma.


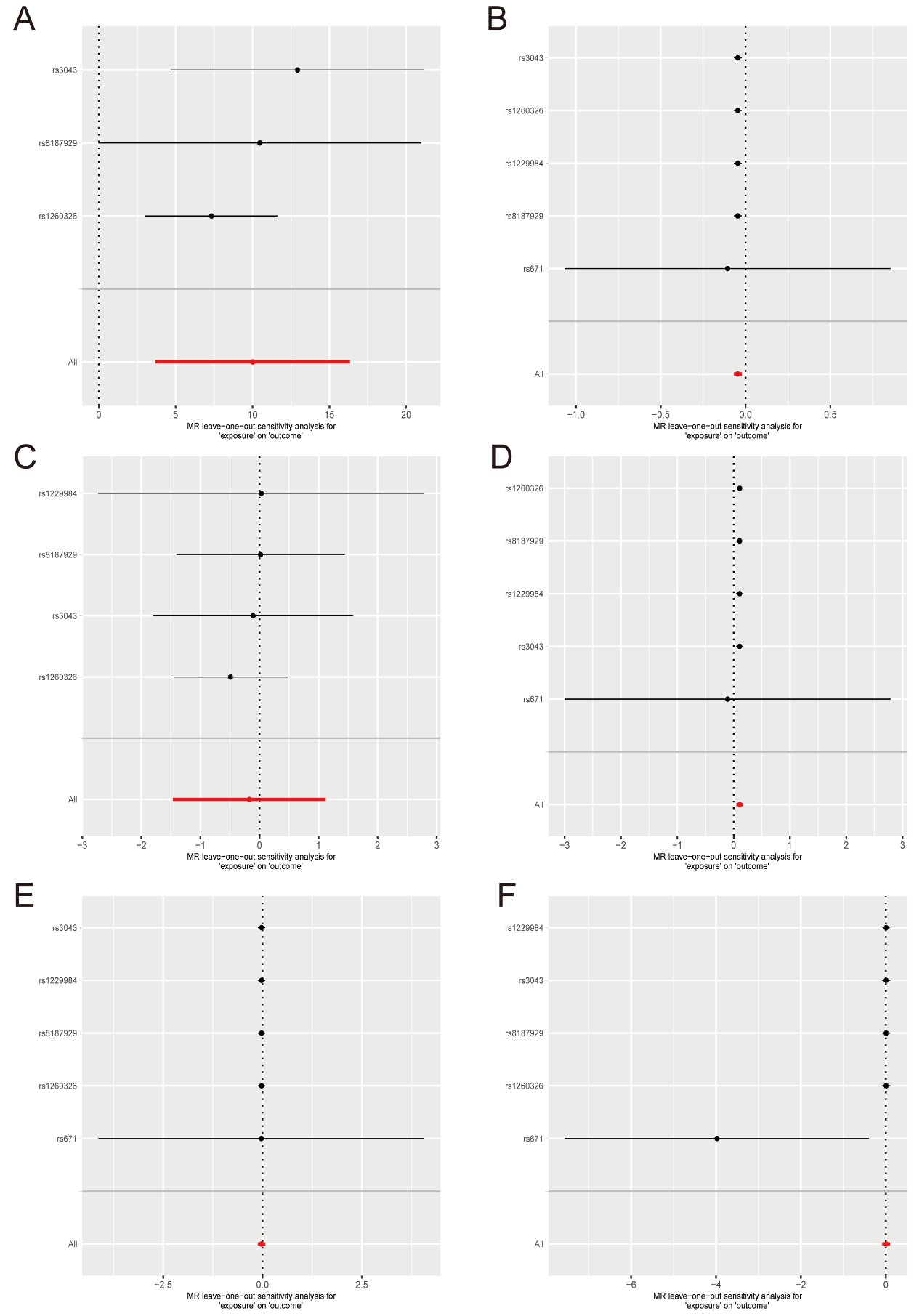


Supplementary Figure 17. Sensitive analysis for MR analyses of the causal effect of “Ever/never drinkers” on digestive system cancers. (A) esophageal cancer; (B) gastric cancer; (C) colorectal cancer; (D) hepatocellular carcinoma; (E) biliary tract cancer; (F) pancreatic carcinoma.


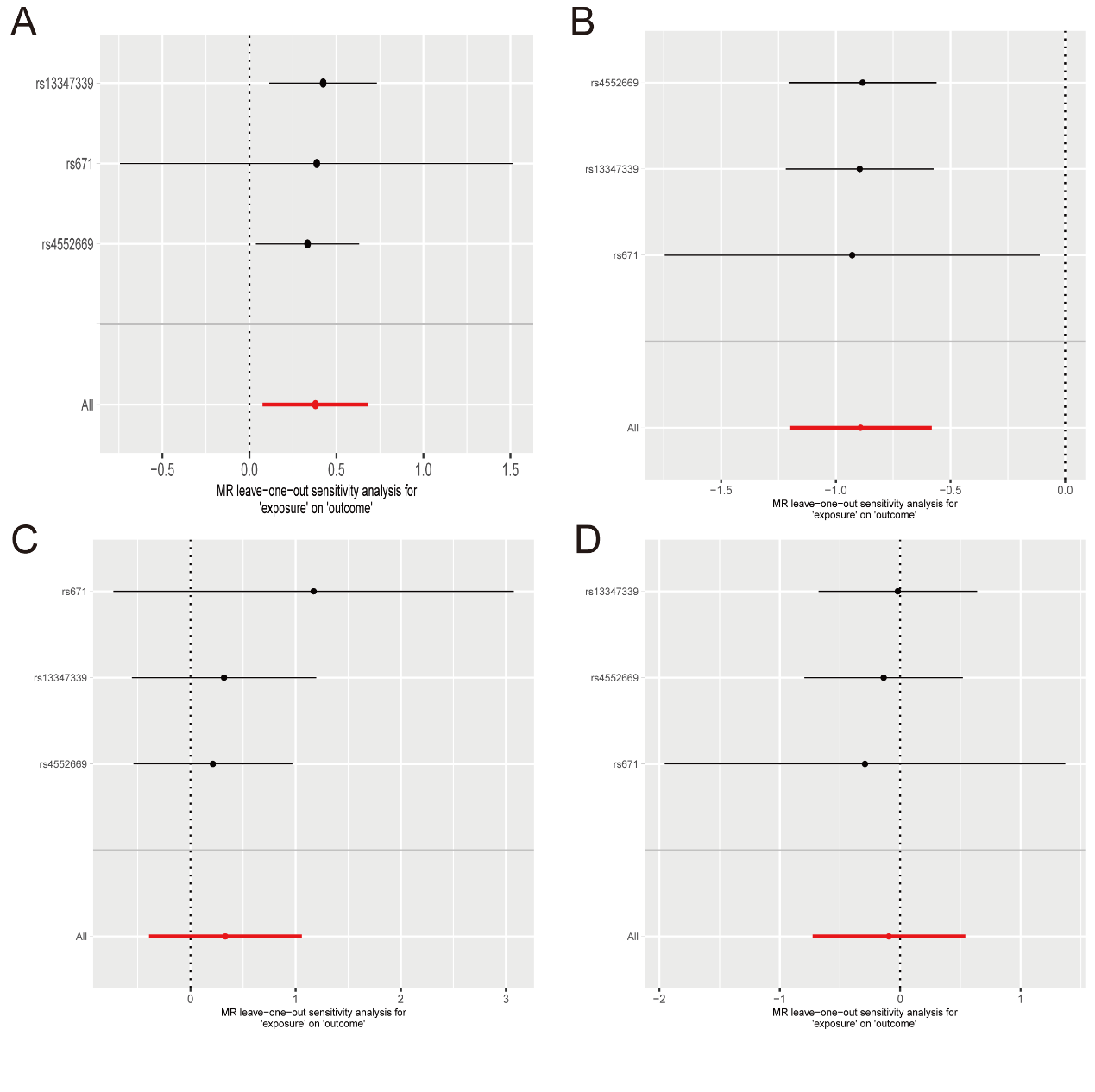
Supplementary Figure 18. Sensitive analysis for MR analyses of the causal effect of “Sweet taste” on digestive system cancers. (A) gastric cancer; (B) hepatocellular carcinoma; (C) biliary tract cancer; (D) pancreatic carcinoma.


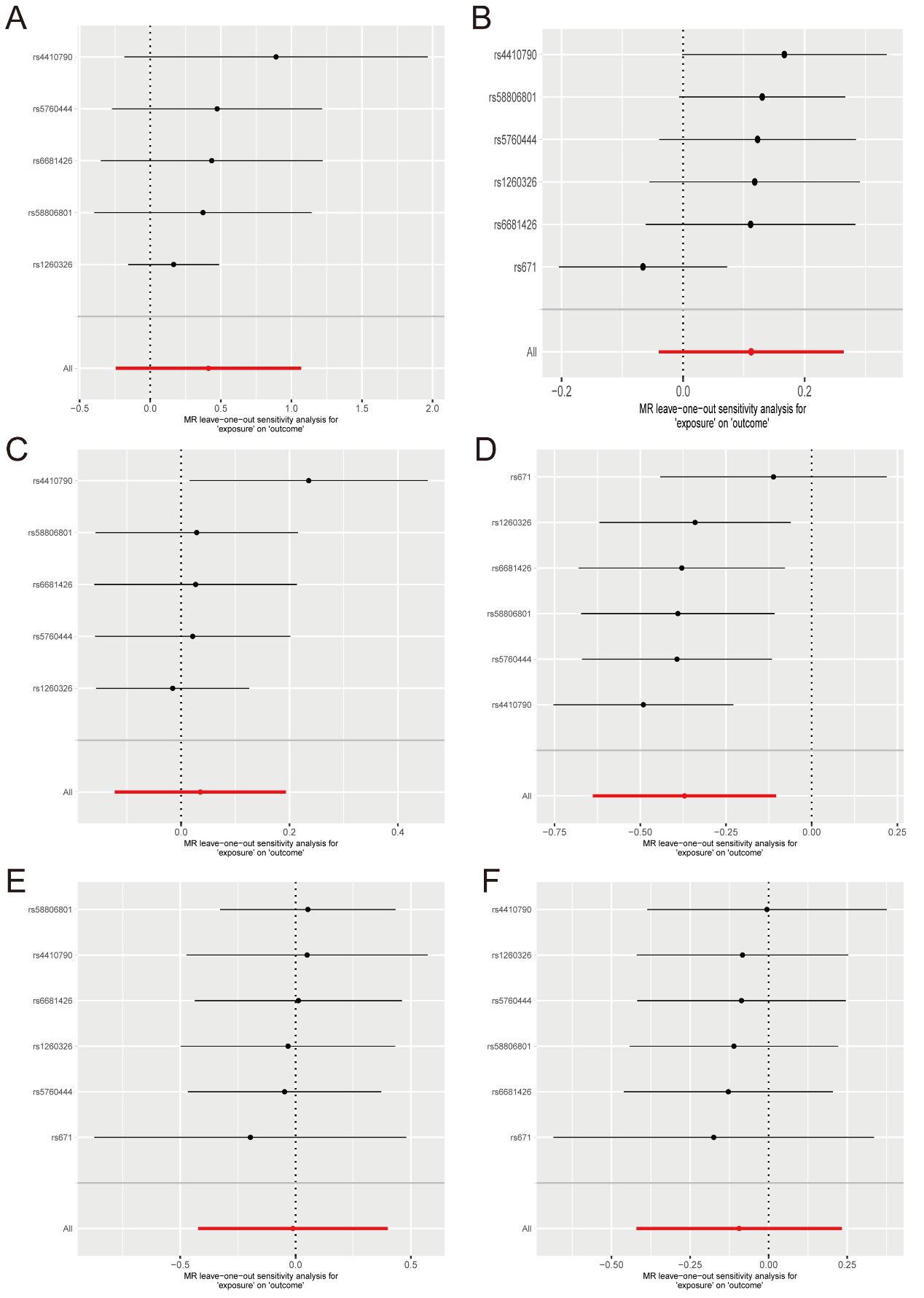


Supplementary Figure 19. Sensitive analysis for MR analyses of the causal effect of “Coffee consumption” on digestive system cancers. (A) esophageal cancer; (B) gastric cancer; (C) colorectal cancer; (D) hepatocellular carcinoma; (E) biliary tract cancer; (F) pancreatic carcinoma.


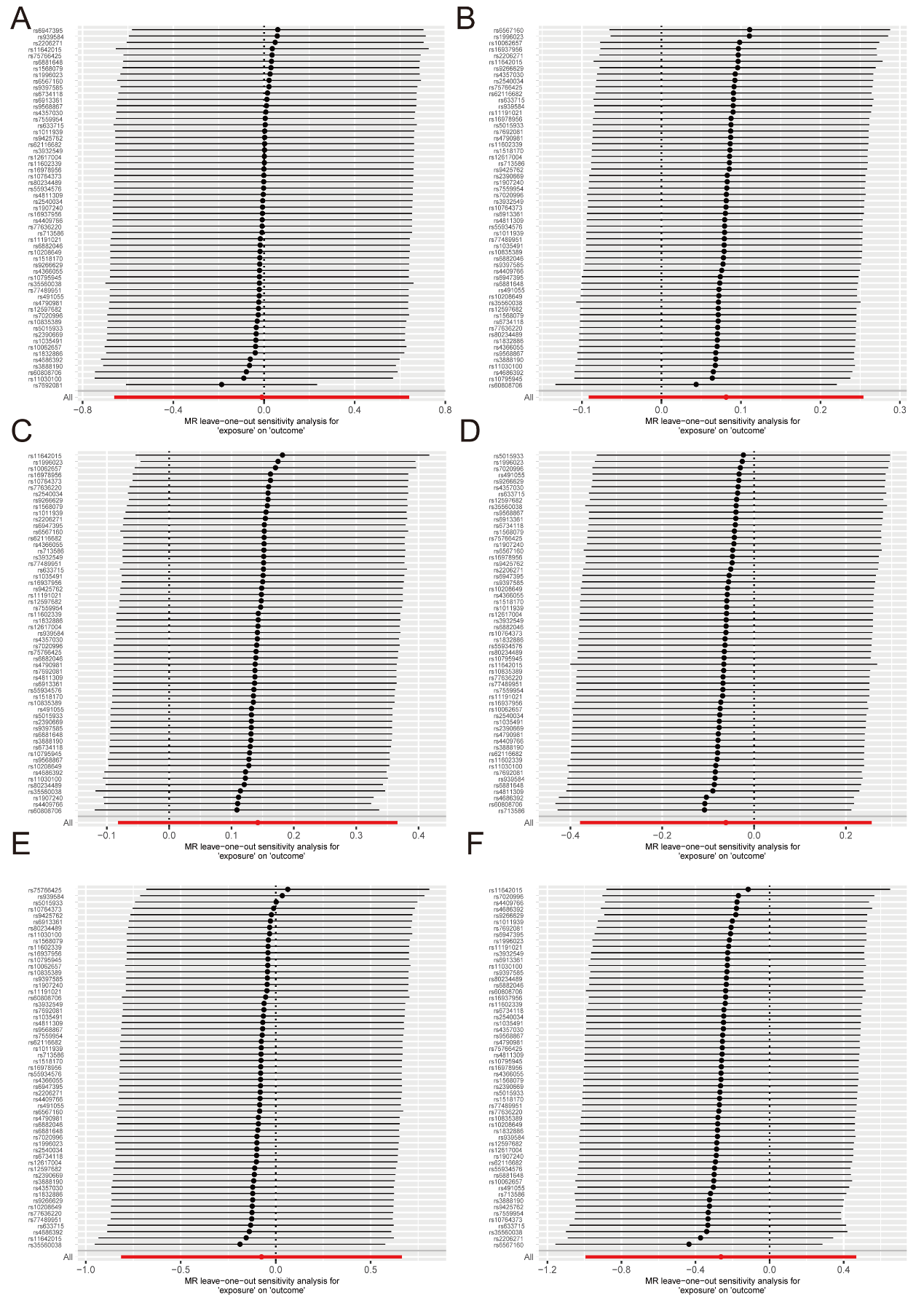


Supplementary Figure 20. Sensitive analysis for MR analyses of the causal effect of “BMI” on digestive system cancers. (A) esophageal cancer; (B) gastric cancer; (C) colorectal cancer; (D) hepatocellular carcinoma; (E) biliary tract cancer; (F) pancreatic carcinoma.


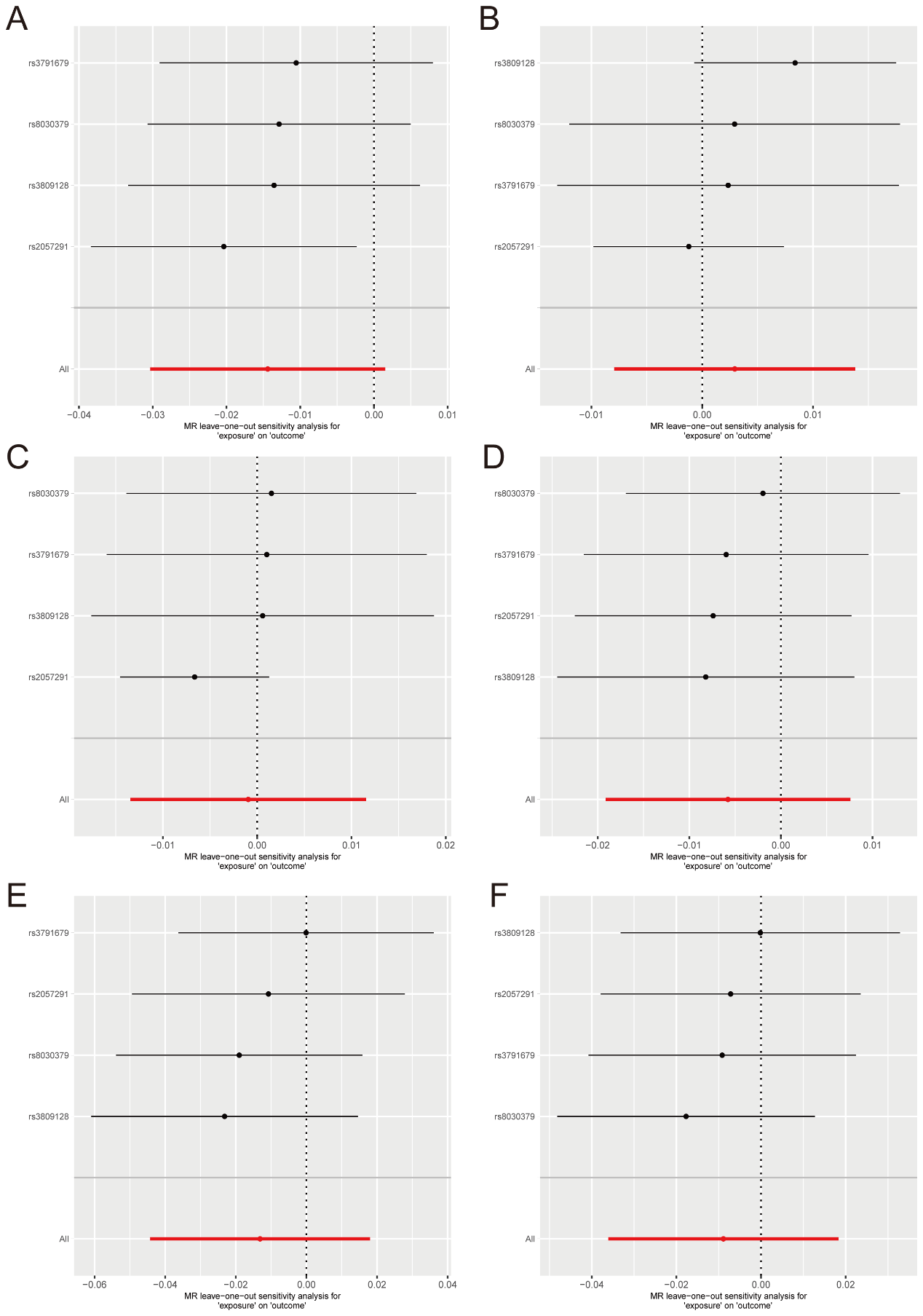


Supplementary Figure 21. Sensitive analysis for MR analyses of the causal effect of “Waist circumference (adjBMI)” on digestive system cancers. (A) esophageal cancer; (B) gastric cancer; (C) colorectal cancer; (D) hepatocellular carcinoma; (E) biliary tract cancer; (F) pancreatic carcinoma.


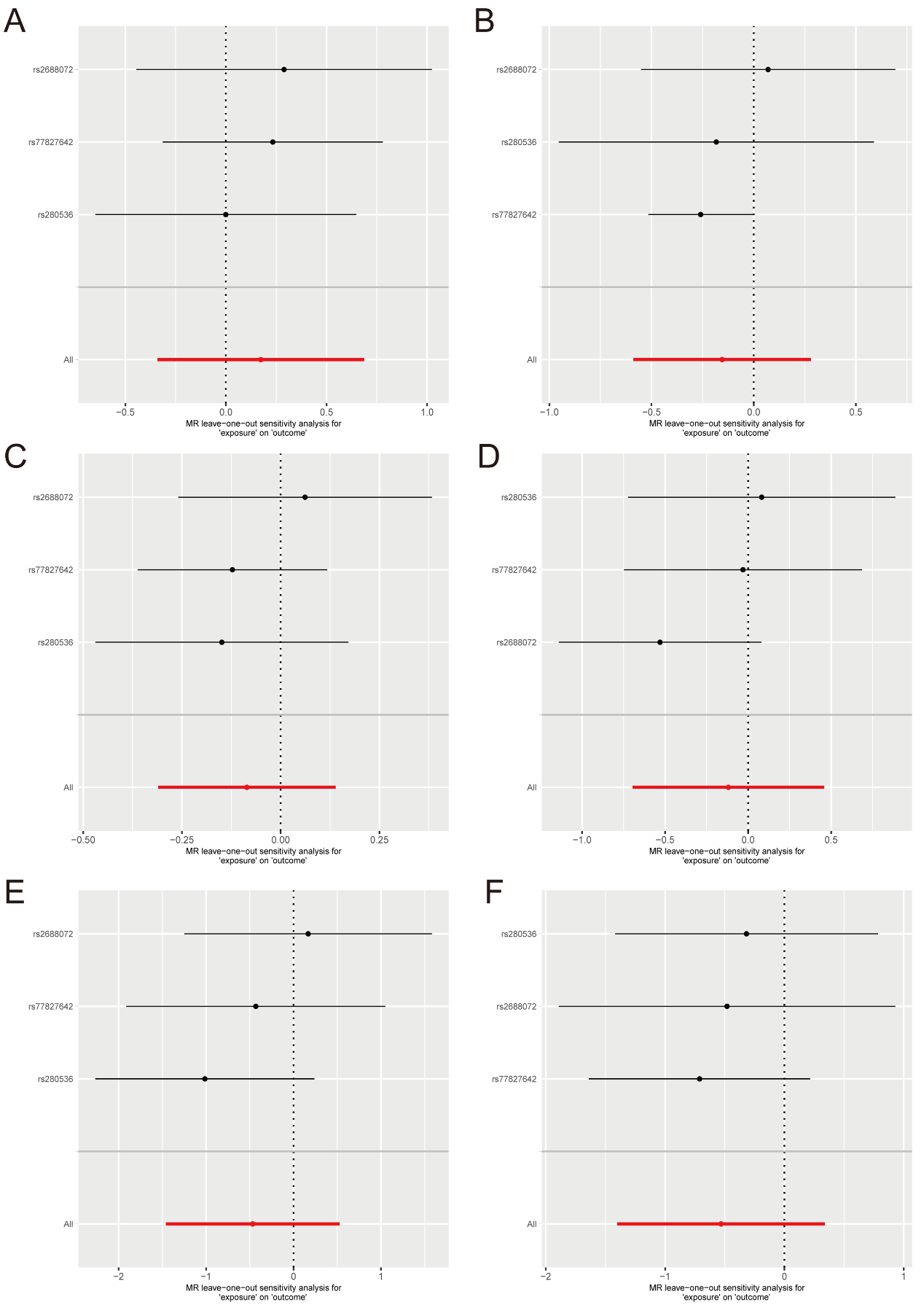


Supplementary Figure 22. Sensitive analysis for MR analyses of the causal effect of “Education status” on digestive system cancers. (A) esophageal cancer; (B) gastric cancer; (C) colorectal cancer; (D) hepatocellular carcinoma; (E) biliary tract cancer; (F) pancreatic carcinoma.


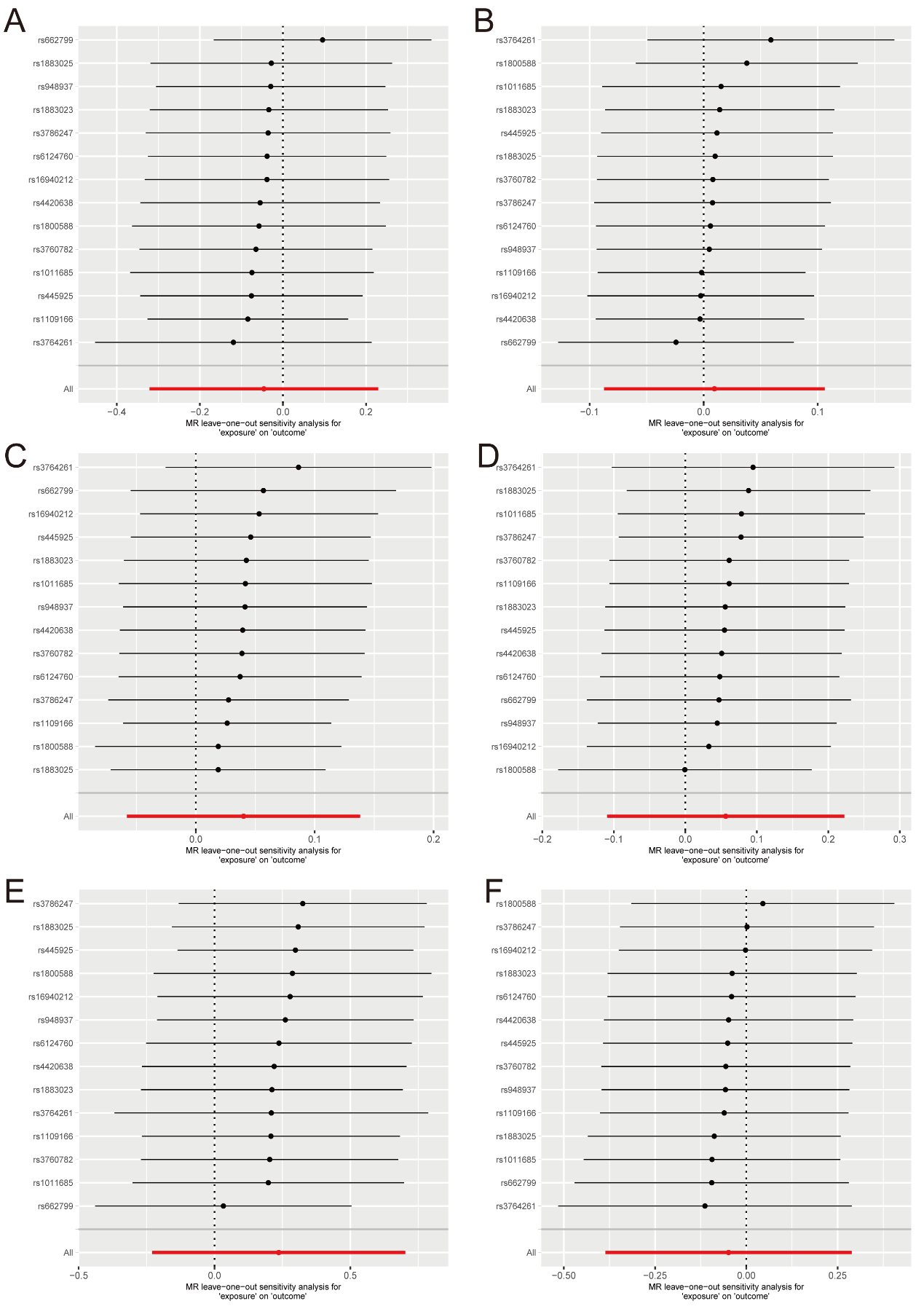


Supplementary Figure 23. Sensitive analysis for MR analyses of the causal effect of “HDL-C” on digestive system cancers. (A) esophageal cancer; (B) gastric cancer; (C) colorectal cancer; (D) hepatocellular carcinoma; (E) biliary tract cancer; (F) pancreatic carcinoma.


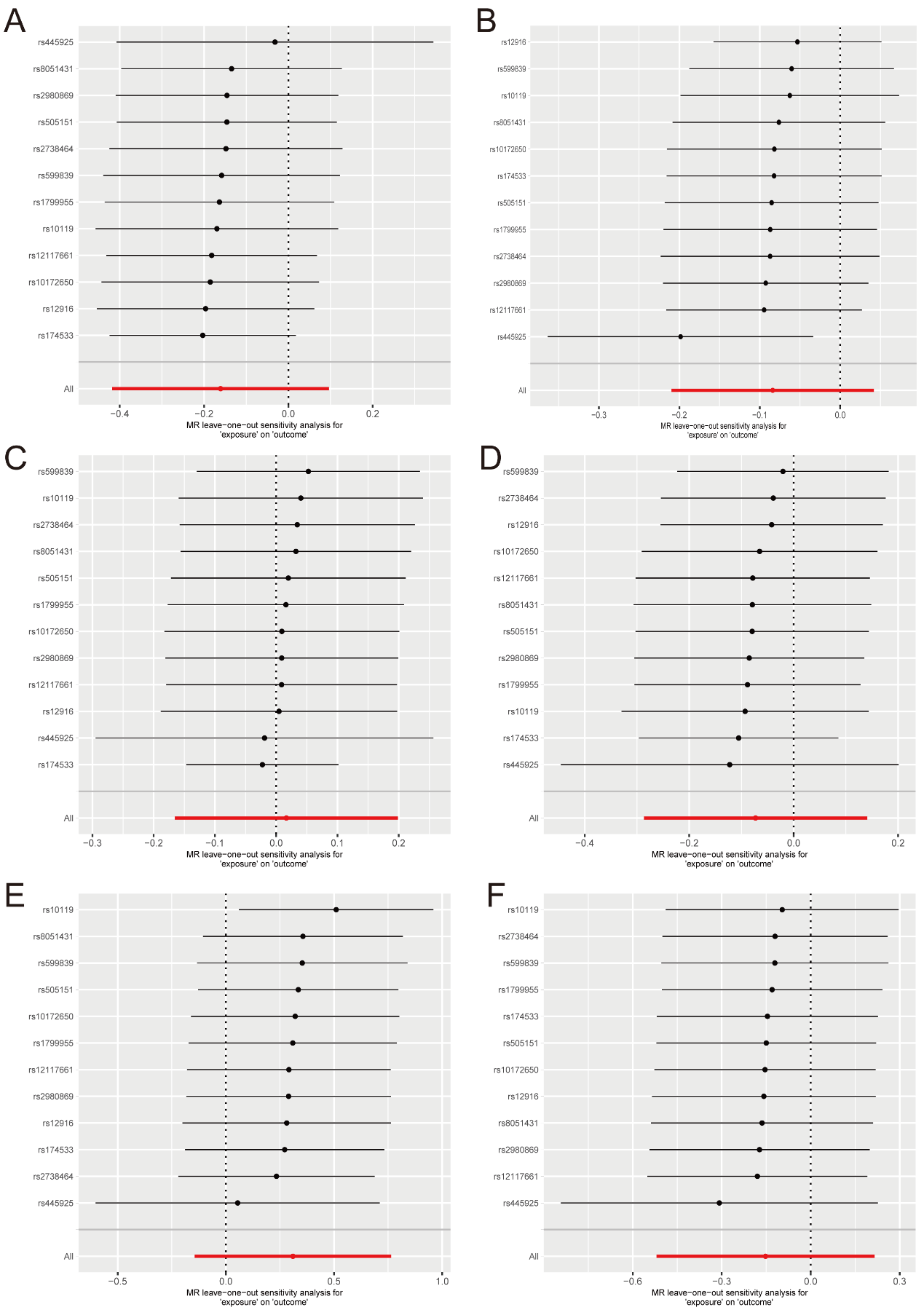


Supplementary Figure 24. Sensitive analysis for MR analyses of the causal effect of “LDL-C” on digestive system cancers. (A) esophageal cancer; (B) gastric cancer; (C) colorectal cancer; (D) hepatocellular carcinoma; (E) biliary tract cancer; (F) pancreatic carcinoma.


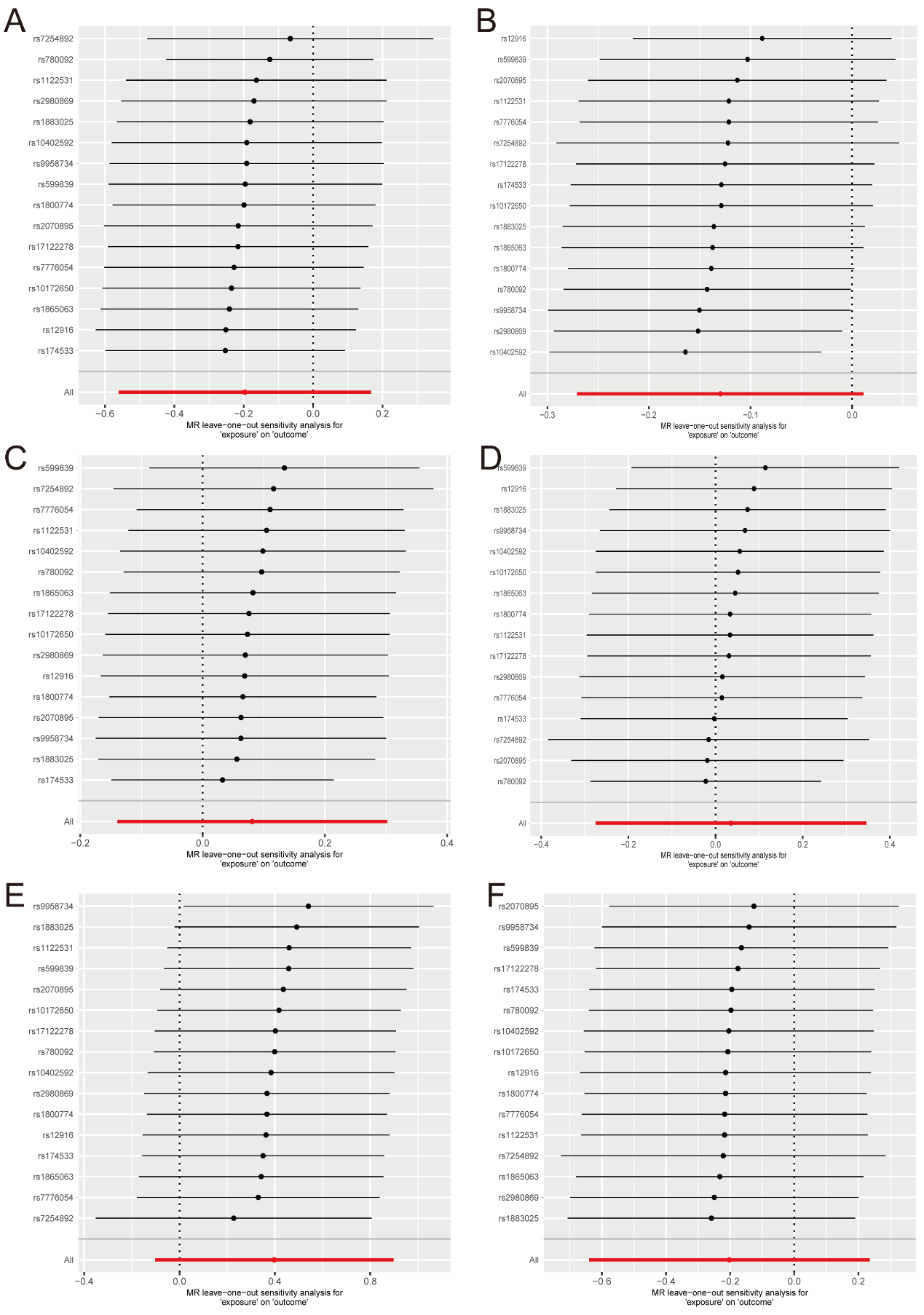


Supplementary Figure 25. Sensitive analysis for MR analyses of the causal effect of “TC-C” on digestive system cancers. (A) esophageal cancer; (B) gastric cancer; (C) colorectal cancer; (D) hepatocellular carcinoma; (E) biliary tract cancer; (F) pancreatic carcinoma.


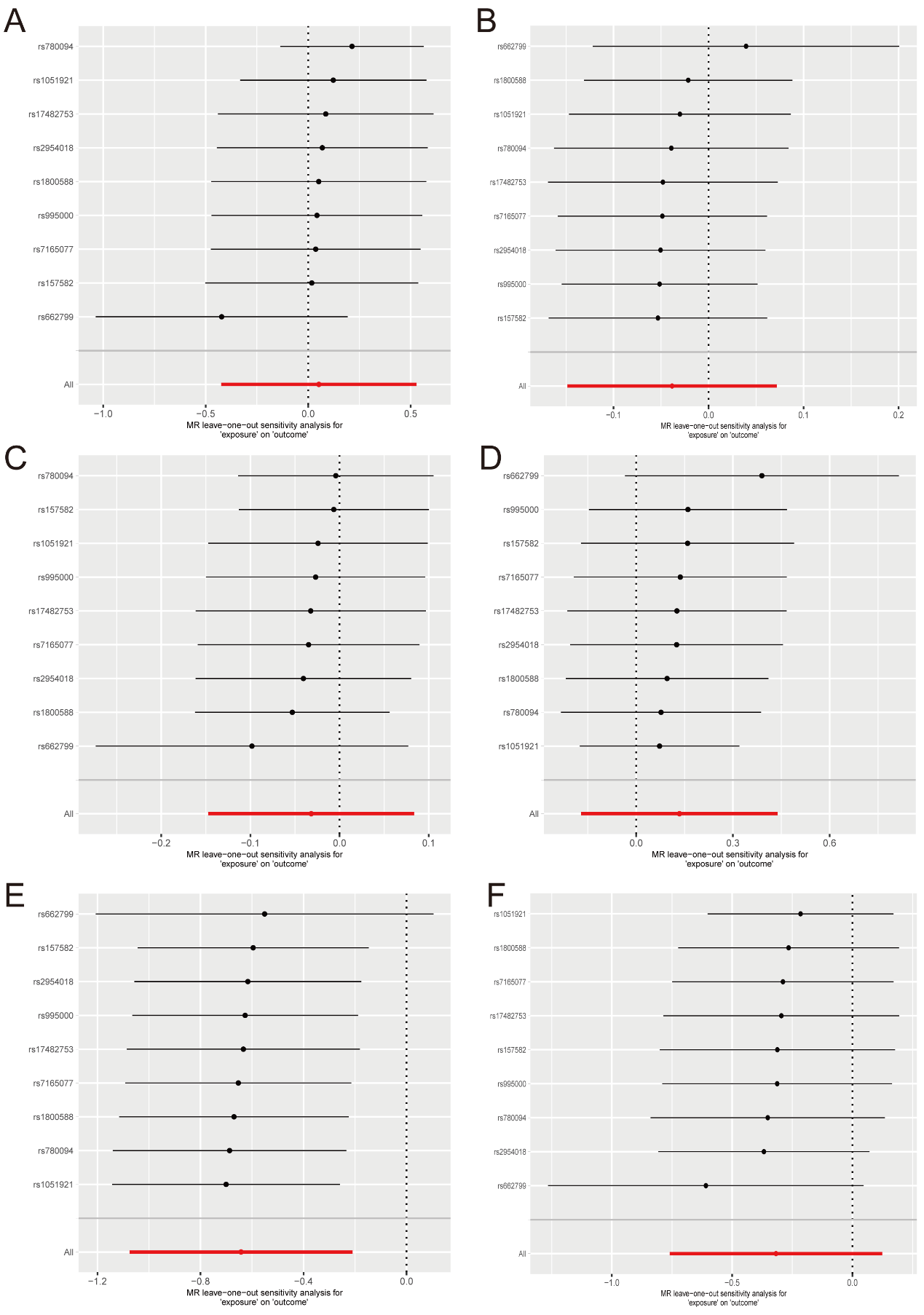


Supplementary Figure 26. Sensitive analysis for MR analyses of the causal effect of “TG-C” on digestive system cancers. (A) esophageal cancer; (B) gastric cancer; (C) colorectal cancer; (D) hepatocellular carcinoma; (E) biliary tract cancer; (F) pancreatic carcinoma.


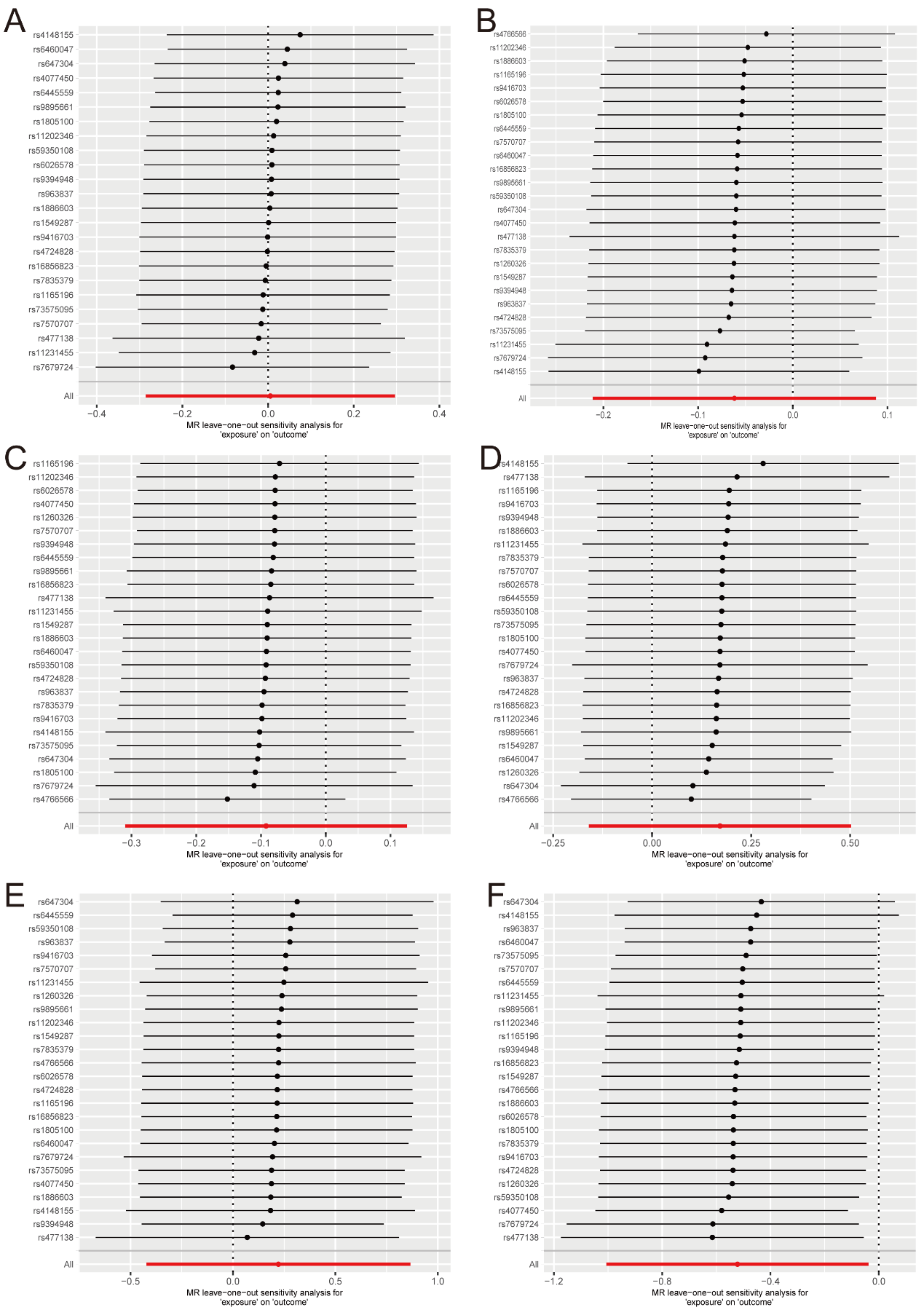


Supplementary Figure 27. Sensitive analysis for MR analyses of the causal effect of “Uric acid” on digestive system cancers. (A) esophageal cancer; (B) gastric cancer; (C) colorectal cancer; (D) hepatocellular carcinoma; (E) biliary tract cancer; (F) pancreatic carcinoma.


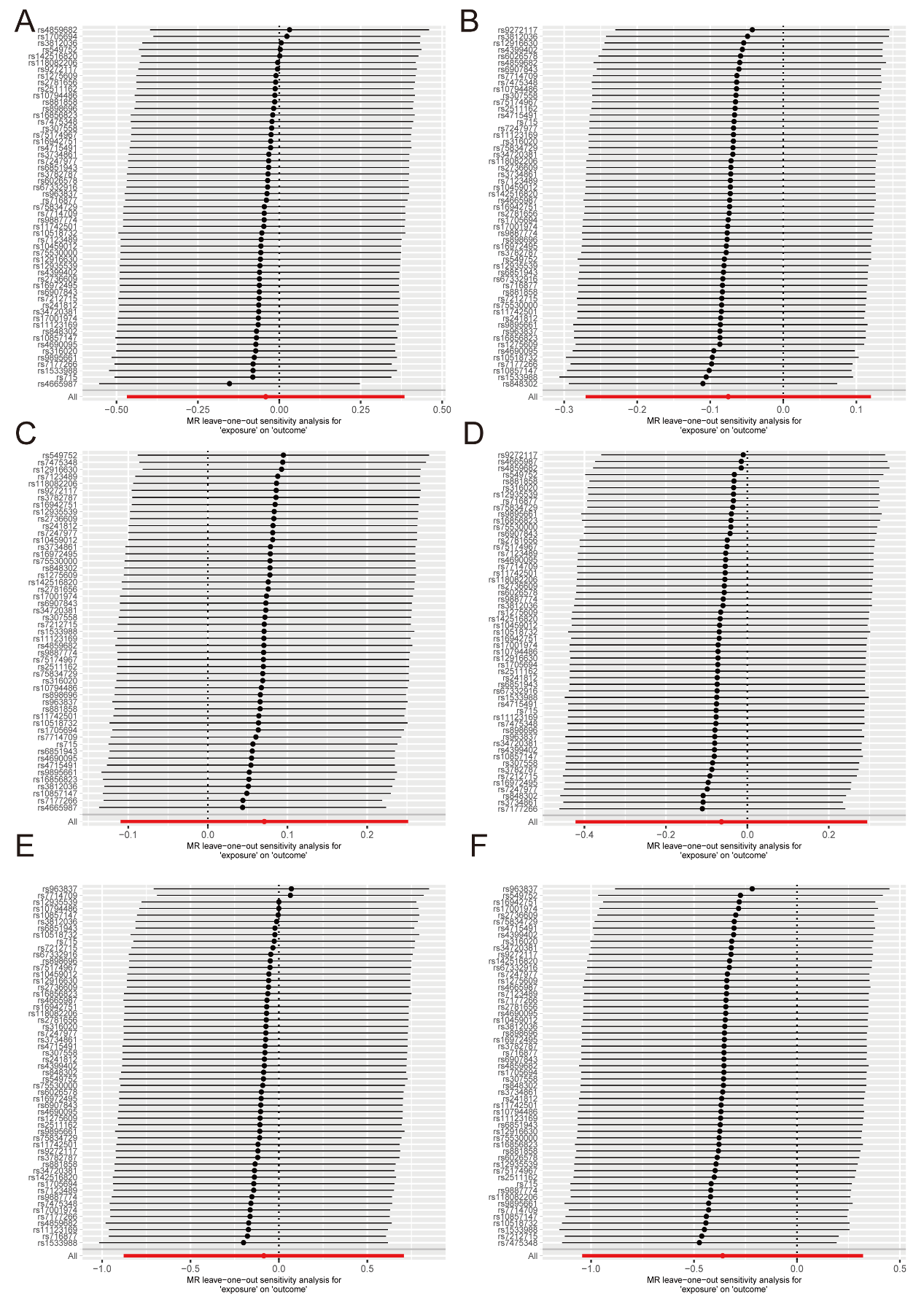


Supplementary Figure 28. Sensitive analysis for MR analyses of the causal effect of “Creatinine” on digestive system cancers. (A) esophageal cancer; (B) gastric cancer; (C) colorectal cancer; (D) hepatocellular carcinoma; (E) biliary tract cancer; (F) pancreatic carcinoma.


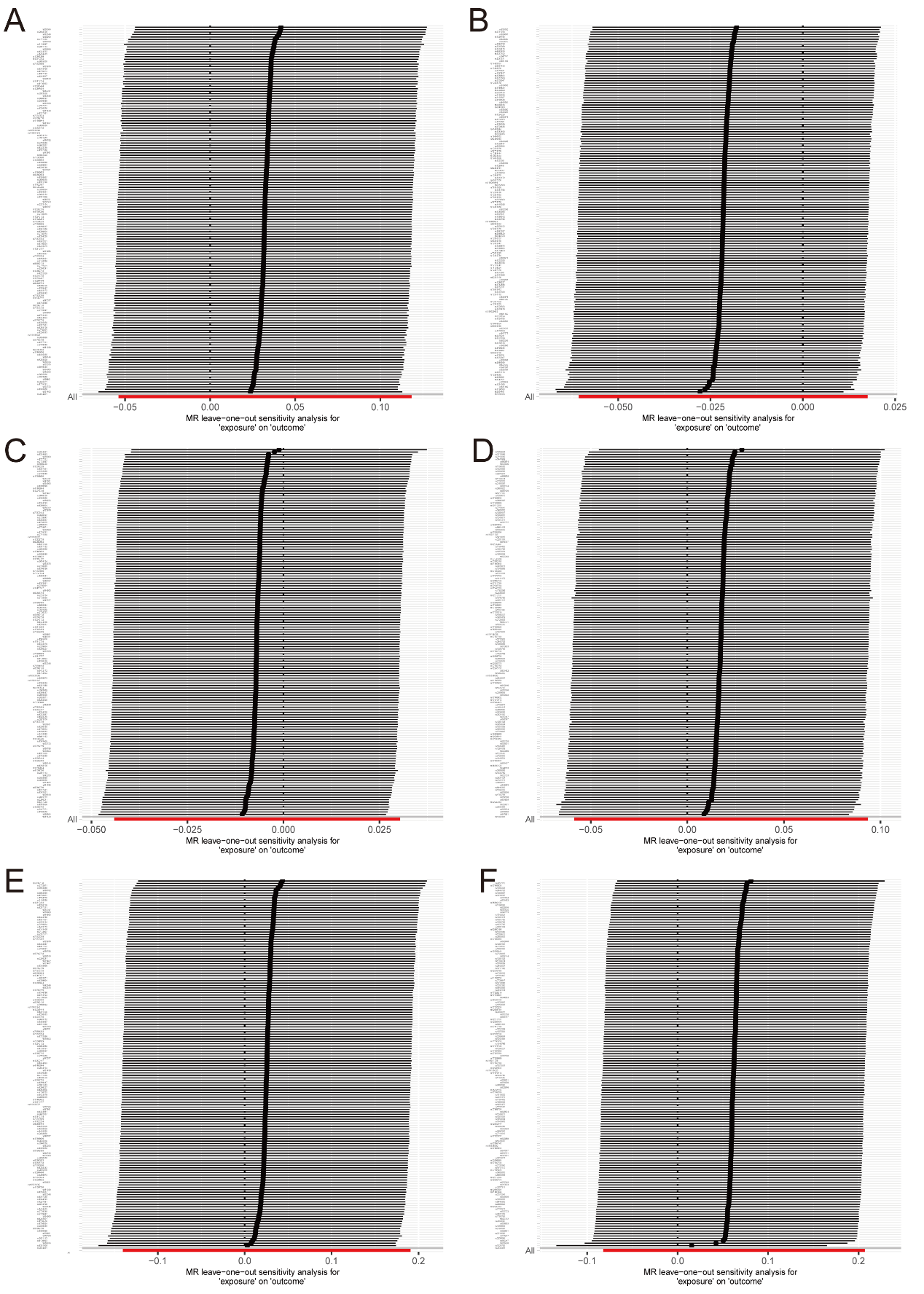


Supplementary Figure 29. Sensitive analysis for MR analyses of the causal effect of “T2D” on digestive system cancers. (A) esophageal cancer; (B) gastric cancer; (C) colorectal cancer; (D) hepatocellular carcinoma; (E) biliary tract cancer; (F) pancreatic carcinoma.


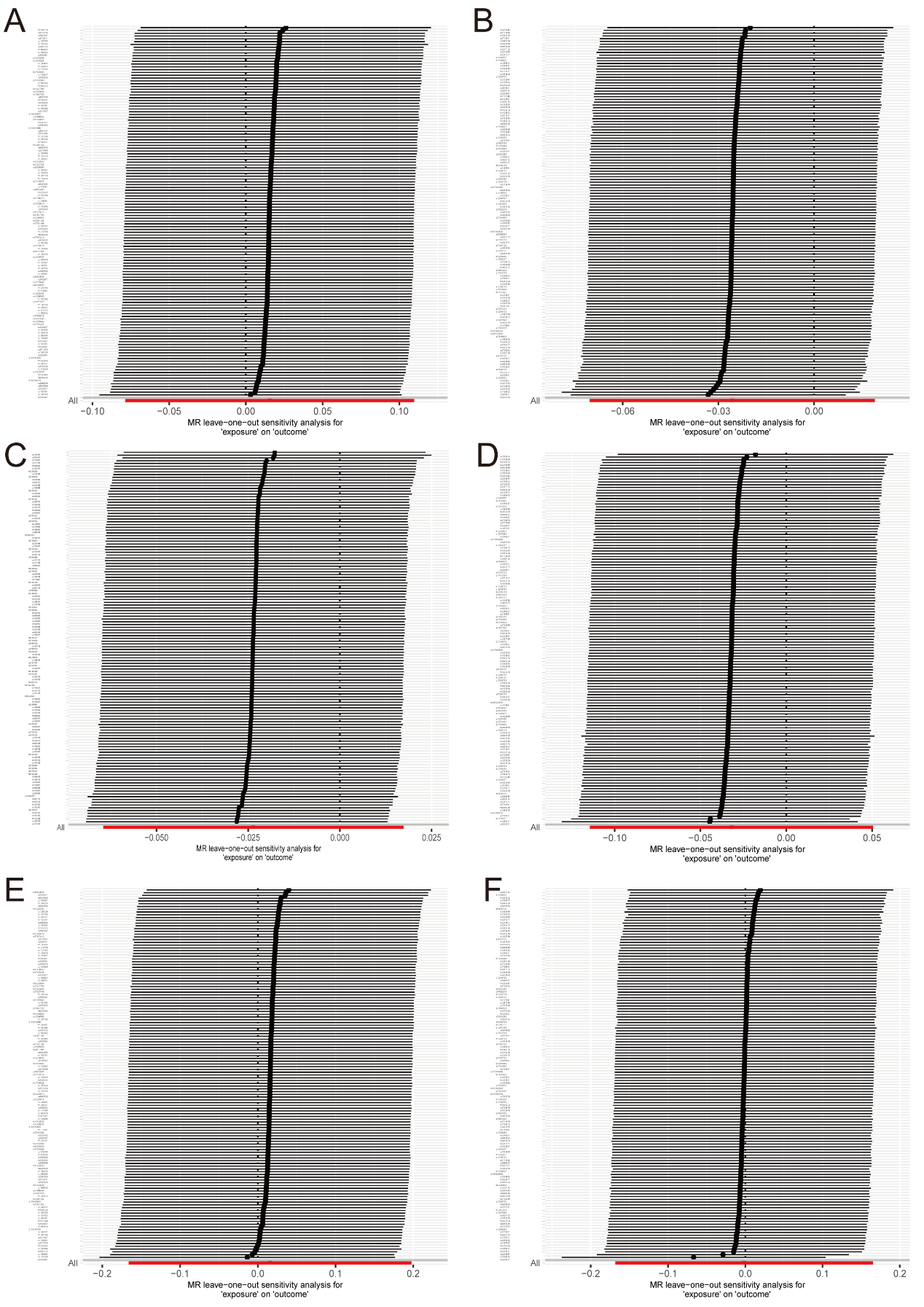


Supplementary Figure 30. Sensitive analysis for MR analyses of the causal effect of “T2D (adjBMI)” on digestive system cancers. (A) esophageal cancer; (B) gastric cancer; (C) colorectal cancer; (D) hepatocellular carcinoma; (E) biliary tract cancer; (F) pancreatic carcinoma.


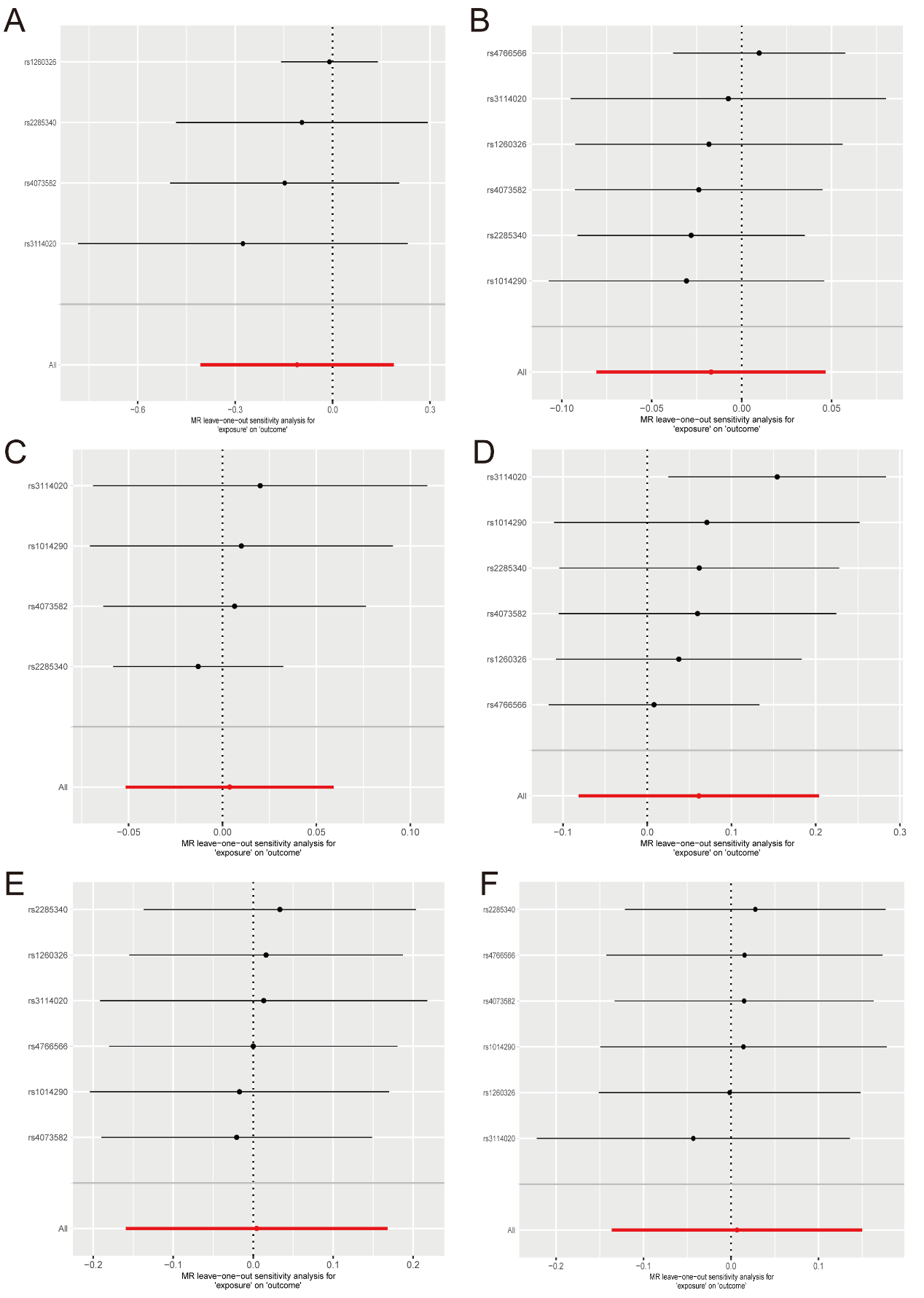


Supplementary Figure 31. Sensitive analysis for MR analyses of the causal effect of “Gout” on digestive system cancers. (A) esophageal cancer; (B) gastric cancer; (C) colorectal cancer; (D) hepatocellular carcinoma; (E) biliary tract cancer; (F) pancreatic carcinoma.


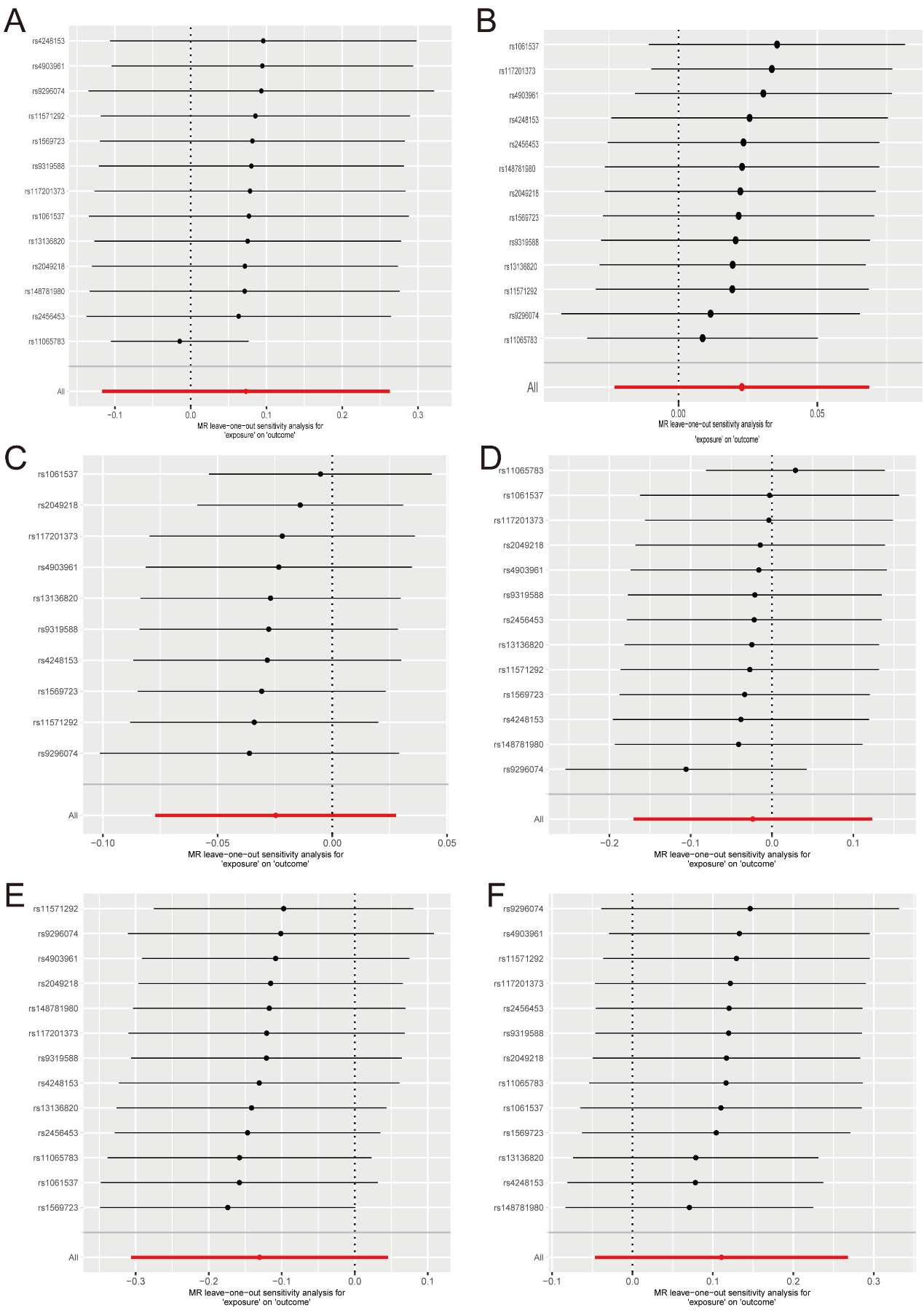


Supplementary Figure 32. Sensitive analysis for MR analyses of the causal effect of “Graves' disease” on digestive system cancers. (A) esophageal cancer; (B) gastric cancer; (C) colorectal cancer; (D) hepatocellular carcinoma; (E) biliary tract cancer; (F) pancreatic carcinoma.
